# Enzymatic Synthesis of Variediene Analogs

**DOI:** 10.1002/chem.202200095

**Published:** 2022-02-09

**Authors:** Lin‐Fu Liang, Jeroen S. Dickschat

**Affiliations:** ^1^ Kekulé-Institute for Organic Chemistry and Biochemistry University of Bonn Gerhard-Domagk-Straße 1 53121 Bonn Germany

**Keywords:** configuration determination, enzymes, isotopes, substrate analogs, terpenoids

## Abstract

Five analogs of dimethylallyl diphosphate (DMAPP) with additional or shifted Me groups were converted with isopentenyl diphosphate (IPP) and the fungal variediene synthase from *Aspergillus brasiliensis* (AbVS). These enzymatic reactions resulted in the formation of several new terpene analogs that were isolated and structurally characterised by NMR spectroscopy. Several DMAPP analogs showed a changed reactivity giving access to compounds with unusual skeletons. Their formation is mechanistically rationalised and the absolute configurations of all obtained compounds were determined through a stereoselective deuteration strategy, revealing absolute configurations that are analogous to that of the natural enzyme product variediene.

## Graphical abstract

**Five DMAPP analogs** with additional carbon atoms or with changed methylation patterns were converted with IPP and the bifunctional variediene synthase from *Aspergillus brasiliensis* AbVS to yield diterpene analogs with unusual skeletons. The DMAPP analogs opened new reaction pathways through the stabilisation of cationic charges at alternative carbon atoms that are naturally not observed.

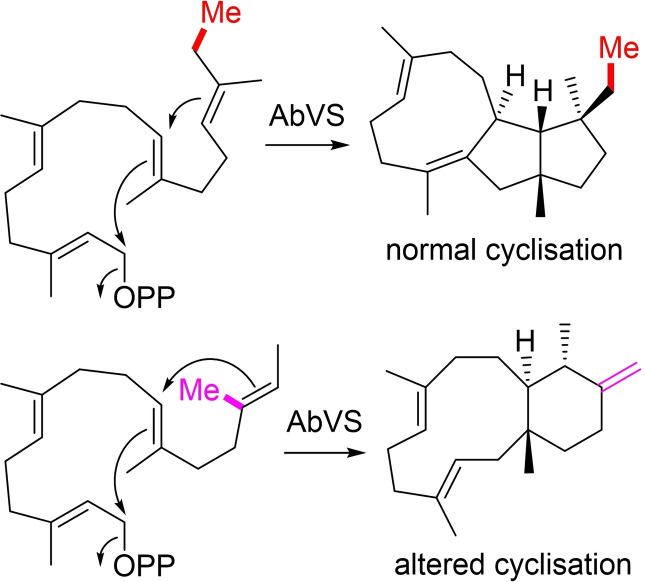

## Introduction

Terpene synthases (TPSs) are remarkable biocatalysts that convert structurally simple, acyclic and achiral oligoprenyl diphosphates into often highly complex, (poly)cyclic and chiral terpenes. These transformations are achieved by the Mg^2+^ dependent type I TPSs through the abstraction of diphosphate which generates a highly reactive allyl cation intermediate.[^1^, ^2^, ^3^] This can undergo a cyclisation cascade involving typical elementary steps of carbocation chemistry such as ring closures by intramolecular attack of an alkene to a cationic center, Wagner‐Meerwein rearrangements, and hydride or proton shifts. The cascade reactions are usually terminated by deprotonation to yield a terpene hydrocarbon or by nucleophilic attack of water to result in a terpene alcohol. The enzyme provides a hydrophobic cavity for these reactions that is contoured mostly by non‐polar aliphatic and aromatic residues that force the substrate into a reactive conformation that determines the structure of the product.[^4^, ^5^, ^6^, ^7^] Additional TPS functions span the stabilisation of cationic intermediates e. g. through cation‐π interactions[^5^, ^8^] and participation in general acid/base catalysis for deprotonations and reprotonations of neutral intermediates. Based on a combined experimental and theoretical study we recently showed that in bacterial selinadiene synthase main chain carbonyl oxygens and an active site water are relevant for such deprotonation‐reprotonation sequences.^[9]^

It is well known that prenyltransferases can use analogs of the terpene monomers dimethylallyl diphosphate (DMAPP) and isopentenyl diphosphate (IPP).^[10]^ Recent research has demonstrated that TPSs convert various substrate analogs^[11]^ including halogenated precursors,[^12^, ^13^, ^14^, ^15^] hydroxylated oligoprenyl diphosphates,[^16^, ^17^] epoxides,^[18]^ ketones,[^18^, ^19^] substrates with heteroatoms inserted into the chain,[^20^, ^21^] with altered methylation pattern,[^15^, ^19^, ^22^, ^23^] shifted[^18^, ^19^] or saturated double bonds[^18^, ^24^, ^25^] into terpenoid compounds. This approach is conceptually highly interesting, because the required chemical synthesis of such (acyclic and achiral) substrate analogs is much easier than a stereoselective synthesis of the (poly)cyclic enzyme products. Among class I diterpene synthases only taxadiene synthase from *Taxus brevifolia* ^[12]^ and catenul‐14‐en‐6‐ol synthase from *Catenulispora acidiphila* ^[25]^ have been investigated for their tolerance towards non‐natural substrate analogs. In order to expand this knowledge, we have now investigated the potential of the fungal variediene synthase from *Aspergillus brasiliensis* (AbVS),^[26]^ a homolog of the earlier characterised variediene synthase from *Emericella variecolor* (EvVS),^[27]^ to convert analogs of DMAPP through in vitro reactions with IPP.

## Results and Discussion

**Scheme 1:**
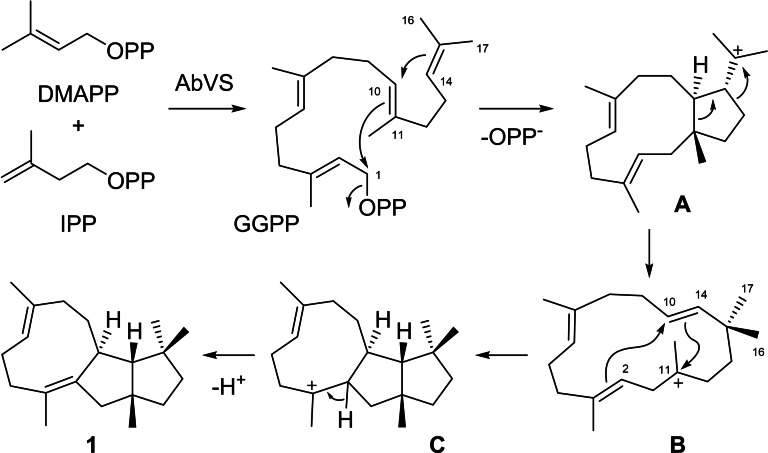
Conversion of DMAPP and IPP into **1** by AbVS.

AbVS is a bifunctional enzyme with a prenyltransferase (PT) and a terpene cyclase (TC) domain that naturally converts DMAPP and IPP through geranylgeranyl diphosphate (GGPP) into variediene (**1**, Scheme 1). Based on the results from labelling experiments[^26^, ^27^] and computational studies,[^28^, ^29^] the reactions from GGPP proceed through 1,11‐10,14 cyclisation to **A**, followed by Wagner‐Meerwein rearrangement and ring opening to **B** with a specific stereochemical course that places Me16 in the *pro*‐*S* and Me17 in the *pro*‐*R* position. A reverse 14,11 ring closure with simultaneous 2,10 cyclisation leads to **C** that is the precursor of **1** by deprotonation.

**Scheme 2:**
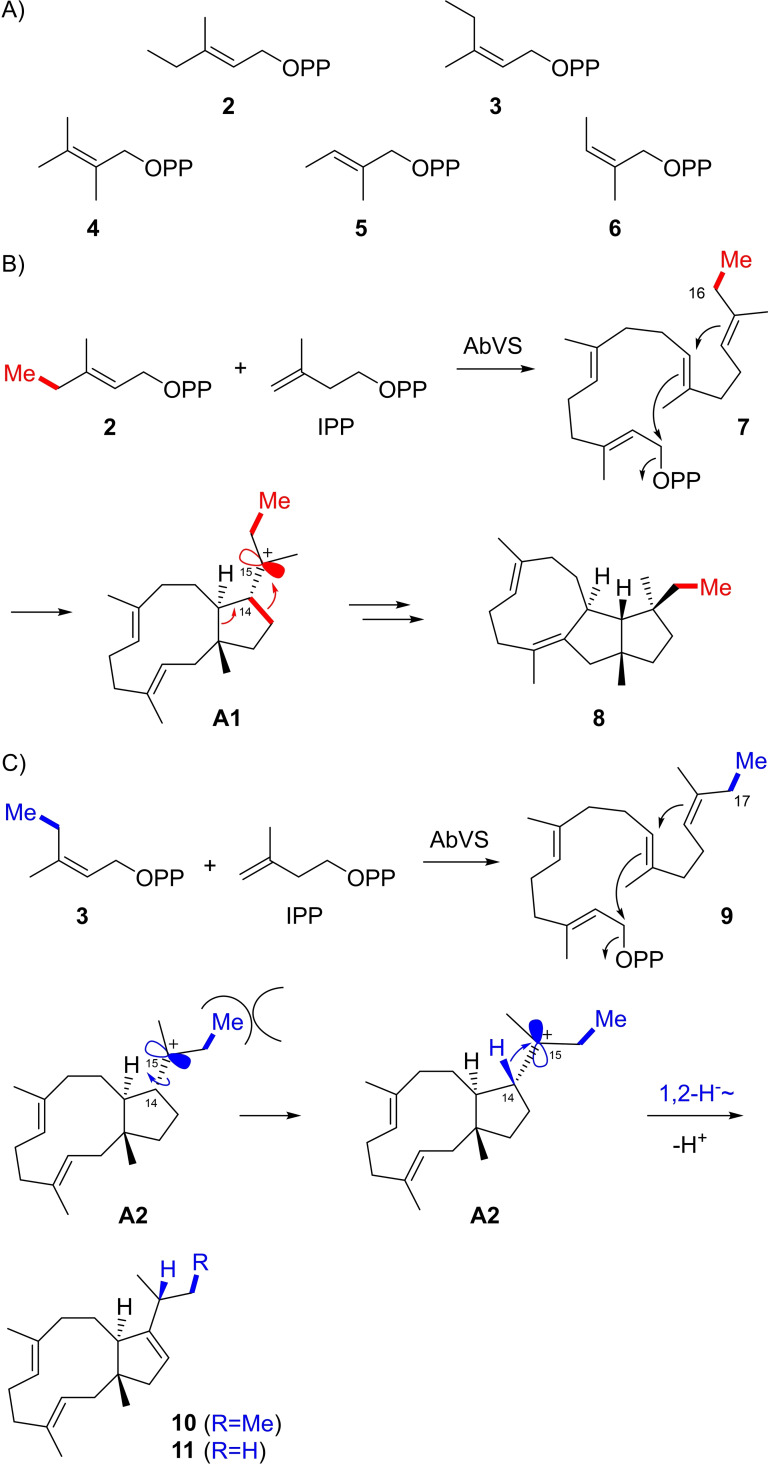
Enzymatic conversions of DMAPP analogs with AbVS. A) DMAPP analogs used in this study, B) AbVS catalysed conversion of **2** and IPP into **8** and C) of **3** and IPP into **10**. The blue arrow at intermediate **A2** indicates the rotation around the C14−C15 bond hypothetically caused by a steric clash in the active site of AbVS that is not relevant for **A1**. Compound **11** is the known natural product dolabella‐3,7,12‐triene.

For enzymatic conversions with AbVS, the DMAPP analogs (*E*)‐3‐methylpent‐2‐enyl diphosphate (**2**), (*Z*)‐3‐methylpent‐2‐enyl diphosphate (**3**), 2,3‐dimethylbut‐2‐enyl diphosphate (**4**), (*E*)‐2‐methylbut‐2‐enyl diphosphate (**5**) and (*Z*)‐2‐methylbut‐2‐enyl diphosphate (**6**) were used (Scheme 2A). Compounds **4** and **5** were synthesised as reported before,^[19]^ and the synthesis of **2**, **3** and **6** is shown in Scheme S1. The incubation of **2** and IPP with AbVS resulted in the formation of **8**, while the enzyme reaction with **3** and IPP yielded **10** (Scheme 2B and 2C). Both compounds were isolated and structurally characterised by NMR spectroscopy (Tables S1 and S2, Figures S1–S16), revealing that compound **8** (homovariediene) is an analog of variediene methylated at C16. Its formation can be explained by the same sequence of steps as for the biosynthesis of **1**, suggesting that the additional carbon introduced from **2** does not cause a change in the reactivity of the intermediates towards **8**. In contrast, for compound **10** the cyclisation cascade is disturbed at intermediate **A2**, the analog of **A** in Scheme 1, that does not react in a ring expansion and ring opening to a **B** analog, but instead reacts through 1,2‐hydride shift and deprotonation to **10**. The altered reactivity in **A2** is likely a consequence of a steric repulsion in the enzyme's active site that forces the *sec*‐Bu group in **A2** with rotation around the C14−C15 bond into a different conformation as in **A1** (and as for the *i*Pr group in **A**). While in **A1** (and **A**) the empty p‐orbital at C15 may be parallel to the σ‐orbital of the C13−C14 bond, the conformational change in **A2** may lead to a parallel orientation of the empty p‐orbital at C15 and the σ‐orbital for the C14−H bond, with the consequence of a downstream 1,2‐hydride shift instead of a Wagner‐Meerwein rearrangement. A compound with the same skeleton as **10**, but without the additional Me group shown in blue, has first been obtained by total synthesis^[30]^ and was subsequently reported as the natural product dolabella‐3,7,12‐triene (**11**) from the brown alga *Dilophus spiralis*.^[31]^ Therefore, **10** was designated homodolabellatriene.

**Scheme 3:**
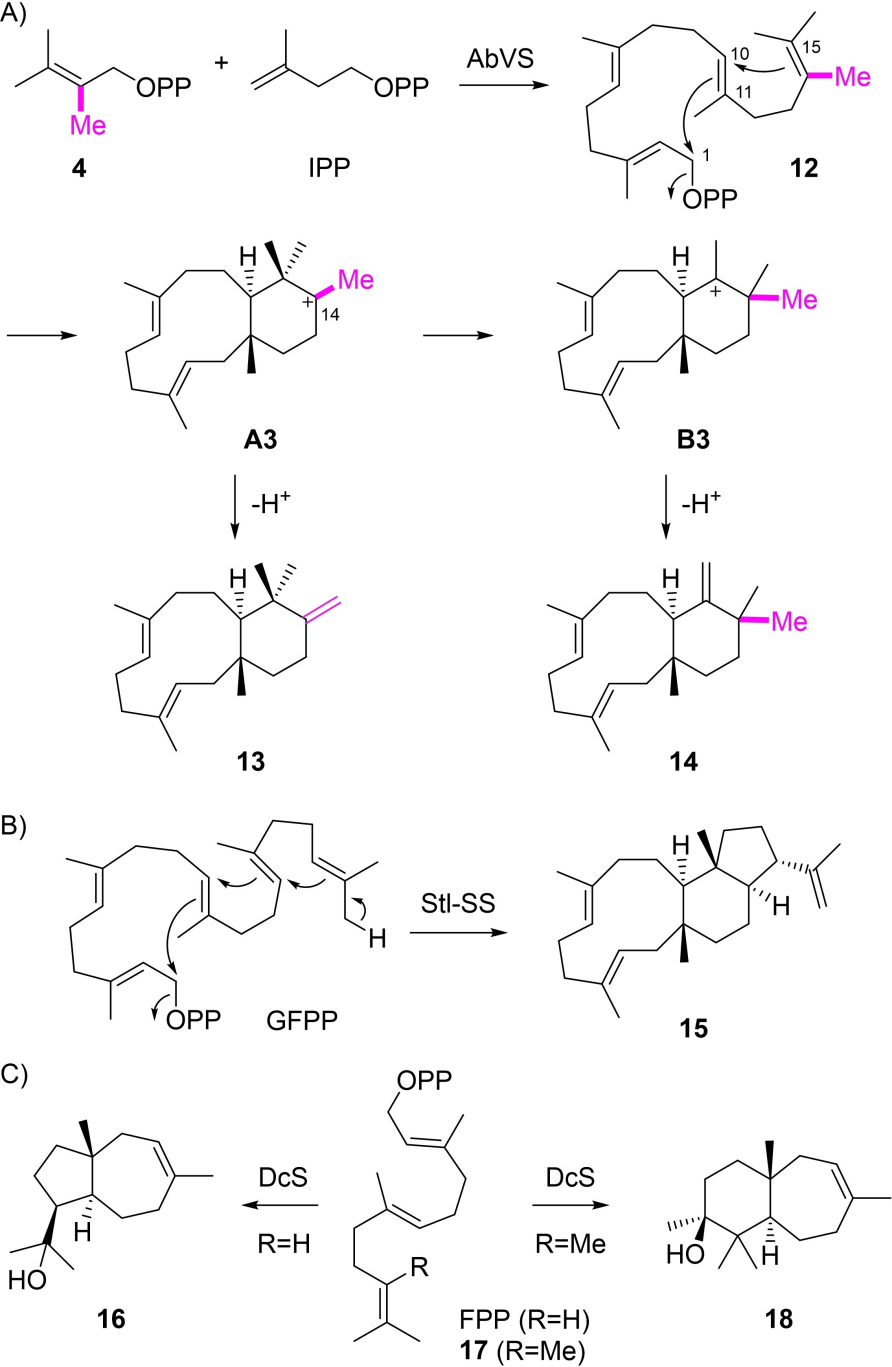
A) Enzymatic conversion of **4** and IPP with AbVS into **13** and **14**. B) Conversion of GFPP into stellatatriene (**15**) by Stl‐SS. C) Conversion of FPP into dauc‐8‐en‐11‐ol (**16**) and of 10‐methyl‐FPP (**17**) into **18** by DcS.

The incubation of **4** and IPP with AbVS yielded two compounds that were isolated and structurally characterised by NMR as **13** and **14** (Tables S3 and S4, Figures S17–S32). The substrate analog **4** also opens a new reaction path through 1,11–10,15 cyclisation to **A3**, followed by deprotonation to isohomodolabellatriene I (**13**), or a 1,2‐methyl shift to **B3** and loss of a proton to isohomodolabellatriene II (**14**, Scheme 3A). In the intermediate **A3** the tertiary cation at C14 is stabilised by the additional Me group, allowing for closure of a 6‐membered ring, while for the native substrate GGPP this reaction would lead to a secondary cation which is thus not preferred. For the biosynthesis of stellatatriene (**15**) by Stl‐SS from *Emericella variecolor* a similar cyclisation reaction is observed,^[32]^ but for this sesterterpene the secondary cation potentially arising by the closure of the six‐membered ring may be a highly transient species that is directly captured by a third cyclisation event (Scheme 3B). A similar change in reactivity from the naturally observed five‐membered ring in dauc‐8‐en‐11‐ol (**16**) obtained from farnesyl diphosphate (FPP) to formation of the six‐membered ring compound **18** from 10‐methyl‐FPP (**17**) was recently described for dauc‐8‐en‐11‐ol synthase (DcS) from *Streptomyces venezuelae* (Scheme 3C).^[19]^

**Scheme 4:**
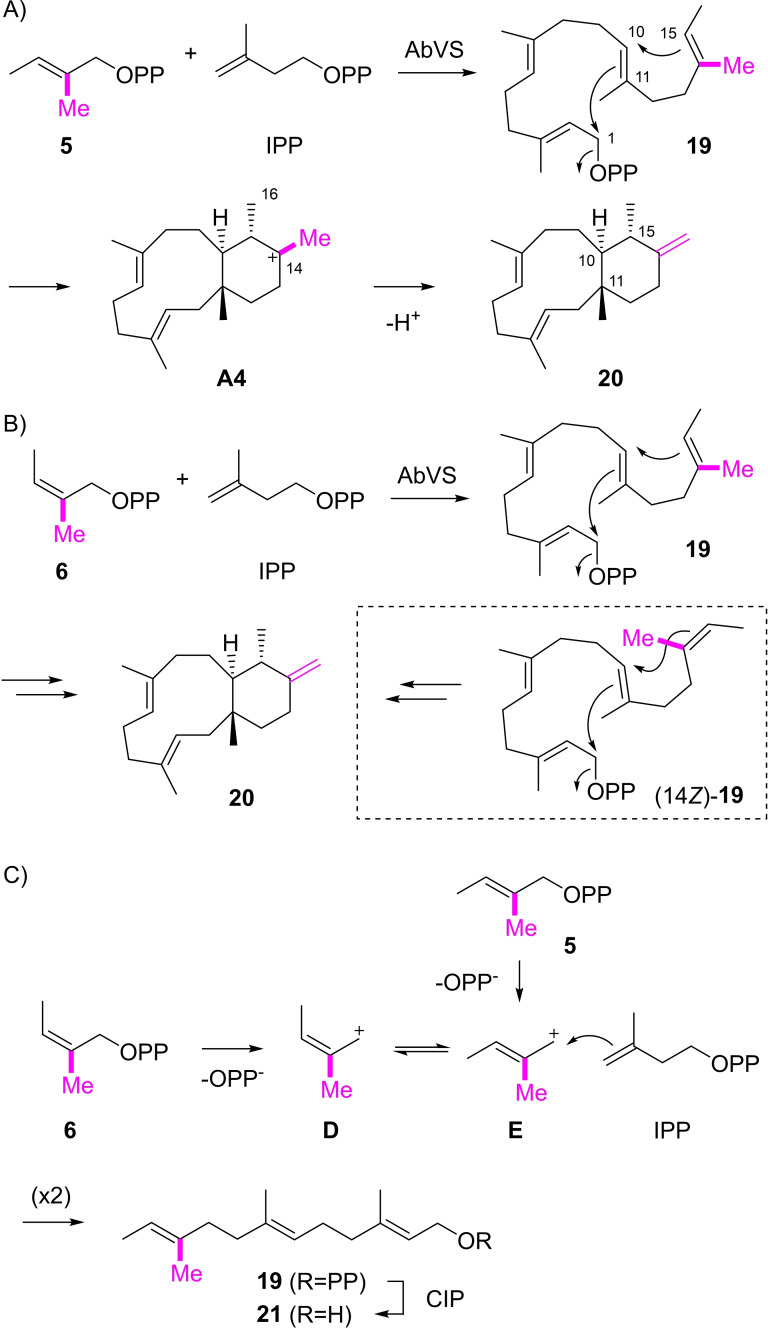
A) Enzymatic conversion of **5** and IPP with AbVS into **20**. B) Conversion of **6** and IPP yields the same compound **20**, presumably via the same intermediate **19** with double bond isomerisation in the portion derived from **6**. The dashed box shows the alternative intermediate (14*Z*)‐**19** formed from **6** without double bond isomerisation and its cyclisation to **20** through a different conformation as for **19** in part A. C) Double bond isomerisation in **6** during elongation with IPP.

The enzymatic reaction of DMAPP analog **5** with a shifted Me group and IPP catalysed by AbVS revealed a similar reactivity as observed with substrate **4**, resulting in the formation of a six‐membered ring in the product isodolabellatriene (**20**) through 1,11‐10,15‐cyclisation (Scheme 4A, for NMR data cf. Table S5 and Figures S33–S40). Also in this case the changed cyclisation mode in comparison to GGPP cyclisation can be explained by the stabilisation of the tertiary cation at C14 in intermediate **A4** by the additional Me group. Its deprotonation then leads to **20**. Surprisingly, the conversion of the *Z* stereoisomer **6** resulted in the formation of the same material (Scheme 4B). In addition, the incubation of both DMAPP analogs **5** and **6** with IPP and FPP synthase (FPPS) from *Streptomyces coelicolor* A3(2)^[33]^ and dephosphorylation with calf intestinal phosphatase (CIP) resulted in both cases in an alcohol of same retention time and mass spectrum, tentatively identified as **21** by GC/MS (Scheme 4C, Figure S41), suggesting that the DMAPP analog **6** undergoes a double bond isomerisation during the elongation with IPP. This isomerisation is explainable, because this elongation reaction proceeds with enzyme catalysed substrate ionisation of **6** through diphosphate abstraction, yielding an allyl cation intermediate **D** in which the double bond character is significantly reduced, with the consequence of a lowered configurational stability that allows isomerisation to **E**. Whether these cationic species exist freely or only transiently remains an open question, but even if they exist only as transient species, this may explain the observed configurational instability of **6**. The alternative configurational isomerisation of **5** would be difficult to understand, and compound **20** can be regarded as the expected product from a 1,11‐10,15‐cyclisation from **19**: C1 of **19** is at the bottom and is attacked from top by C11. The attack at C10 must be *anti* to the attack at C11, so that C15 will be one level higher, exposing its *Re* face to C10, which explains all configurations at C10, C11 and C15 in **20**. An alternative explanation would be as follows: Diphosphate **5** may result in the 14*E* and **6** in the 14*Z* stereoisomer of **19** and both of these stereoisomers may react to the same compound **20** through different conformations (this alternative is shown in the dashed box of Scheme 4B). However, as it seems that the same alcohol **21** was obtained in the dephosphorylation with CIP (Scheme 4C, Figure S41), the hypothetical isomerisation of **6** during elongation to **19** may give the better explanation.

The absolute configurations of all obtained compounds **8**, **10**, **13**, **14** and **20** were determined through an enantioselective deuteration strategy developed in our laboratory.^[34]^ This method makes use of the stereoselectively deuterated substrates (*R*)‐ and (*S*)‐(1‐^13^C,1‐^2^H)IPP^[35]^ and (*E*)‐ and (*Z*)‐(4‐^13^C,4‐^2^H)IPP.^[36]^ Based on Cornforth's fundamental work regarding the stereochemical course of FPPS and related oligoprenyl diphosphate synthases,^[37]^ from these substrates artifical stereogenic centers of known configuration can be introduced into the acyclic terpene precursors. Their conversion by TPSs leads to the introduction of these stereochemical anchors into terpene hydrocarbons, and the determination of the relative orientation of the naturally present stereogenic centers to these anchors then allows to conclude on the absolute configuration of the terpenes. In practice, the relative orientations of diastereotopic hydrogens in methylene groups are first assigned for the unlabelled compounds by NOESY. After conversion of the labelled stereoselectively deuterated precursors the incorporation into the diastereotopic hydrogen positions can be followed with high sensitivity through HSQC spectroscopy, for which the additional ^13^C‐substitutions were introduced. All combinations of the DMAPP analogs **2**–**6** with the four labelled substrates (*R*)‐ and (*S*)‐(1‐^13^C,1‐^2^H)IPP and (*E*)‐ and (*Z*)‐(4‐^13^C,4‐^2^H)IPP were converted with AbVS, with addition of FPPS and GGPP synthase (GGPPS) from *Streptomyces cyaneofuscatus* ^[34]^ that enhanced turnover, followed by HSQC analysis of the products (Figures S42–S51), revealing the absolute configurations of compounds **8**, **10**, **13**, **14** and **20** as shown in Schemes 2–4. Notably, these absolute configurations correspond to those of **1** and the intermediates along its cyclisation cascade (Scheme 1).

## Conclusion

The bifunctional diterpene synthase AbVS naturally converts DMAPP and IPP into the diterpene hydrocarbon variediene (**1**). Herein, we show for the first time that both domains of a fungal bifunctional terpene synthase can collaborate in the enzymatic conversion of analogs of the terpene monomer DMAPP that is elongated by the PT domain with IPP to obtain the corresponding GGPP analogs. Further conversion by the TC domain leads to analogs of diterpene hydrocarbons. Depending on the methylation pattern of the DMAPP analogs, in several cases new reaction paths that are not observed with the natural substrate GGPP were opened, because charges in the cationic intermediates can be stabilised at alternative carbon atoms. This approach gives enzymatic access to compounds that are naturally not obtained. While the cyclisation mechanism can change according to the reactivity implemented in the DMAPP analogs, the absolute configurations of the obtained compounds that are determined during the first cyclisation step, with all subsequent transformations being diastereoselective, are analogous to that of the natural enzyme product **1**. Besides the varidiene synthase[^26^, ^27^] several other bifunctional fungal di‐ and sesterterpene synthases have been discovered recently,[^38^, ^39^, ^40^, ^41^, ^42^, ^43^, ^44^, ^45^, ^46^, ^47^, ^48^, ^49^, ^50^, ^51^, ^52^, ^53^] which offer an interesting playground for future research adopting a similar strategy of using non‐natural DMAPP and IPP analogs for the enzymatic enantioselective synthesis of structurally complex terpene analogs with potentially novel skeletons.

## Conflict of interest

The authors declare no conflict of interest.

1

## Supporting information

As a service to our authors and readers, this journal provides supporting information supplied by the authors. Such materials are peer reviewed and may be re‐organized for online delivery, but are not copy‐edited or typeset. Technical support issues arising from supporting information (other than missing files) should be addressed to the authors.

Supporting Information

## Data Availability

The data that support the findings of this study are available in the supplementary material of this article.
