# Supplementary material for: Enzymatic Synthesis of Variediene Analogs
Source: Chemistry. 2022 Feb 9;28(15):e202200095. doi: 10.1002/chem.202200095 (PMC9305479; doi:10.1002/chem.202200095)
Supplement: Supplementary file 1 — Supporting Information [file CHEM-28-0-s001.pdf]

Chemistry–A European Journal

Supporting Information

Enzymatic Synthesis of Variediene Analogs

Lin-Fu Liang and Jeroen S. Dickschat*

NMR and IR spectroscopic analysis
NMR spectra of isolated natural products were recorded on a Bruker AV Avance DMX-500 (500 MHz), a Bruker AV III HD Cryo (700 MHz) and a Bruker AV III HD Prodegy (500 MHz) spectrometer, and were referenced against solvent signals (H-NMR, residual proton signals: C_6D_6 δ = 7.16, D_2O δ = 4.79; ^{13}C-NMR: C_6D_6 δ = 128.06 ppm). IR spectra of the isolated natural products were recorded on a Bruker alpha FTIR spectrometer.

GC–MS analysis
GC–MS analyses were carried out with a 7890B gas chromatograph connected to a 5977A inert mass detector (Agilent) fitted with a HP5-MS fused silica capillary column (30 m, 0.25 mm i. d., 0.50 μm film). Instrumental parameters were (1) inlet pressure, 77.1 kPa, He 23.3 mL min^{-1}, (2) injection volume, 1 μL, (3) transfer line, 250 °C, and (4) electron energy 70 eV. The GC was programmed as follows: 5 min at 50 °C increasing at 10 °C min^{-1} to 320 °C, and operated in split mode (splitless, 60 s valve time). The carrier gas was He at 1 mL min^{-1}. Retention indices (*I*) were determined from a homologous series of *n*-alkanes (C_7−C_{40}). Compound identification of the side products of terpene cyclases was based on a matching mass spectrum to a library mass spectrum and retention index to published data.

APCI–MS measurements
High resolution mass spectra using APCI were recorded on an Orbitrap XL instrument (Thermo Fisher Scientific, Waltham, MA, USA).

Gene expression and protein purification
A preculture of *E. coli* BL21(DE3) transformed with pYE-OJJ72250[1] was grown in LB medium with kanamycin (50 μg mL^{-1}) overnight while shaking at 37 °C. A gene expression culture inoculated with the preculture (1/1000) was grown in LB medium with kanamycin (50 μg mL^{-1}) shaking at 37 °C until OD_{600} = 0.4-0.6 was reached. After cooling to 18 °C enzyme expression was induced by addition of aqueous IPTG solution (400 mM, 1/1000). The culture was shaken at 18 °C overnight and cells were harvested via centrifugation (9800 g, 5 min, 4 °C). The cells were lysed by ultra-sonication (3x 1 min) on ice, after resuspending the cell pellet with binding buffer (10 mL L^{-1}; 20 mM Na_2HPO_4, 0.5 M NaCl, 20 mM imidazole, 1 mM $MgCl_2$, pH 7.4). The cell debris were removed by centrifugation (14600 g, 7 min, 4 °C) and the soluble protein fractions were loaded to Ni^{2+}-NTA superflow affinity chromatography columns by Qiagen (Venlo, Netherlands) equilibrated with binding buffer. The columns were washed with binding buffer (2x 10 mL L^{-1} culture) and the desired proteins were eluted with elution buffer (2x 10 mL $^{-1}$ culture; 20 mM Na_2HPO_4, 500 mM NaCl, 500 mM imidazole, 1 mM $MgCl_2$, pH 7.4). The obtained fraction was directly used for incubation experiments.

Enzyme reaction and product isolation
Test incubations to identify the substrate scope of recombinant AbVS were performed with DMAPP analogues ((*E*)-3-methylpent-2-enyl diphosphate (**2**), (*Z*)-3-methylpent-2-enyl diphosphate (**3**), 2,3-dimethylbut-2-enyl diphosphate (**4**), (*E*)-2-methylbut-2-enyl diphosphate (**5**) and (*Z*)-2-methylbut-2-enyl diphosphate (**6**)) or DMAPP and IPP (1 mg) dissolved in substrate buffer (100 μL; 25 mM NH_4HCO_3) and diluted with incubation buffer (600 μL; 50 mM Tris/HCl, 10 mM $MgCl_2$, 20% glycerol, pH 8.2). Protein preparations (AbVS and GGPPS, each 150 μL) obtained from 500 mL expression culture were added respectively, followed by incubation with shaking at 28 °C for 4 h. The products were extracted with hexane (100 μL), the extracts were dried with $MgSO_4$ and analysed by GC/MS. These incubations showed the production of variediene analogues.
For preparative isolation of variediene analogues, large scale incubations were done by dissolving DMAPP analogues (50 mg) and IPP (150 mg) in substrate buffer (15 mL). These incubation experiments were performed with a syringe pump, which added the substrate solution over 1 h to the enzyme elution fractions (AbVS 10 mL, from 4 L expression culture; GGPPS 5 mL, from 2 L expression culture) diluted by incubation buffer 70 mL. The reaction

mixtures were incubated at 28 °C overnight and then extracted with pentane (3x 100 mL), the extracts were dried with $MgSO_4$ and concentrated in vacuo. Column chromatography on silica gel with pentane yielded the pure diterpenes **8**, **10**, **13**, **14** and **20** as a colorless oil.

Synthetic routes to (*E*)-3-methylpent-2-enyl diphosphate (2), (*Z*)-3-methylpent-2-enyl diphosphate (3) and (*Z*)-2-methylbut-2-enyl diphosphate (6)

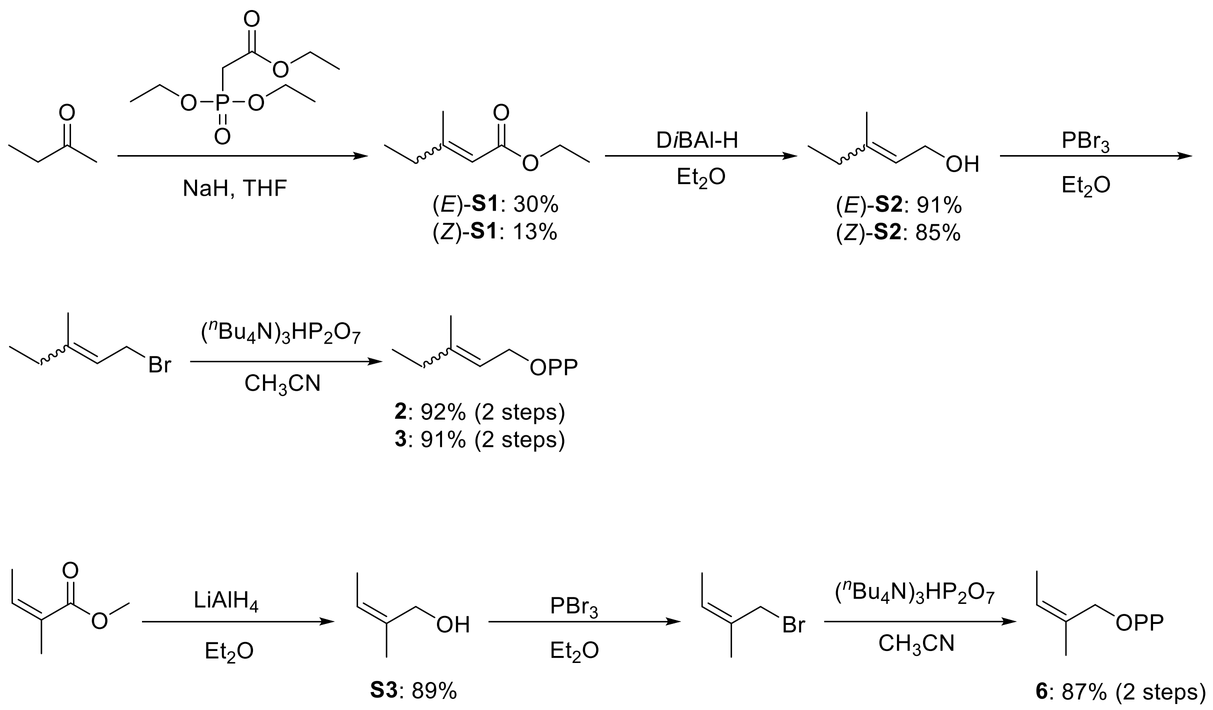

Scheme S1. Synthesis of **2**, **3** and **6**.

Synthesis of ethyl 3-methylpent-2-enoate ((*Z*/*E*)-S1)

To a solution of triethylphosphonoacetate (19.0 mL, 95.8 mmol, 2.4 eq.) and NaH (2.30 g, 95.8 mmol, 2.4 eq.) in THF (100 mL) was added 2-butanone (3.58 mL, 40.0 mmol, 1.0 eq.). The mixture was stirred at 0 °C for 12 h, then quenched with an aqueous ammonium chloride solution (100 mL) and extracted with ethyl acetate (3 × 100 mL). The combined organic layers were washed with brine, dried over anhydrous $MgSO_4$, and concentrated under reduced pressure to give the crude product. Purification by silica gel column chromatography using pentane/diethyl ether (20:1) as an eluent afforded the products (*Z*)-**S1** (0.74 g, 5.18 mmol, 13%) and (*E*)-**S1** (1.70 g, 12.0 mmol, 30%). (*Z*)-**S1**: ^{1}H-NMR ($CDCl_3$, 300 MHz): δ = 5.63 (s, 1H) , 4.14 (q, $^3J_{H,H}$ = 7.1 Hz, 2H), 2.63 (q, $^3J_{H,H}$ = 7.6 Hz, 2H), 1.88 (d, $^4J_{H,H}$ = 1.4 Hz, 3H), 1.27 (t, $^3J_{H,H}$ = 7.1 Hz, 3H), 1.07 (t, $^3J_{H,H}$ = 7.6 Hz, 3H) ppm. (*E*)-**S1**: ^{1}H-NMR ($CDCl_3$, 300 MHz): δ = 5.66 (s, 1H) , 4.15 (q, $^3J_{H,H}$ = 7.1 Hz, 2H), 2.16 (q, $^3J_{H,H}$ = 7.5 Hz, 2H), 2.16 (s, 3H), 1.28 (t, $^3J_{H,H}$ = 7.1 Hz, 3H), 1.07 (t, $^3J_{H,H}$ = 7.5 Hz, 3H) ppm. NMR data were consistent with those reported in the literature.[2,3]

Synthesis of 3-methylpent-2-en-1-ol ((*Z*/*E*)-S2)

To a cooled (–30 °C) solution of (*Z*)-**S1** (0.36 g, 2.5 mmol, 1.0 eq) in diethyl ether (50 mL) was slowly added DiBAl-H (1 M in hexane, 1.0 mL, 5.5 mmol, 2.2 eq). The mixture was stirred overnight with slow warming to room temperature. The reaction was cooled to 0 °C and saturated aqueous Na-K-tartrate solution was added. The aqueous phase was extracted three times with diethyl ether and the combined organic phases were dried with $MgSO_4$, carefully

concentrated under reduced pressure (350 mbar) and purified by column chromatography (pentane/diethyl ether, 1:1) to yield the product (*Z*)-**S2** (0.21 g, 2.1 mmol, 85%) as a colourless oil. Following the same procedure, (*E*)-**S1** (1.24 g, 8.8 mmol) was converted into (*E*)-**S2** (0.80 g, 8.0 mmol, 91%) that was obtained as a colourless oil. (*Z*)-**S2**: ^{1}H-NMR ($CDCl_3$, 500 MHz): δ = 5.38 (t, $^3J_{H,H}$ = 7.2 Hz, 1H), 4.13 (d, $^3J_{H,H}$ = 7.1 Hz, 2H), 2.09 (q, $^3J_{H,H}$ = 7.6 Hz, 2H), 1.74 (s, 3H), 0.99 (t, $^3J_{H,H}$ = 7.6 Hz, 3H) ppm. ^{13}C-NMR ($CDCl_3$, 125 MHz): δ = 142.3 (Cq), 123.4 (CH), 59.2 (CH_2), 25.1 (CH_3), 23.1 (CH_2), 13.3 (CH_3) ppm. (*E*)-**S2**: ^{1}H-NMR ($CDCl_3$, 500 MHz): δ = 5.40 (tq, $^3J_{H,H}$ = 7.0 Hz, $^4J_{H,H}$ = 1.4 Hz, 1H), 4.16 (d, $^3J_{H,H}$ = 7.0 Hz, 2H), 2.03 (q, $^3J_{H,H}$ = 7.5 Hz, 2H), 1.68 (s, 3H), 1.02 (t, $^3J_{H,H}$ = 7.5 Hz, 3H) ppm. ^{13}C-NMR ($CDCl_3$, 125 MHz): δ = 141.8 (Cq), 122.1 (CH), 59.6 (CH_2), 32.3 (CH_2), 16.3 (CH_3), 12.5 (CH_3) ppm. NMR data were consistent with those reported in the literature.[4,5]

Synthesis of 3-methylpent-2-enyl diphosphate (2 and 3)

A solution of (*E*)-**S2** (0.80 g, 8.0 mmol, 1.0 eq) in diethyl ether (25 mL) was cooled to 0 °C and phosphorous tribromide (0.32 mL, 1.3 mmol, 0.4 eq) was slowly added. After 2 h ice cold saturated K_2CO_3 solution was added. The aqueous layer was extracted quickly with diethyl ether three times. The combined organic phases were dried with $MgSO_4$, concentrated carefully under reduced pressure and directly added to a solution of $(NBu_4)_3HP_2O_7$ (14.25 g, 8.0 mmol, 2.0 eq) in acetonitrile (4 mL). The mixture was stirred overnight and was concentrated under reduced pressure. The residue was dissolved in aqueous ammonium bicarbonate solution (25 mM) and loaded onto a DOWEX® 50WX8 cation exchange column (NH_4^+ form, pH ~ 7.0). The desired compound was eluted with 1.5 column volumes of NH_4HCO_3 buffer (25 mM, 5% *i*PrOH), the eluate was frozen with liquid N_2 and lyophilized to yield the diphosphate **2** as a white powder (2.30 g, 7.4 mmol, 92%). Following the same procedure, (*Z*)-**S2** (0.21 g, 2.1 mmol) was converted into **3** (0.60 g, 1.9 mmol, 91%) that was obtained as a colourless oil. **2**: ^{1}H-NMR (D_2O, 500 MHz): δ = 5.36 (t, $^3J_{H,H}$ = 7.3 Hz, 1H), 4.38 (t, $^3J_{H,H}$ = 6.8 Hz, 2H), 1.98 (q, $^3J_{H,H}$ = 7.5 Hz, 2H), 1.63 (s, 3H), 0.92 (t, $^3J_{H,H}$ = 7.5 Hz, 3H) ppm. ^{13}C-NMR (D_2O, 126 MHz): δ = 145.0 (Cq), 118.1 (d, $^3J_{C,P}$ = 8.5 Hz, CH), 62.5 (d, $^2J_{C,P}$ = 5.2 Hz, CH_2), 31.6 (CH_2),15.6 (CH_3), 11.6 (CH_3) ppm. ^{31}P-NMR (D_2O, 202 MHz): δ = –6.6 (d, $^2J_{P,P}$ = 21.4 Hz), –10.2 (d, $^2J_{P,P}$ = 21.4 Hz) ppm. **3**: ^{1}H-NMR (D_2O, 500 MHz): δ = 5.38 (t, $^3J_{H,H}$ = 6.5 Hz, 1H), 4.41 (t, $^3J_{H,H}$ = 6.8 Hz, 2H), 2.09 (q, $^3J_{H,H}$ = 7.6 Hz, 2H), 1.72 (s, 3H), 0.94 (t, $^3J_{H,H}$ = 7.6 Hz, 3H) ppm. ^{13}C-NMR (D_2O, 126 MHz): δ = 145.5 (Cq), 119.3 (d, $^3J_{C,P}$ = 8.4 Hz, CH), 62.1 (d, $^2J_{C,P}$ = 5.2 Hz, CH_2), 24.5 (CH_2), 22.1 (CH_2), 12.4 (CH_3) ppm. ^{31}P-NMR (D_2O, 202 MHz): δ = –6.4 (d, $^2J_{P,P}$ = 21.9 Hz), –10.3 (d, $^2J_{P,P}$ = 21.9 Hz) ppm. NMR spectra of **2** and **3** are shown in Figures S52 – S57.

Synthesis of (*Z*)-2-methyl-2-buten-1-ol (S3)

$LiAlH_4$ (1.11 g, 29.3 mmol, 3.0 eq) was added into a 100 mL flame-dried round bottom flask equipped with a stir bar. Et_2O (16 mL) was added and the mixture was cooled to 0 °C. Methyl angelate (1.12 g, 9.8 mmol) was added dropwise at this temperature. Then the mixture was stirred at room temperature for 2 h. After cooling to 0 °C the reaction was quenched by slow addition of sat. Na_2SO_4 until no white solid formed. Sat. Rochelle's salt (25 mL) was added and the aqueous layer was extracted using Et_2O (3 x 40 mL). The combined organic layers were washed with brine (3 x 100 mL), dried over $MgSO_4$, filtered and carefully concentrated under reduced pressure (350 mbar) to afford the crude product, which was purified by column chromatography (pentane/diethyl ether, 2:1) to yield the product **S3** as colorless liquid (0.75 g, 8.7 mmol, 89%). TLC (pentane/diethyl ether, 1:1): R_f = 0.47, ^{1}H-NMR (C_6D_6, 700 MHz): δ = 5.19 (m, 1H), 3.93 (br s, 2H), 1.74 (m, 3H), 1.46 (d, $^3J_{H,H}$ = 7.1 Hz, 3H). 1.21 (br s, 1H) ppm. ^{13}C-NMR (C_6D_6, 176 MHz): δ = 135.9 (Cq), 121.5 (CH), 61.0 (CH_2), 21.3 (CH_3), 13.1 (CH_3) ppm. NMR spectra of **S3** are shown in Figures S58 – S64.

Synthesis of (*Z*)-2-methyl-2-butenyl diphosphate (6)

A solution of **S3** (0.29 g, 3.4 mmol, 1.0 eq) in diethyl ether (4 mL) was cooled to 0 °C and phosphorous tribromide (0.13 mL, 1.3 mmol, 0.4 eq) was slowly added. After 2 h ice cold saturated K_2CO_3 solution was added. The aqueous layer was extracted quickly with diethyl ether three times. The combined organic phases were dried with $MgSO_4$, concentrated carefully under reduced pressure and directly added to a solution of $(NBu_4)_3HP_2O_7$ (6.11 g, 6.77 mmol, 2.0 eq) in acetonitrile (4 mL). the mixture was stirred overnight and was concentrated under reduced pressure. The residue was dissolved in aqueous ammonium bicarbonate solution (25 mM) and loaded onto a DOWEX® 50WX8 cation exchange column (NH_4^+ form, pH ~ 7.0). The desired compound was eluted with 1.5 column volumes of NH_4HCO_3 buffer (25 mM, 5% *i*PrOH), the eluate was frozen with liquid N_2 and lyophilized to yield the diphosphate as a white powder (0.88 g, 2.95 mmol, 87%). ^{1}H-NMR (D_2O, 700 MHz): δ = 5.64 (q, $^3J_{H,H}$ = 7.0 Hz, 1H), 4.49 (d, $^3J_{H,H}$ = 5.9 Hz, 2H), 1.78 (br s, 3H), 1.67 (d, $^3J_{H,H}$ = 6.9 Hz, 3H) ppm. ^{13}C-NMR (D_2O, 176 MHz): δ = 132.3 (d, $^3J_{C,P}$ = 8.1 Hz, Cq), 124.6 (CH), 64.2 (d, $^2J_{C,P}$ = 5.5 Hz, CH_2), 20.5 (d, $^4J_{C,P}$ = 1.5 Hz, CH_3), 12.6 (d, $^6J_{C,P}$ = 2.4 Hz, CH_3) ppm. ^{31}P-NMR (D_2O, 202 MHz): δ = –7.1 (br s), –10.2 (br s) ppm. NMR spectra of **6** are shown in Figures S65 – S67.

Homovariediene (8). Yield: 0.9 mg (3.1 μmol, 1.9%), from 50 mg (160.7 μmol) **2** trisammonium salt. TLC (pentane): R_f = 0.85. IR (diamond ATR): ṽ = 2961 (w), 2926 (w), 2855 (w), 1736 (w), 1716 (w), 1452 (w), 1412 (w), 1376 (w), 1260 (m), 1087 (m), 1021 (m), 864 (w), 796 (m), 702 (w), 662 (w) cm^{-1}. HR-MS (APCI): calc. for $[C_{21}H_{35}]^+$ m/z = 287.2733; found: m/z = 287.2744. Optical rotary power: $[\alpha]_D^{25}$ = −8.9 (c 0.09, C_6H_6).

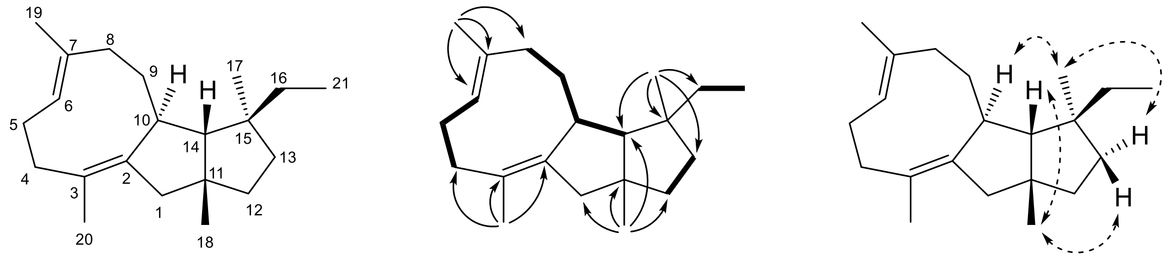

8

Figure S1. Structure elucidation of **8**. Bold: ^{1}H,^{1}H-COSY, single headed arrows: key HMBC, and dashed double headed arrows: key NOESY correlations. Carbon numbering follows GGPP numbering to indicate the origin of each carbon.

Table S1. NMR data of homovariediene (**8**) in C_6D_6 recorded at 298 K.

C[a]	type	^{1}H[b]	^{13}C[b]
1	CH_2	2.18 (d, $^{2}J_{H,H}$ = 14.6, H_α)<br>2.05 (d, $^{2}J_{H,H}$ = 14.5, H_β)	44.27
2	C_q	–	144.03
3	C_q	–	121.81
4	CH_2	1.94 (m, H_α)<br>1.90 (m, H_β)	38.19
5	CH_2	2.01 (m, H_β)<br>1.89 (m, H_α)	25.26
6	CH	5.25 (dd, $^{3}J_{H,H}$ = 11.3, 3.5)	127.36
7	C_q	–	138.41
8	CH_2	2.15 (m, H_α)<br>2.08 (m, H_β)	41.03
9	CH_2	2.16 (m, H_β)<br>1.56 (m, H_α)	41.39
10	CH	2.29 (d, $^{3}J_{H,H}$ = 10.6)	44.82
11	C_q	–	48.47
12	CH_2	1.43 (m, 2H)	40.81
13	CH_2	1.39 (m, H_α)<br>1.29 (m, H_β)	40.05
14	CH	1.32 (m)	67.43
15	C_q	–	46.90
16	CH_2	1.32 (m, 2H)	37.89
17	CH_3	0.81 (s)	20.17
18	CH_3	1.20 (s)	31.17
19	CH_3	1.40 (d, $^{4}J_{H,H}$ = 0.8)	16.34
20	CH_3	1.57 (d, $^{4}J_{H,H}$ = 1.4)	21.54
21	CH_3	0.88 (t, $^{3}J_{H,H}$ = 7.5)	9.75

[a] Carbon numbering as shown in Figure S1. [b] Chemical shifts δ in ppm, multiplicity: s = singlet, d = doublet, t = triplet, m = multiplet, coupling constants *J* are given in Hertz.

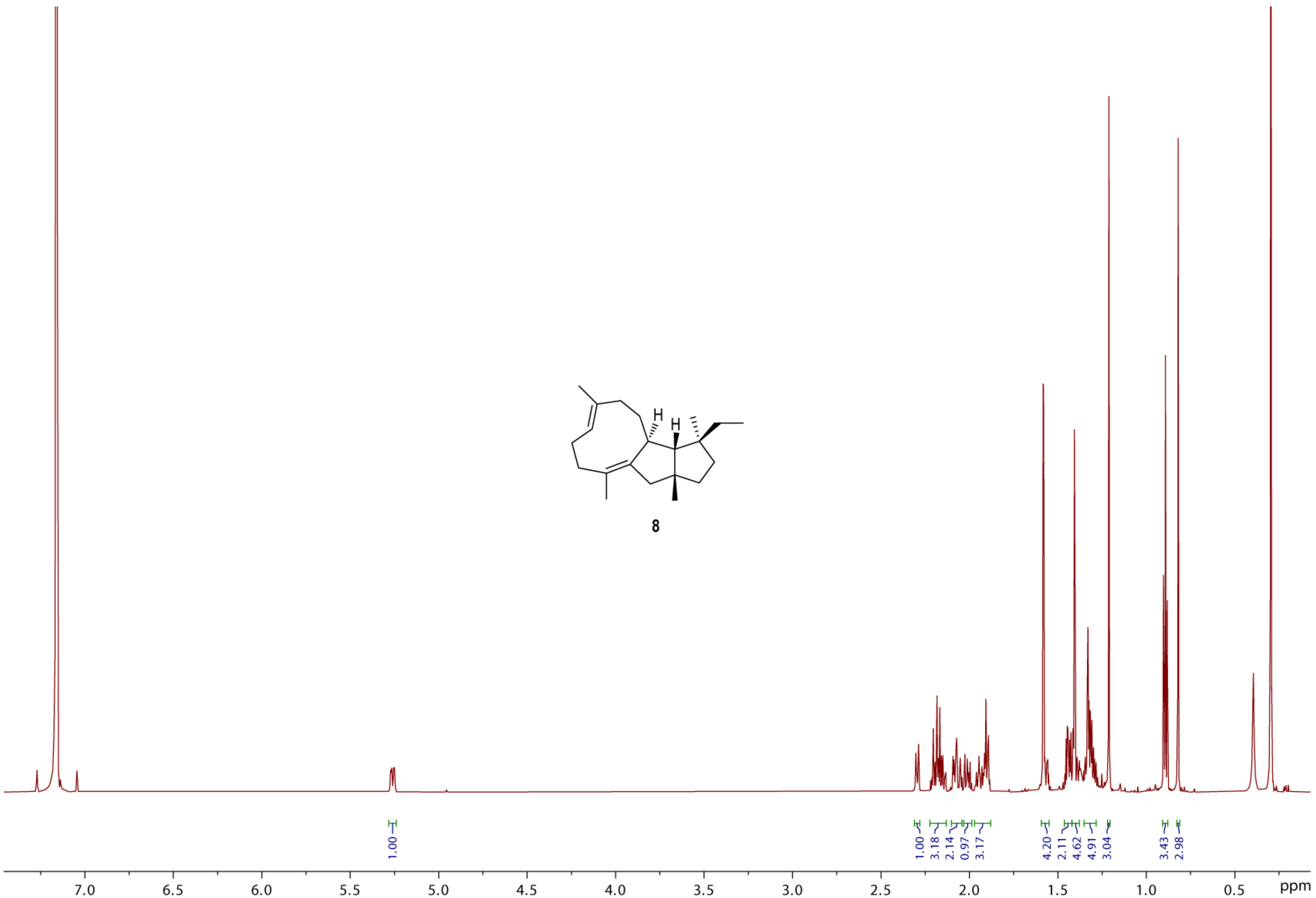

Figure S2. ^{1}H-NMR spectrum of **8** (700 MHz, C_6D_6).

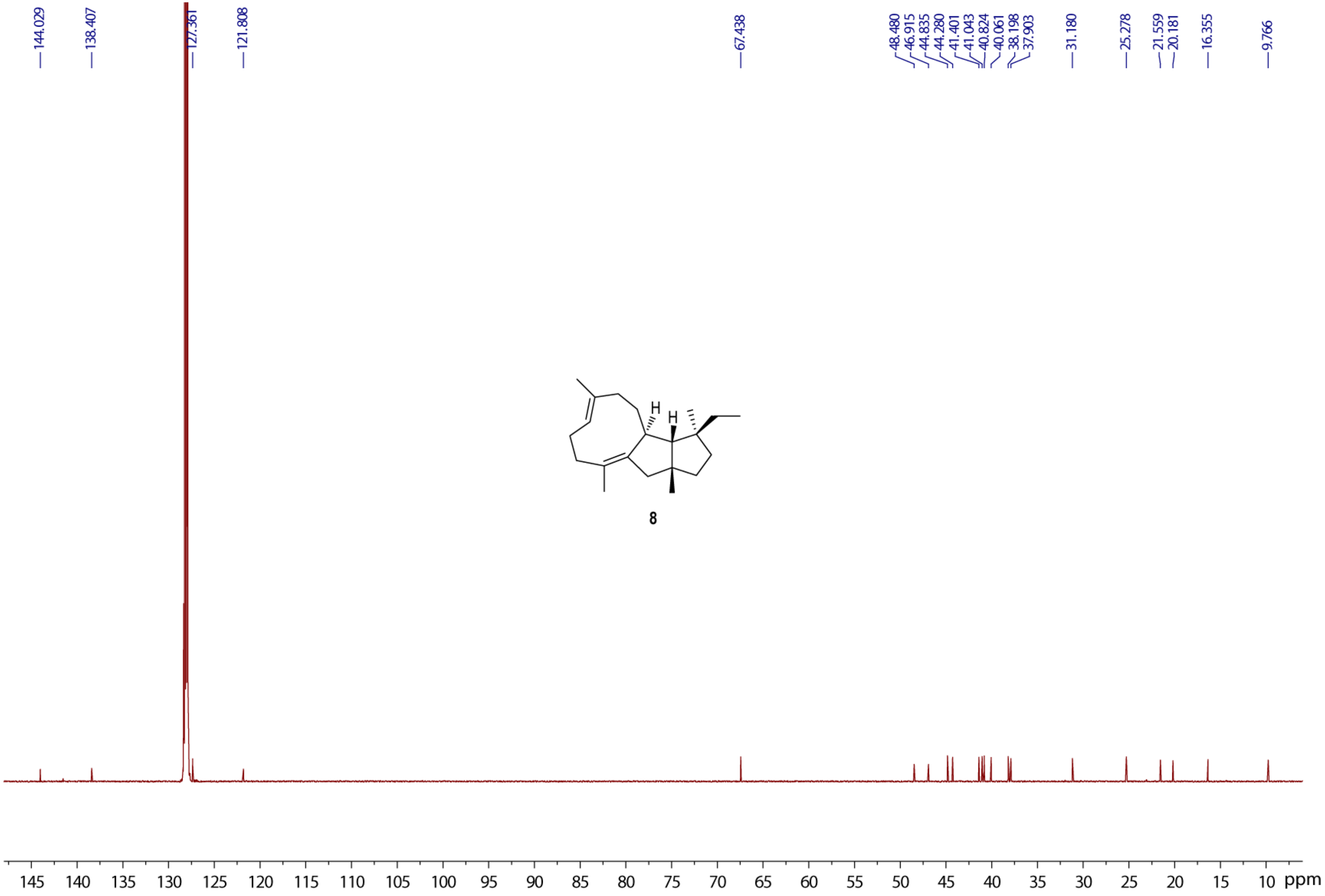

Figure S3. ^{13}C-NMR spectrum of **8** (176 MHz, C_6D_6).

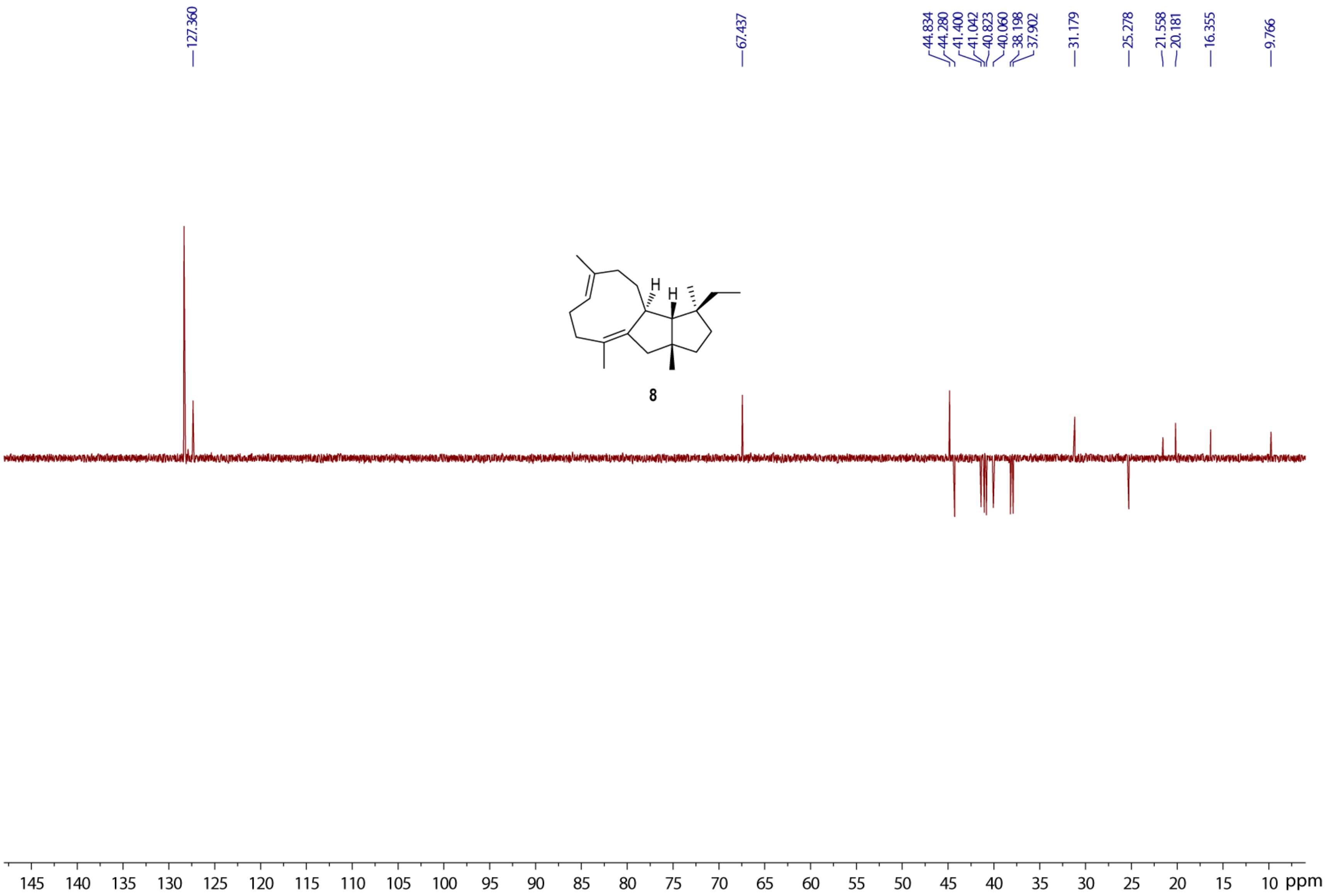

Figure S4. ^{13}C-DEPT spectrum of **8** (176 MHz, C_6D_6).

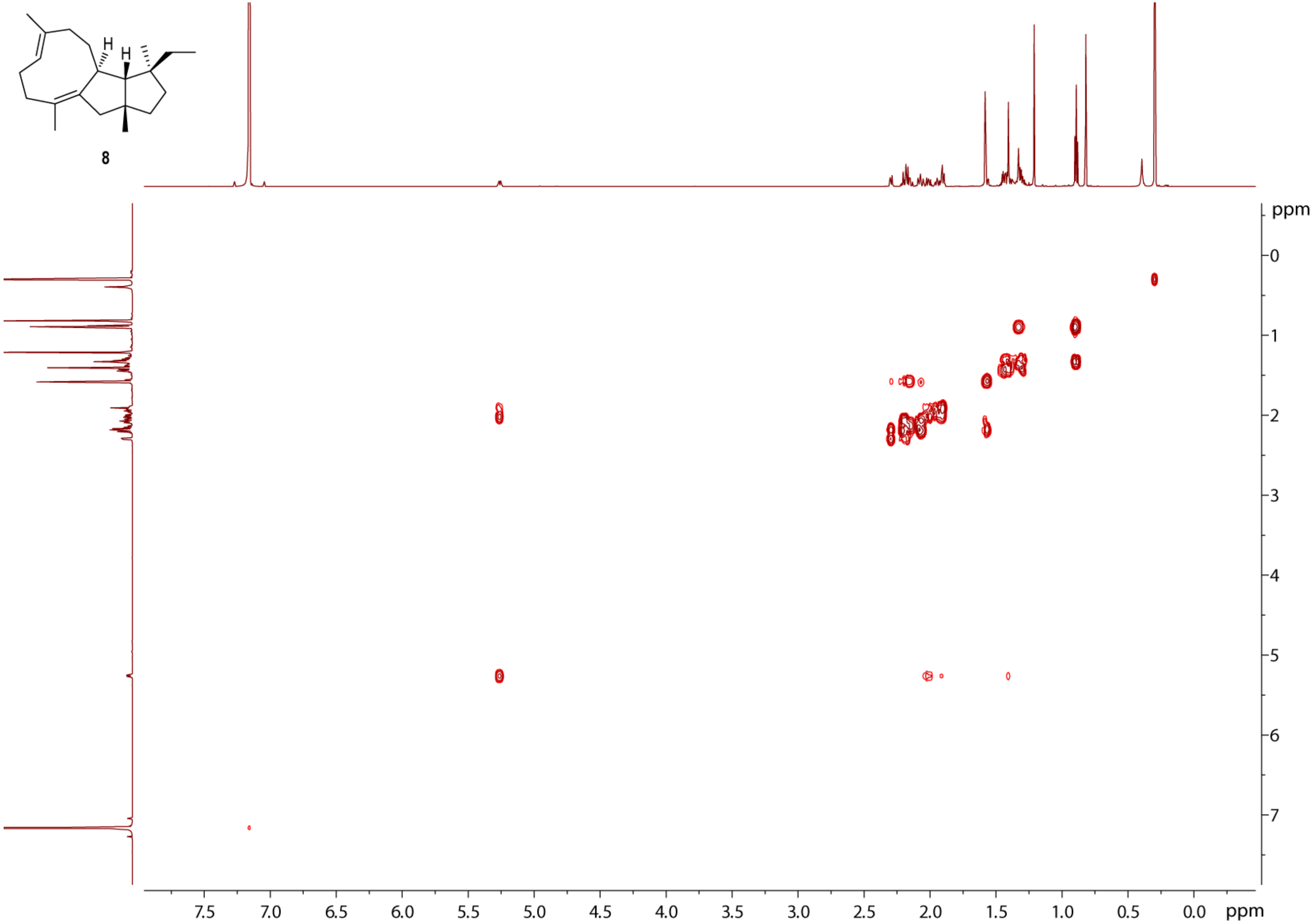

Figure S5. ^{1}H-^{1}H-COSY spectrum of **8** (700 MHz, C_6D_6).

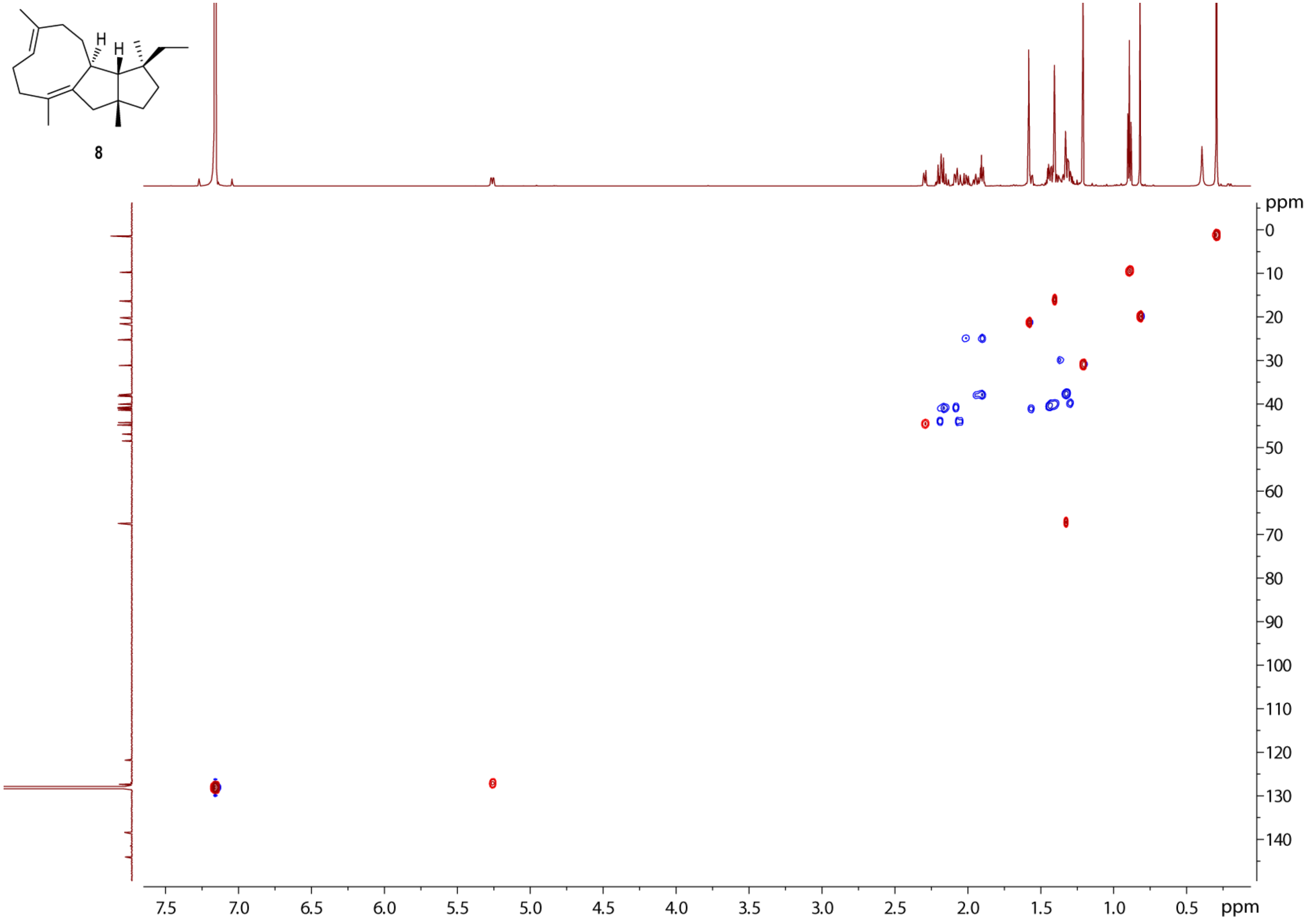

Figure S6. HSQC spectrum of **8** (C_6D_6).

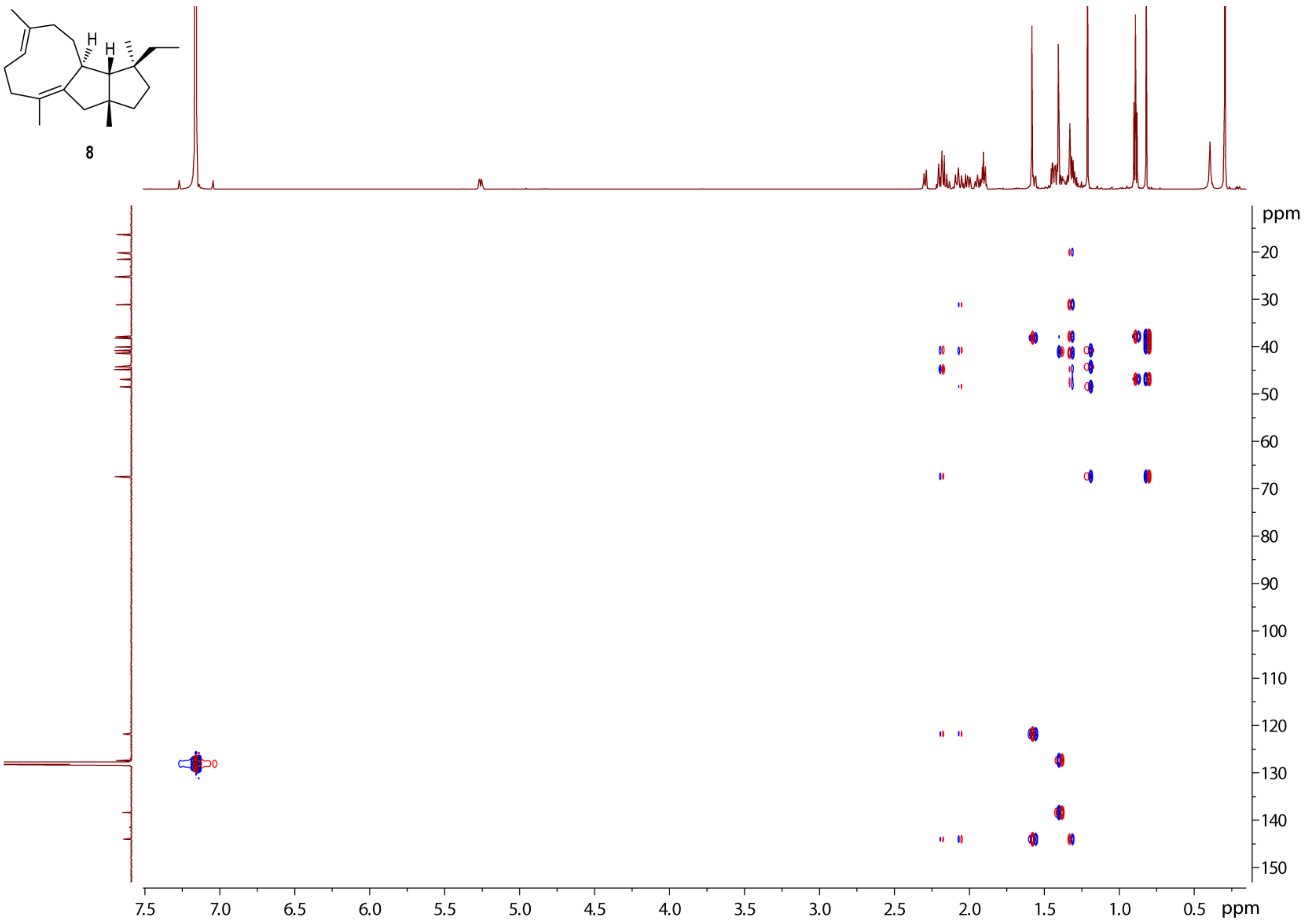

Figure S7. HMBC spectrum of **8** (C_6D_6).

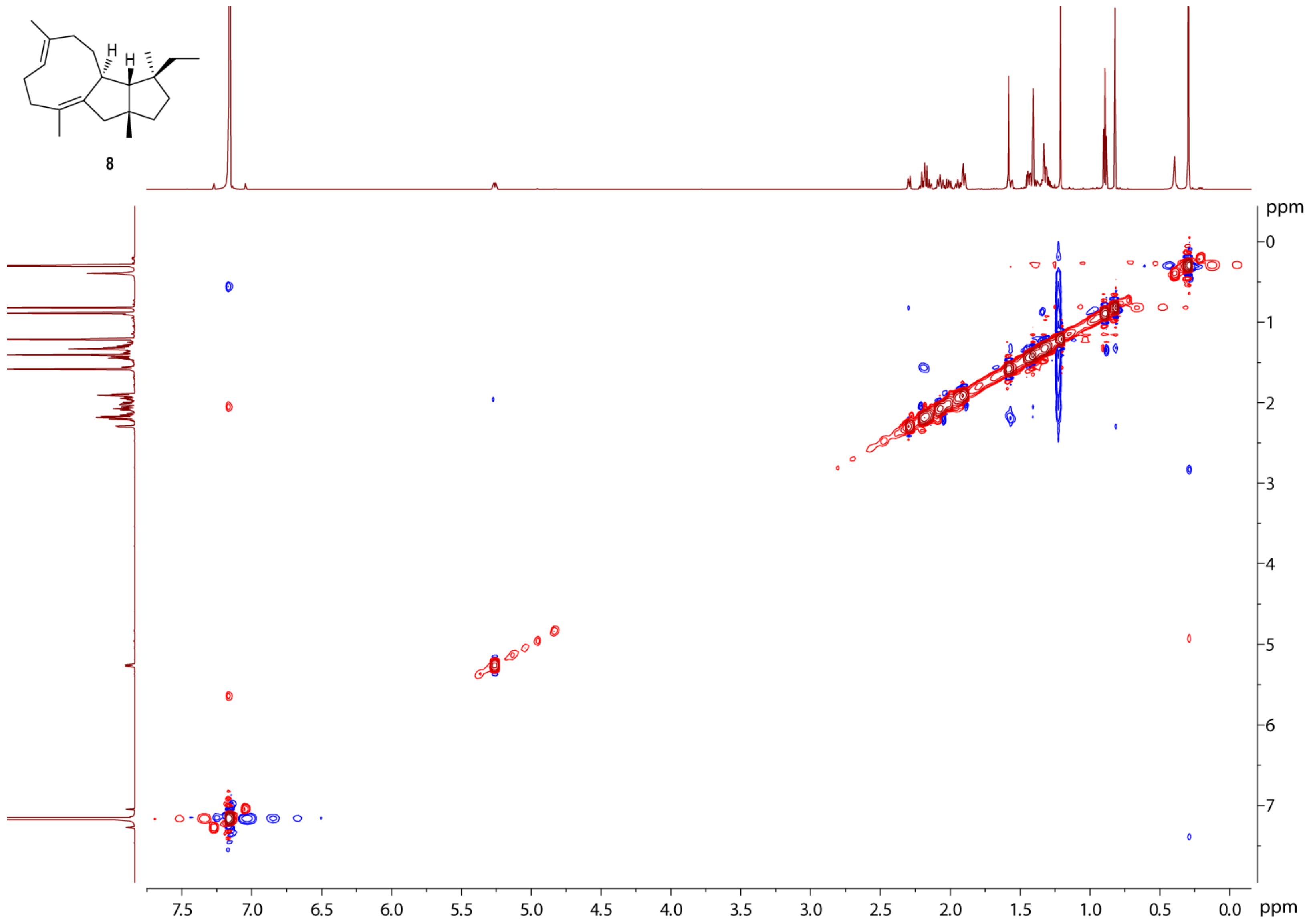

Figure S8. NOESY spectrum of **8** (C_6D_6).

Homodolabellatriene (10). Yield: 1.0 mg (3.5 μmol, 2.2%), from 50 mg (160.7 μmol) **3** trisammonium salt. TLC (pentane): R_f = 0.66. IR (diamond ATR): ṽ = 2959 (w), 2922 (w), 2872 (w), 2851 (w), 2273 (w), 1738 (w), 1551 (w), 1446 (w), 1373 (w), 1343 (w), 1318 (w), 1263 (w), 1217 (w), 1093 (w), 1015 (w), 845 (w), 813 (w), 740 (w), 705 (w), 540 (m) cm^{-1}. HR-MS (APCI): calc. for $[C_{21}H_{35}]^+$ m/z = 287.2733; found: m/z = 287.2735. Optical rotary power: $[\alpha]_D^{25}$ = +22.0 (c 0.1, C_6H_6).

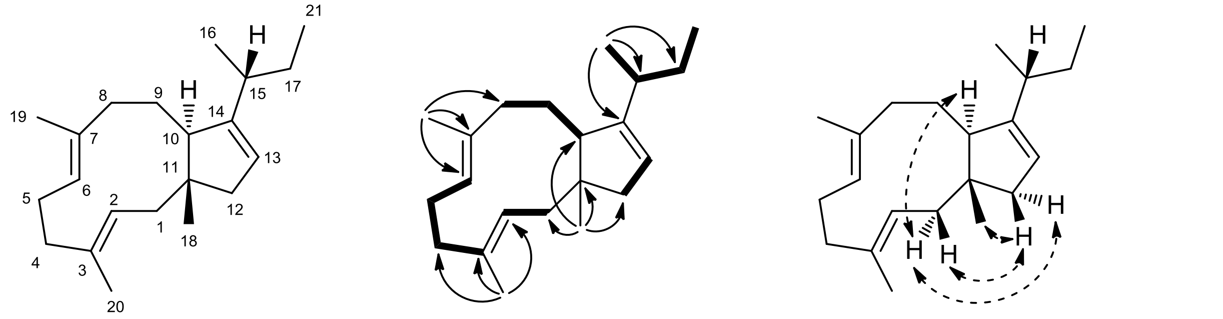

10

Figure S9. Structure elucidation of **10**. Bold: ^{1}H,^{1}H-COSY, single headed arrows: key HMBC, and dashed double headed arrows: key NOESY correlations. Carbon numbering follows GGPP numbering to indicate the origin of each carbon.

Table S2. NMR data of homodolabellatriene (**10**) in C_6D_6 recorded at 298 K.

C[a]	type	^1H[b]	^{13}C[b]
1	CH_2	2.36 (t, $^2J_{H,H}$ = $^3J_{H,H}$ = 12.3, H_α)<br>1.70 (dd, $^2J_{H,H}$ = 13.9, $^3J_{H,H}$ = 4.6, H_β)	41.98
2	CH	5.20 (dd, $^3J_{H,H}$ = 11.7, 4.0)	125.79
3	C_q	–	134.2
4	CH_2	2.13 (m, H_α)<br>2.03 (m, H_β)	40.41
5	CH_2	2.23 (m, H_α)<br>1.98 (m, H_β)	24.86
6	CH	4.86(d, $^3J_{H,H}$ = 11.3)	129.25
7	C_q	–	133.04
8	CH_2	2.26 (m, H_β)<br>2.11 (m, H_α)	38.62
9	CH_2	1.55 (m, H_α)<br>1.37 (m, H_β)	26.39
10	CH	2.12 (m)	47.96
11	C_q	–	46.16
12	CH_2	2.28 (m, H_β)<br>2.01 (m, H_α)	49.02
13	CH	5.26 (hex, $^3J_{H,H}$ = 1.4, $^4J_{H,H}$ = 1.4)	120.11
14	C_q	–	152.71
15	CH	2.03 (m)	33.94
16	CH_3	1.10 (d, $^3J_{H,H}$ = 6.9)	19.17
17	CH_2	1.58 (m)<br>1.26 (m)	29.16
18	CH_3	1.14 (s)	22.83
19	CH_3	1.49 (br s)	15.27
20	CH_3	1.48 (br s)	16.09
21	CH_3	0.90 (t, $^3J_{H,H}$ = 7.5)	11.30

[a] Carbon numbering as shown in Figure S9. [b] Chemical shifts δ in ppm, multiplicity: s = singlet, d = doublet, t = triplet, hex = hextet, m = multiplet, br = broad, coupling constants J are given in Hertz.

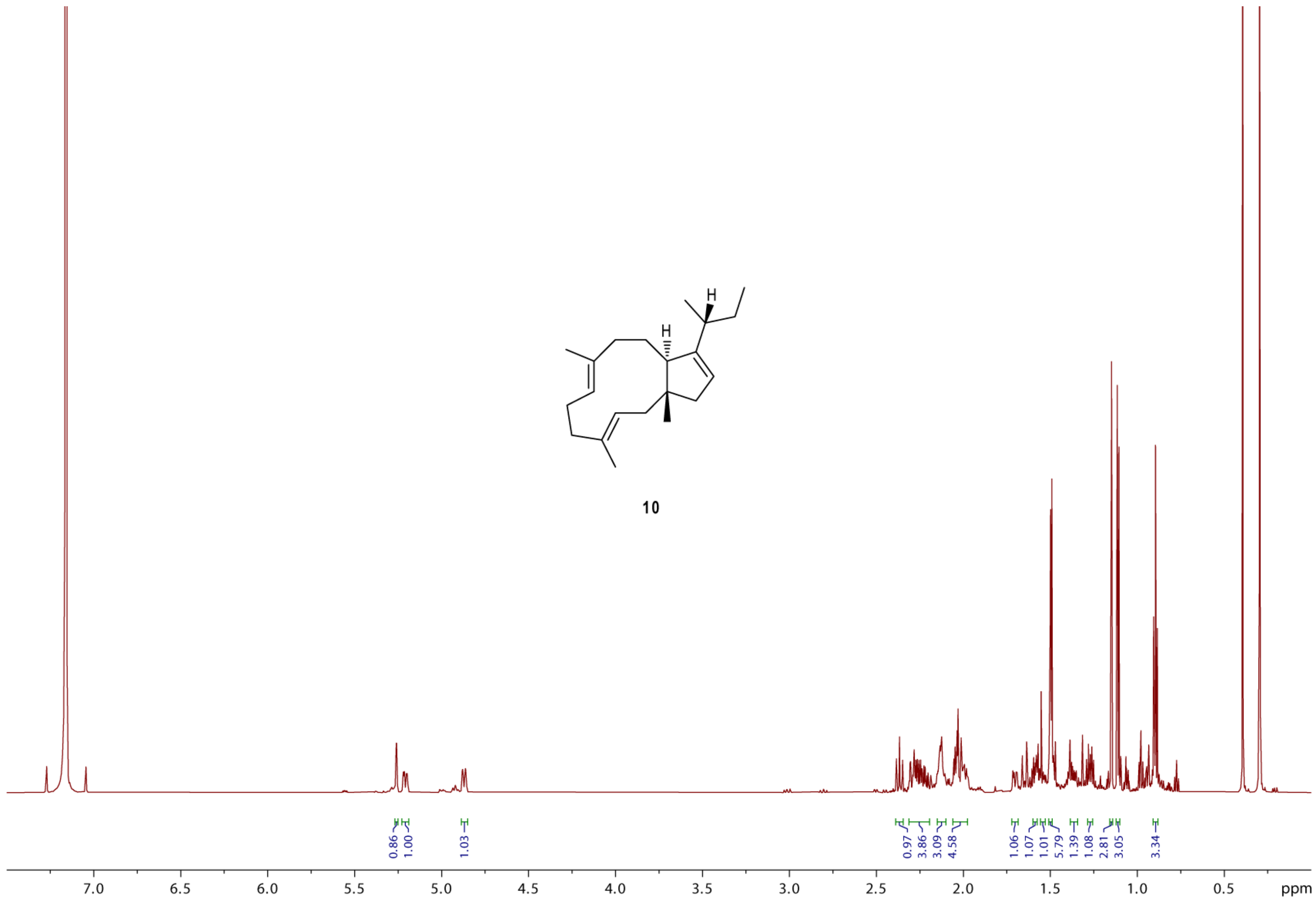

Figure S10. ^{1}H-NMR spectrum of **10** (700 MHz, C_6D_6).

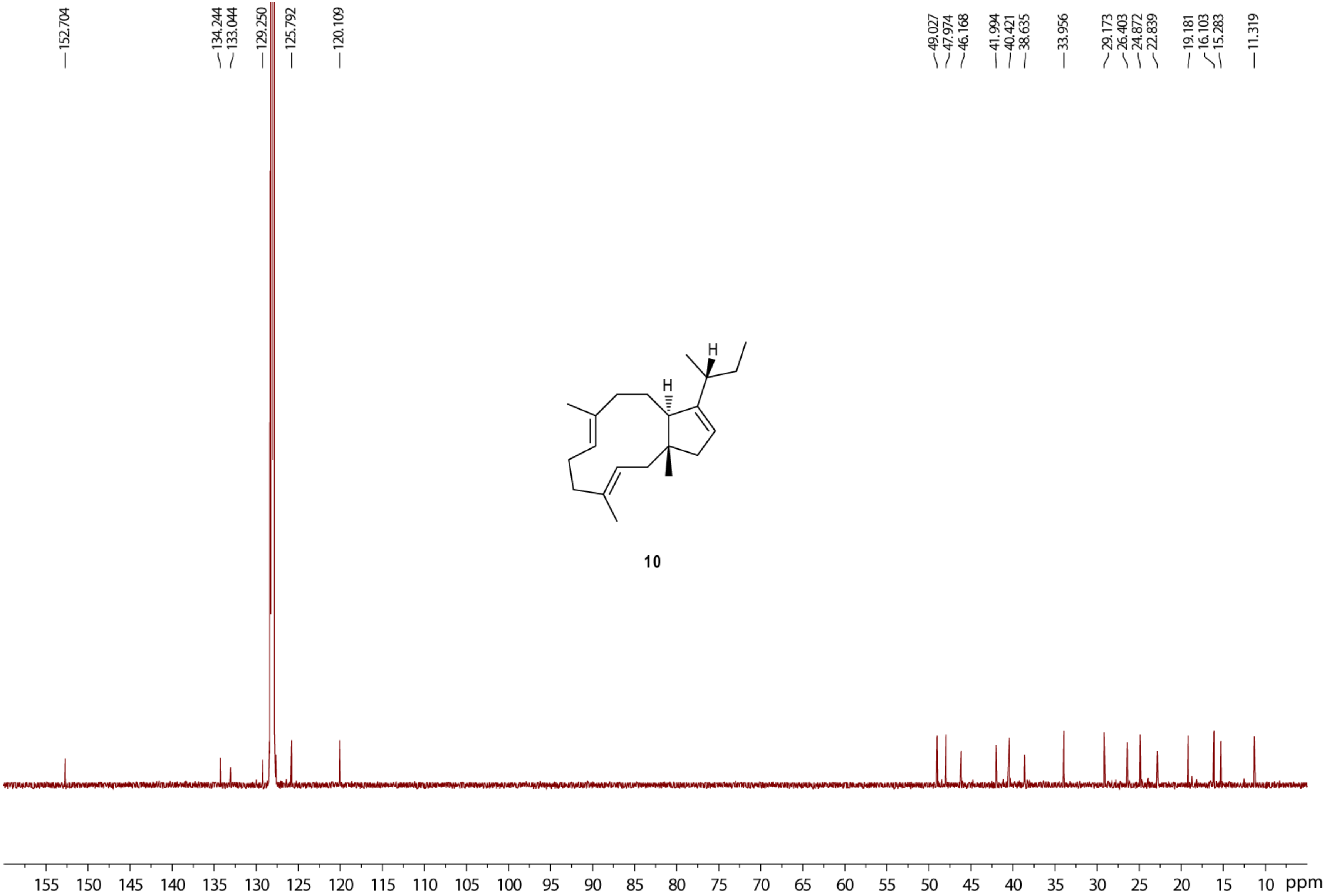

Figure S11. ^{13}C-NMR spectrum of **10** (176 MHz, C_6D_6).

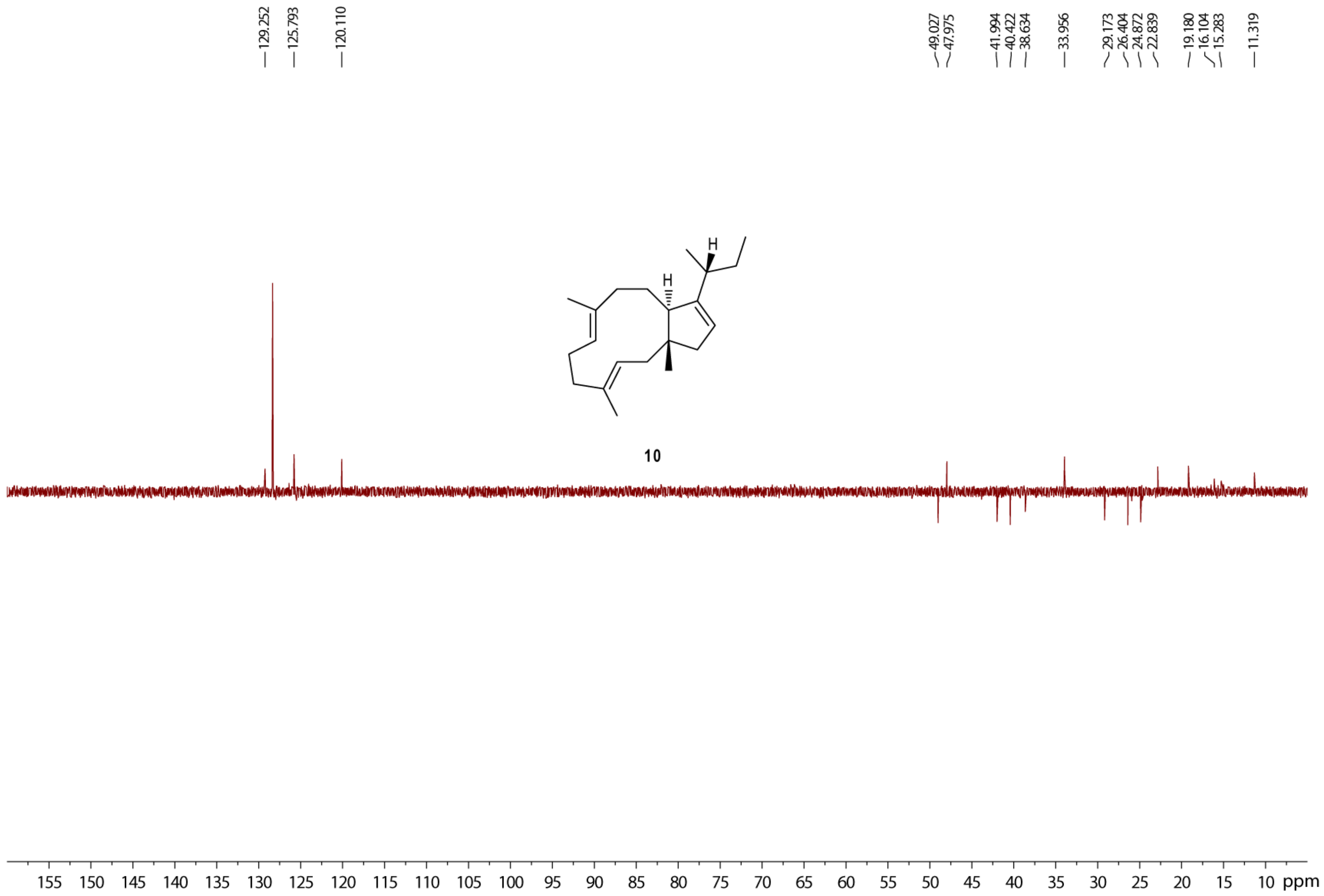

Figure S12. ^{13}C-DEPT spectrum of **10** (176 MHz, C_6D_6).

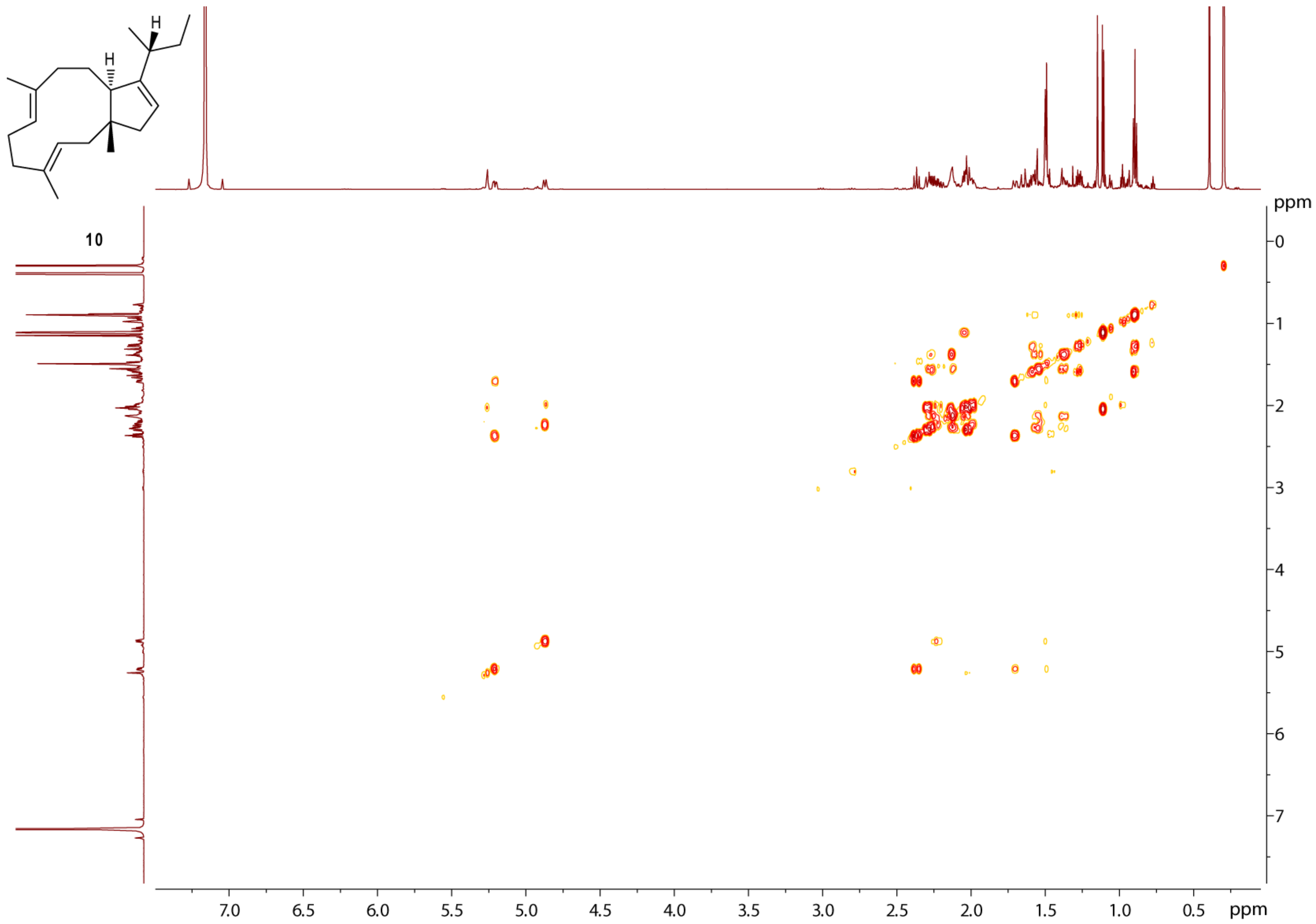

Figure S13. ¹H-¹H-COSY spectrum of **10** (700 MHz, C_6D_6).

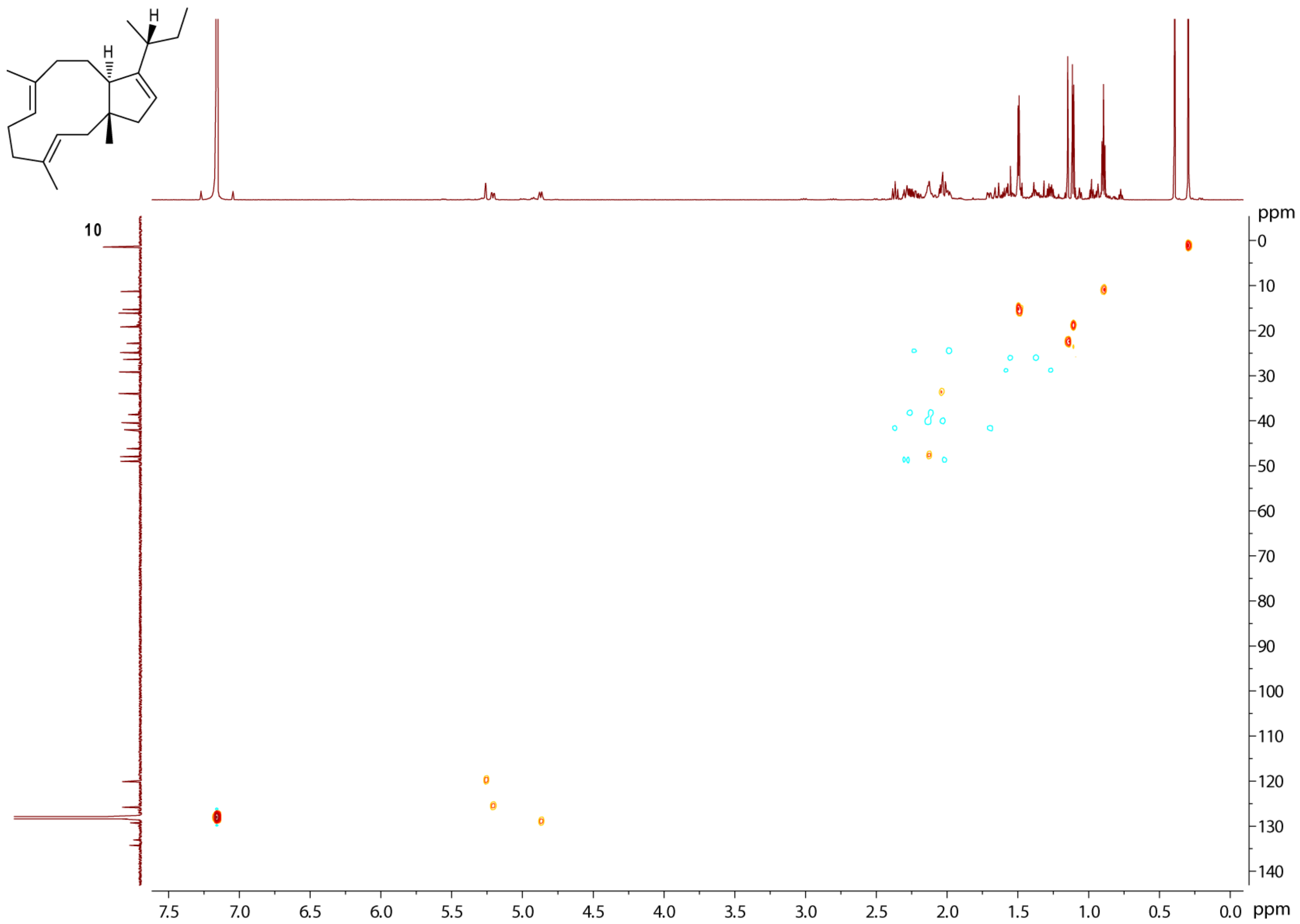

Figure S14. HSQC spectrum of **10** (C_6D_6).

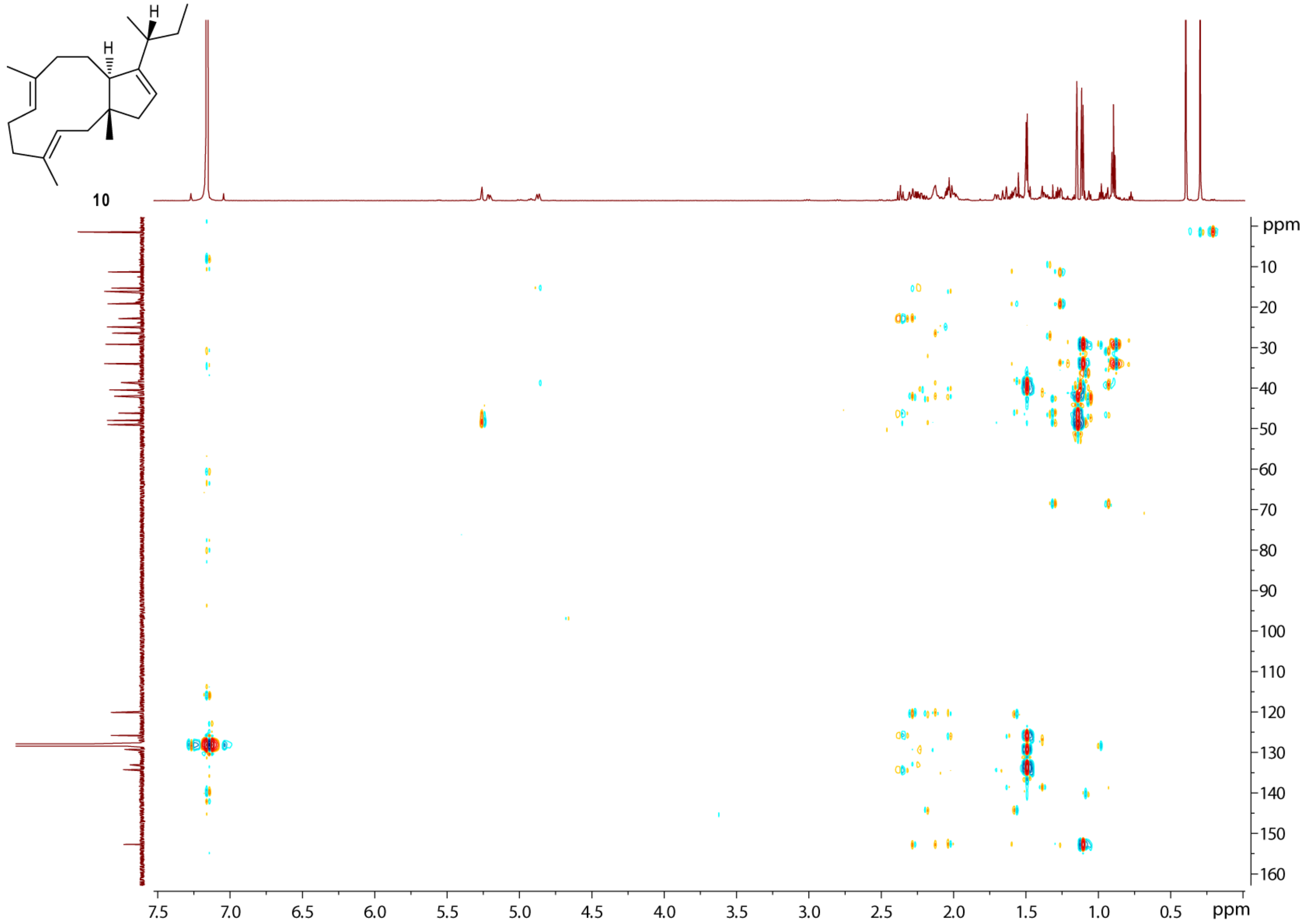

Figure S15. HMBC spectrum of **10** (C_6D_6).

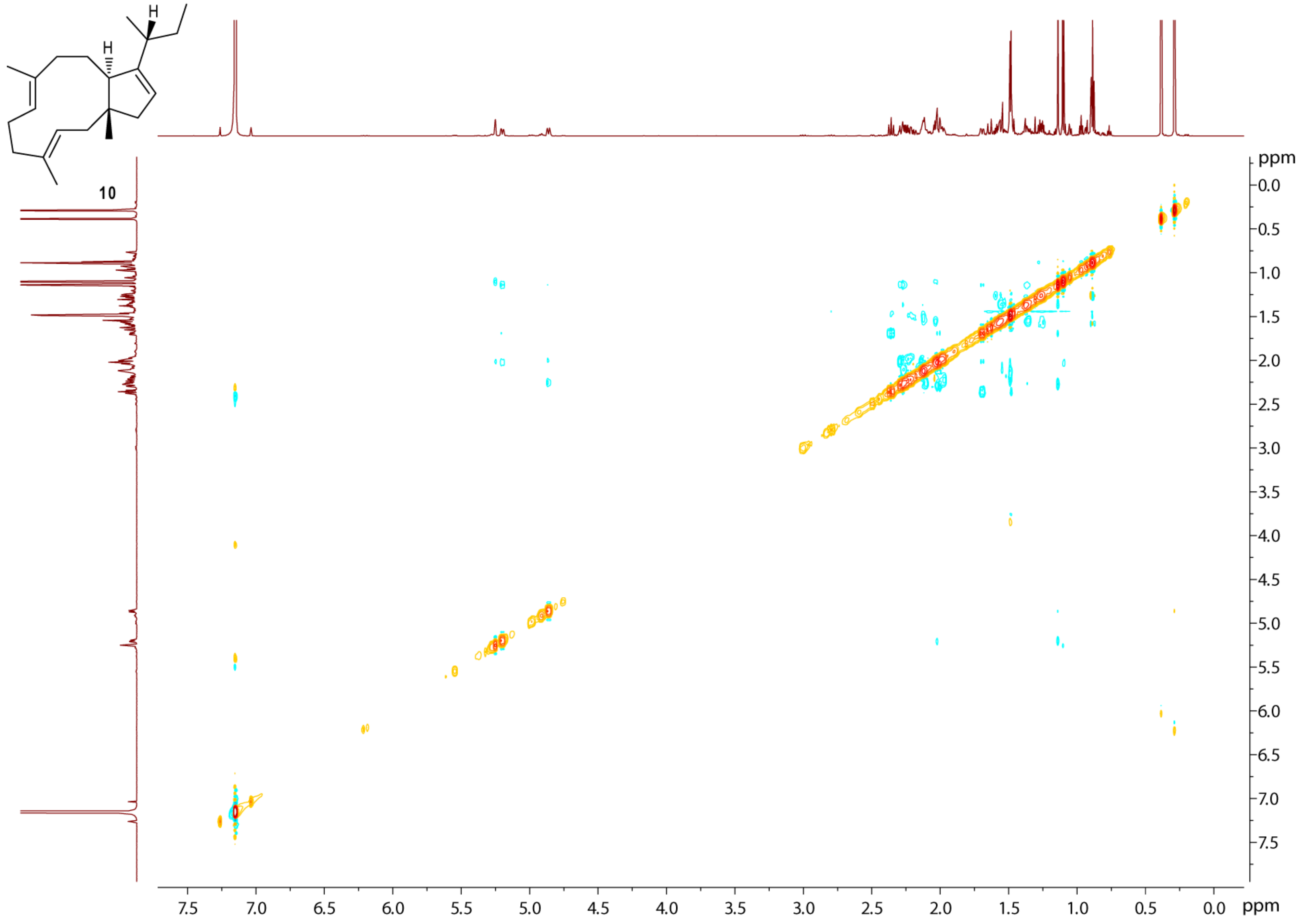

Figure S16. NOESY spectrum of **10** (C_6D_6).

Isohomodolabellatriene I (13). Yield: 2.5 mg (8.7 μmol, 5.4%), from 50 mg (160.7 μmol) **4** trisammonium salt. TLC (pentane): R_f = 0.61. IR (diamond ATR): ṽ = 2960 (m), 2924 (m), 2853 (m), 1636 (w), 1457 (w), 1438 (w), 1362 (w), 1260 (w), 1083 (w), 1016 (w), 896 (w), 797 (m), 681 (w) cm^{-1}. HR-MS (APCI): calc. for $[C_{21}H_{35}]^+$ m/z = 287.2733; found: m/z = 287.2733. Optical rotary power: $[\alpha]_D^{25}$ = +7.6 (c 0.25, C_6H_6).

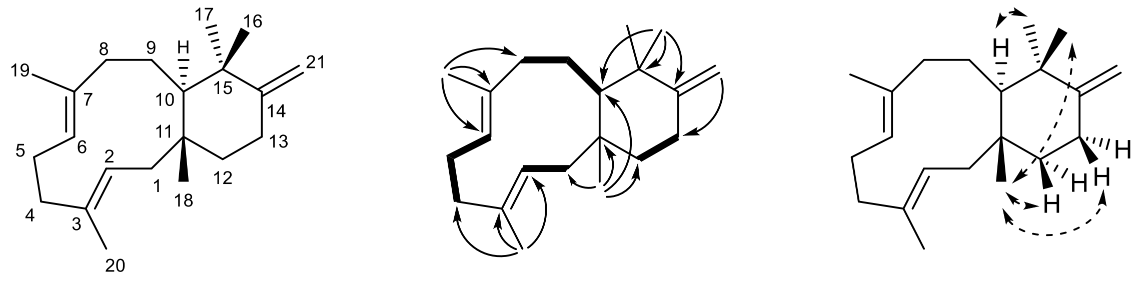

13

Figure S17. Structure elucidation of **13**. Bold: ^{1}H,^{1}H-COSY, single headed arrows: key HMBC, and dashed double headed arrows: key NOESY correlations. Carbon numbering follows GGPP numbering to indicate the origin of each carbon.

Table S3. NMR data of isohomodolabellatriene I (**13**) in C_6D_6 recorded at 298 K.

C[a]	type	^1H[b]	^{13}C[b]
1	CH_2	1.98 (m, H_β)	41.61
		1.71 (m, H_α)	
2	CH	4.78 (m)	125.16
3	C_q	–	131.02
4	CH_2	2.00 (m, H_β)	39.09
		1.73 (m, H_α)	
5	CH_2	2.06 (m, H_β)	26.58
		1,92 (m, H_α)	
6	CH	4.79 (m)	125.66
7	C_q	–	137.55
8	CH_2	2.12 (m, H_β)	42.72
		2.06 (m, H_α)	
9	CH_2	1.31 (m, H_β)	23.01
		1.18 (m, H_β)	
10	CH	1.44 (m)	48.77
11	C_q	–	38.09
12	CH_2	1.73 (m, H_α)	40.14
		1.28 (m, H_β)	
13	CH_2	2.50 (m, H_β)	30.41
		2.12 (m, H_α)	
14	C_q	–	158.32
15	C_q	–	41.68
16	CH_3	1.05 (s)	23.86
17	CH_3	1.16 (s)	30.01
18	CH_3	0.89 (s)	23.56
19	CH_3	1.34 (s)	16.35
20	CH_3	1,44 (s)	16.99
21	CH_2	4.80 (s)	105.32
		4.77 (m)	

[a] Carbon numbering as shown in Figure S17. [b] Chemical shifts δ in ppm, multiplicity: s = singlet, m = multiplet, coupling constants J are given in Hertz.

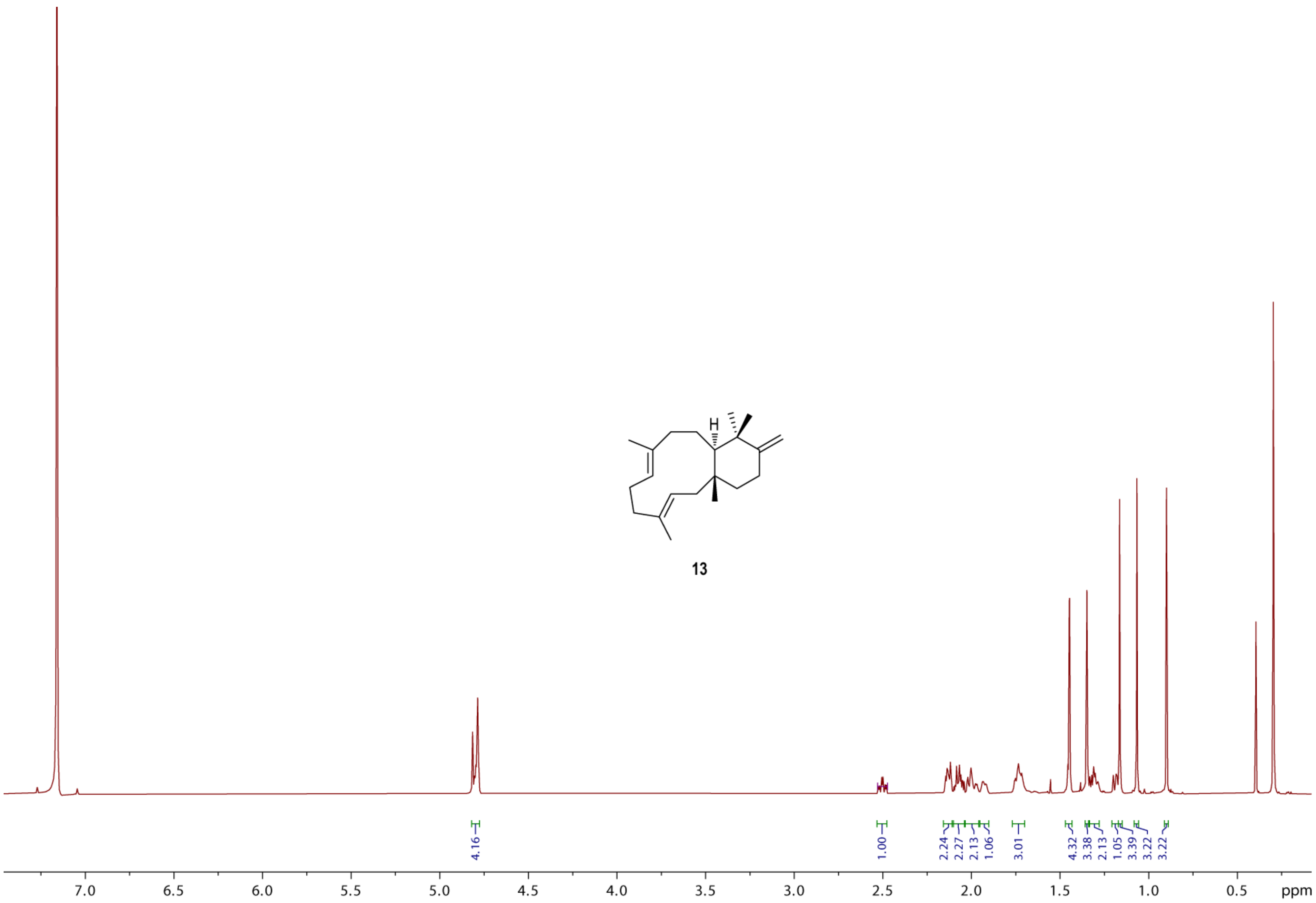

Figure S18. ^{1}H-NMR spectrum of **13** (700 MHz, C_6D_6).

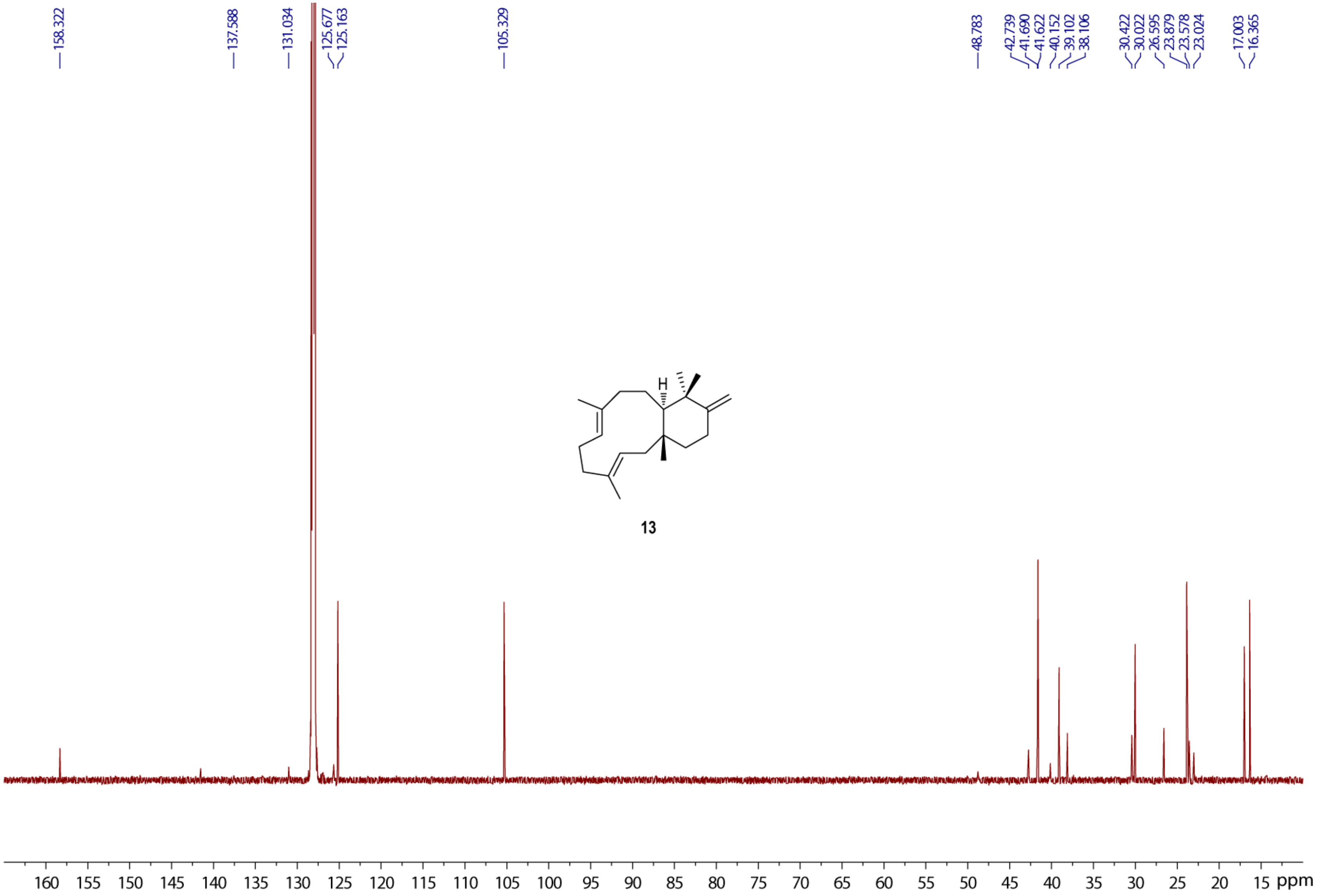

Figure S19. ^{13}C-NMR spectrum of **13** (176 MHz, C_6D_6).

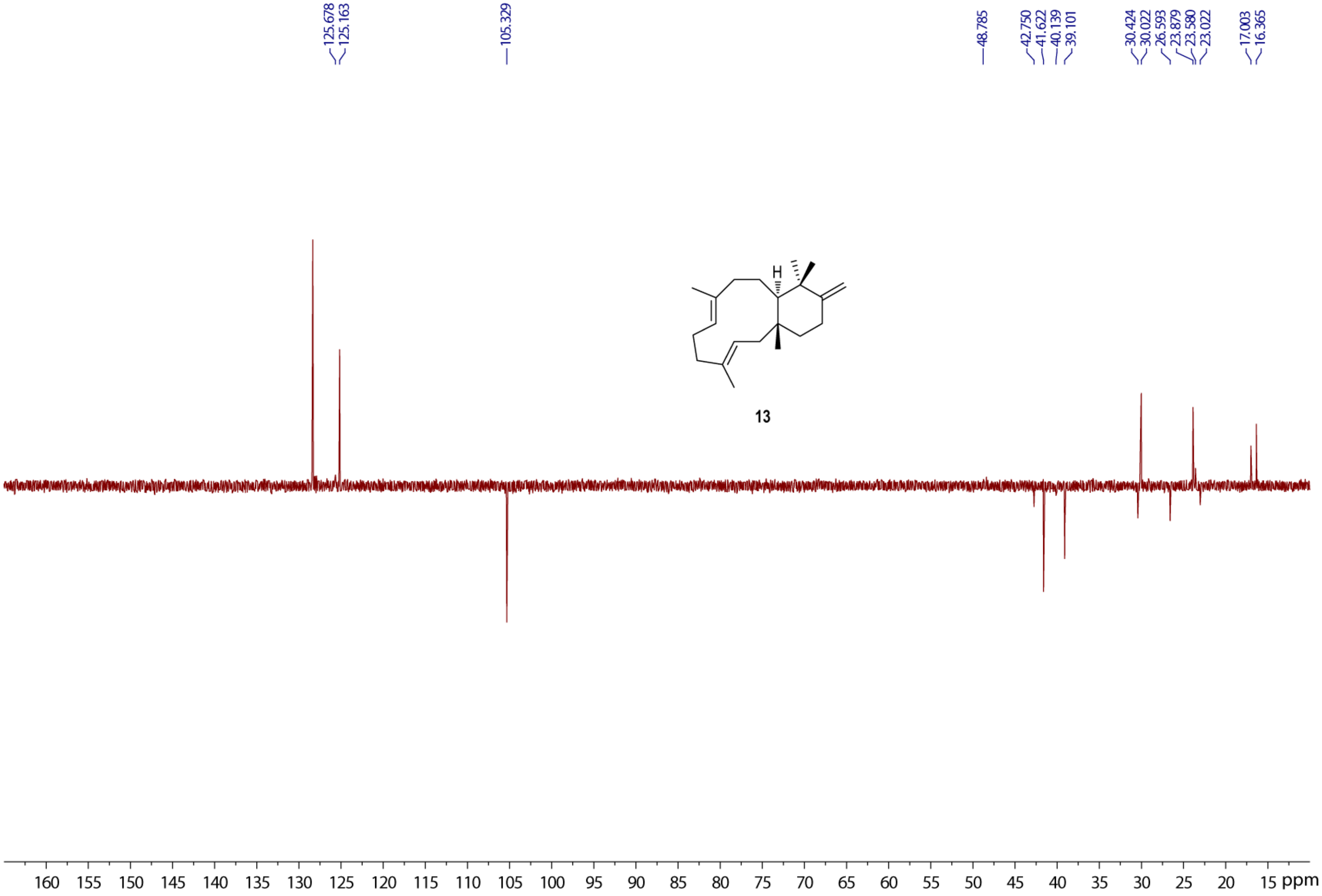

Figure S20. ^{13}C-DEPT spectrum of **13** (176 MHz, C_6D_6).

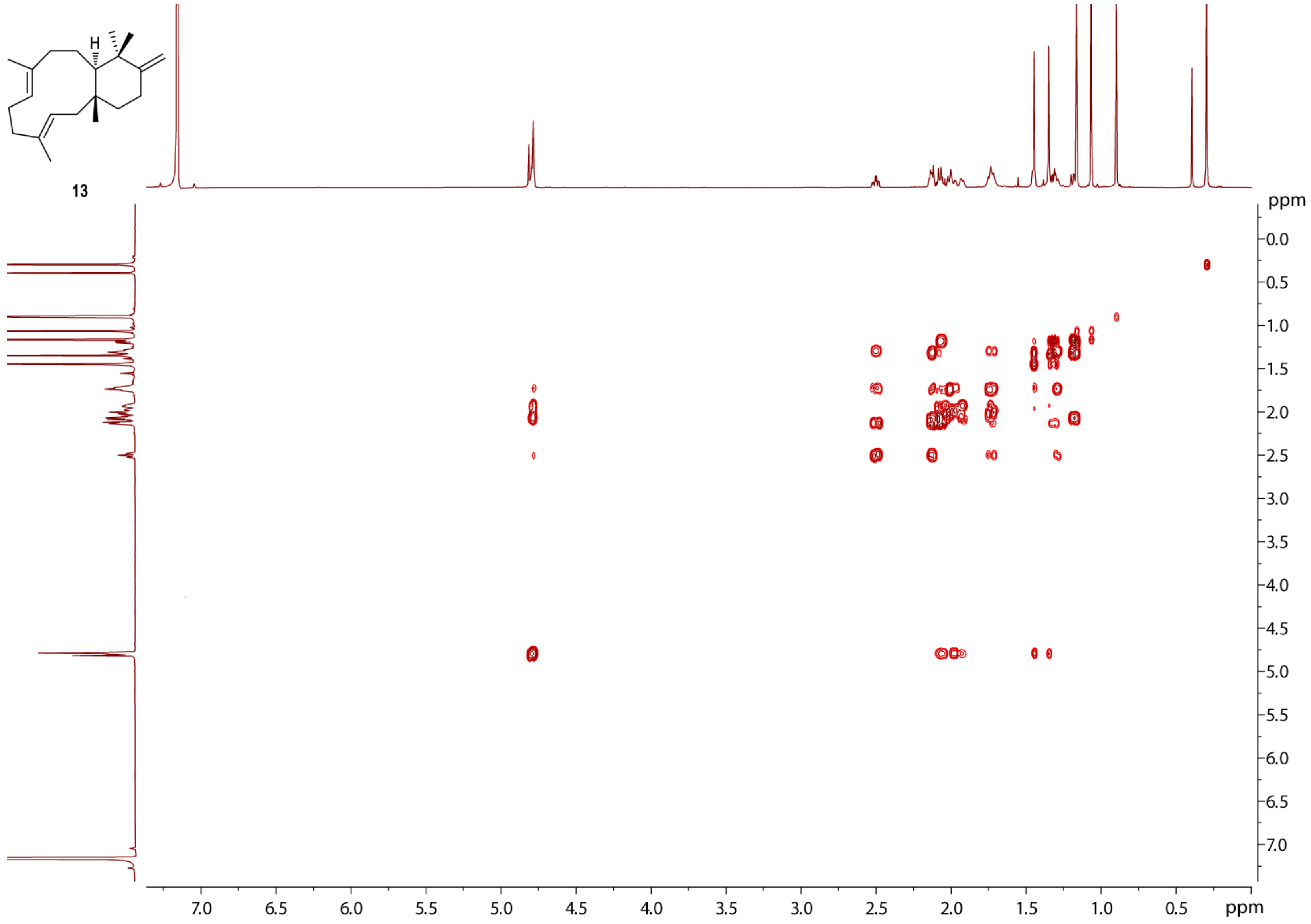

Figure S21. ^{1}H-^{1}H-COSY spectrum of **13** (700 MHz, C_6D_6).

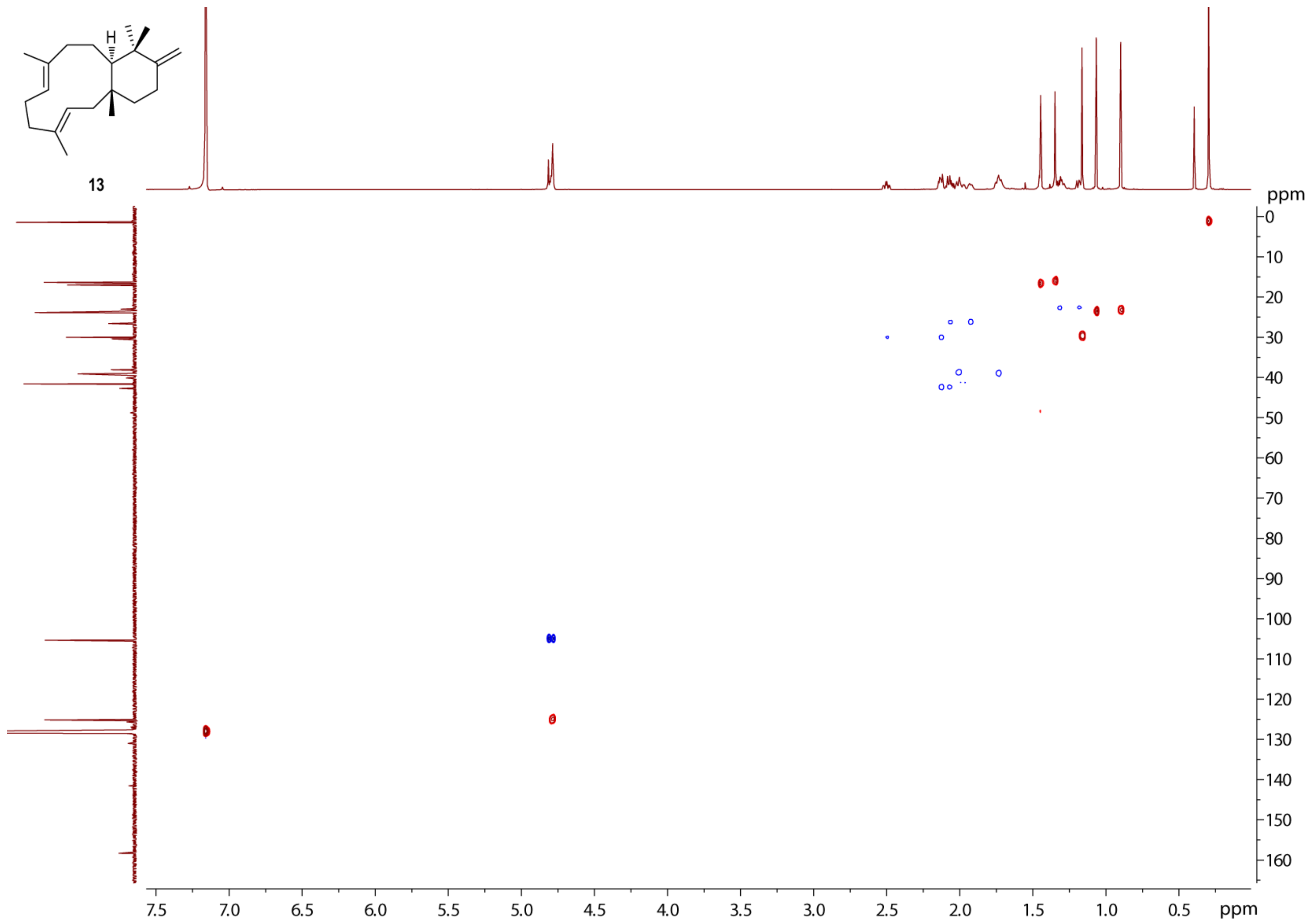

Figure S22. HSQC spectrum of **13** (C_6D_6).

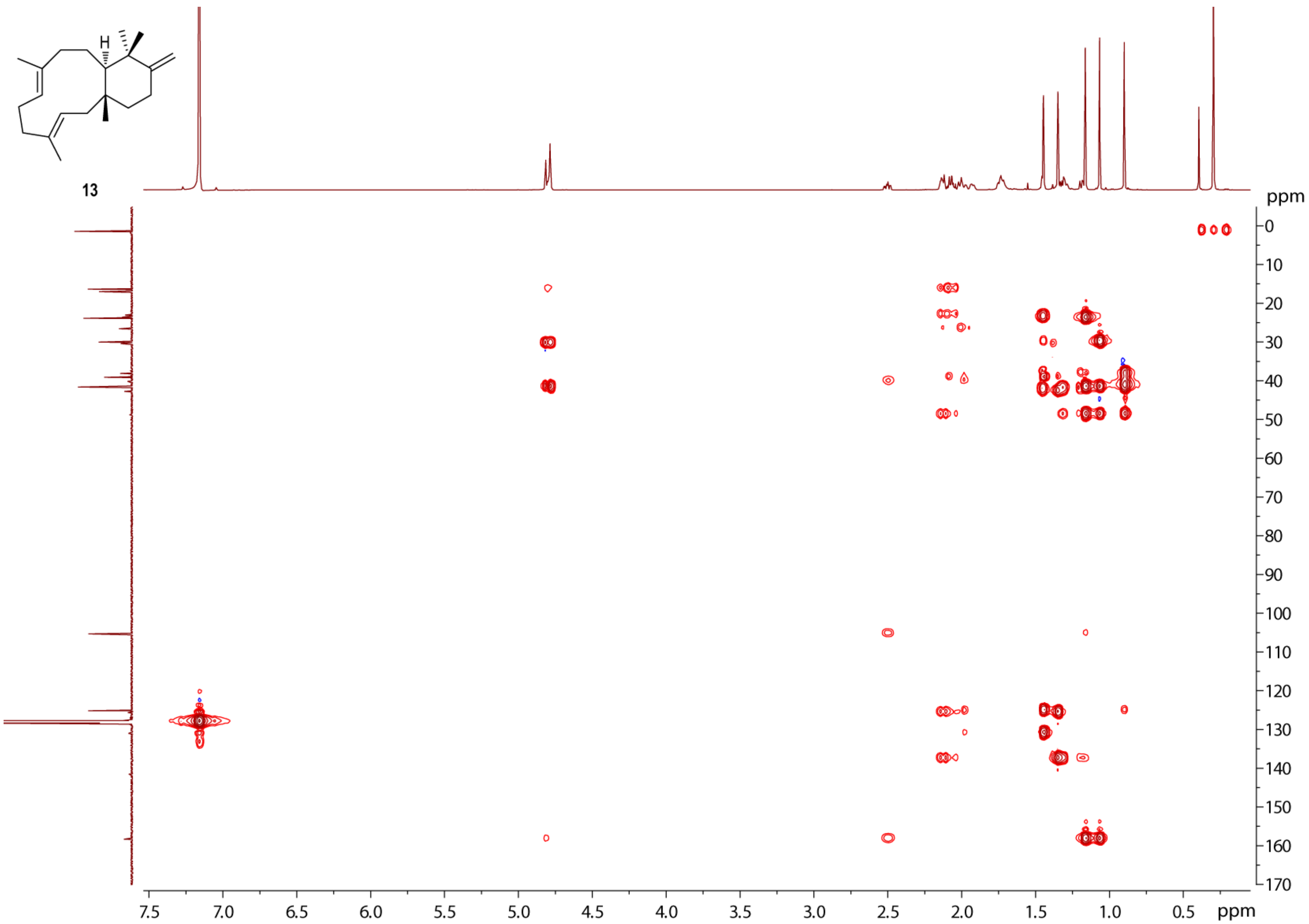

Figure S23. HMBC spectrum of **13** (C_6D_6).

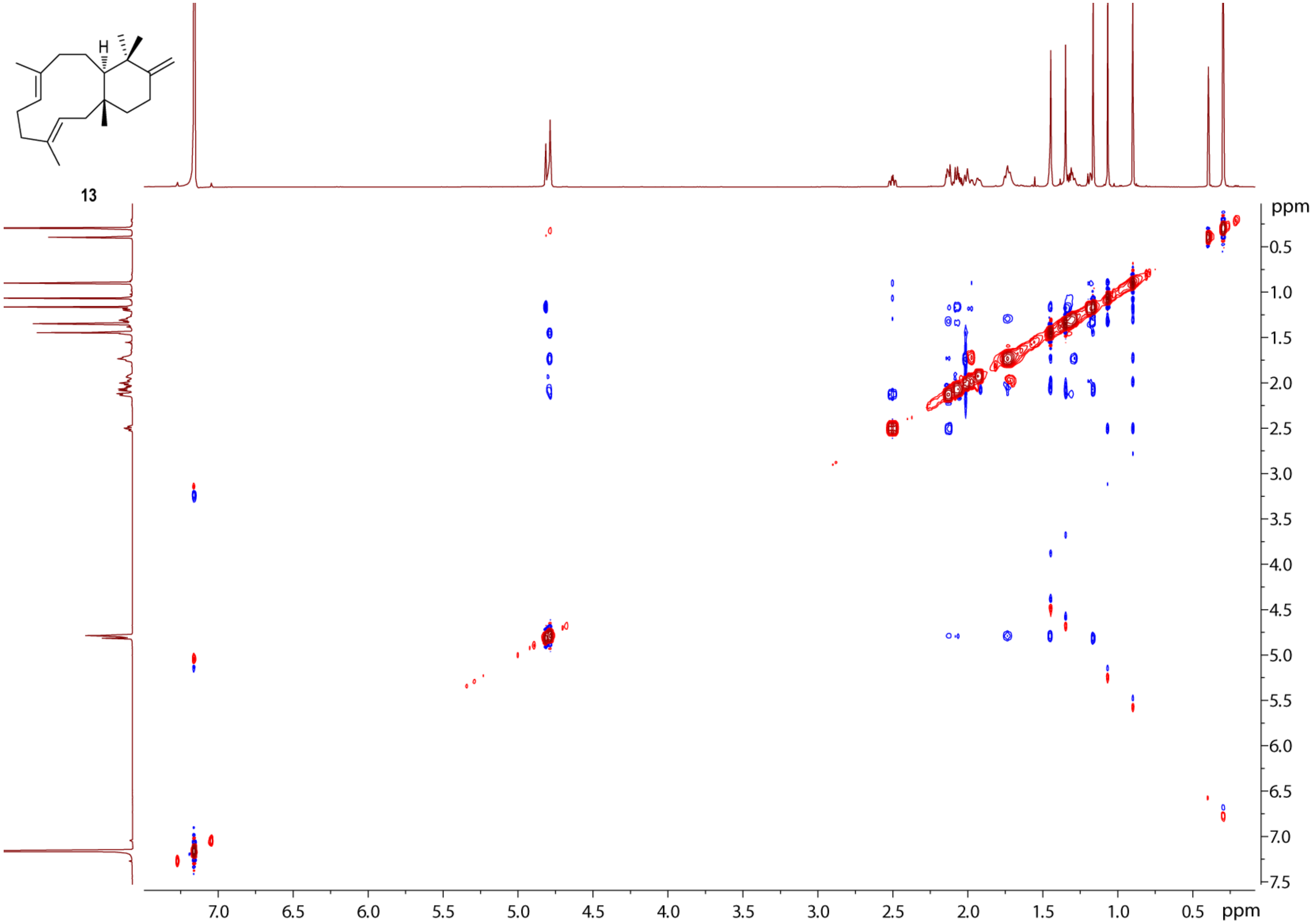

Figure S24. NOESY spectrum of **13** (C_6D_6).

Isohomodolabellatriene II (14). Yield: 1.2 mg (4.2 μmol, 2.6%), from 50 mg (160.7 μmol) **4** trisammonium salt. TLC (pentane): R_f = 0.75. IR (diamond ATR): ṽ = 2955 (m), 2924 (m), 2855 (m), 1733 (w), 1633 (w), 1438 (w), 1378 (w), 1362 (w), 1243 (w), 1094 (w), 1021 (w), 893 (w), 803 (m), 566 (w) cm^{-1}. HR-MS (APCI): calc. for $[C_{21}H_{35}]^+$ *m*/*z* = 287.2733; found: *m*/*z* = 287.2733. Optical rotary power: $[\alpha]_D^{25}$ = +14.0 (*c* 0.1, C_6H_6).

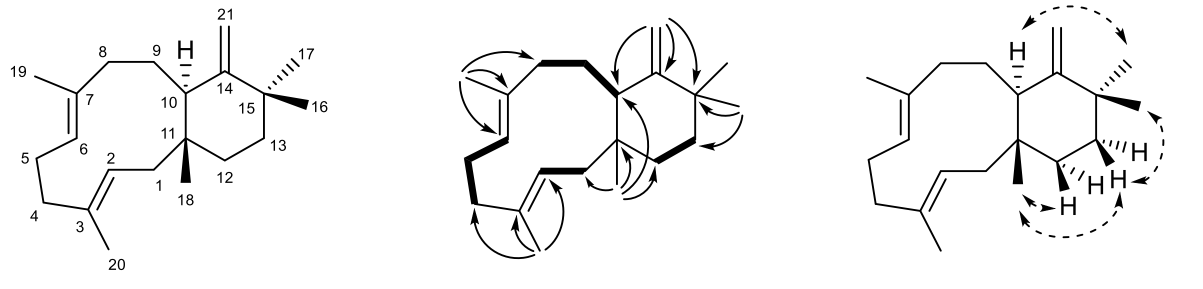

14

Figure S25. Structure elucidation of **14**. Bold: $^1H,^1H$-COSY, single headed arrows: key HMBC, and dashed double headed arrows: key NOESY correlations. Carbon numbering follows GGPP numbering to indicate the origin of each carbon.

Table S4. NMR data of isohomodolabellatriene II (**14**) in C_6D_6 recorded at 298 K.

C[a]	type	^1H[b]	^{13}C[b]
1	CH_2	2.16 (m, H_β)	40.00
		1.73 (m, H_α)	
2	CH	5.20 (br d, $^3J_{H,H}$ = 10.0)	124.12
3	C_q	–	134.38
4	CH_2	2.16 (m, H_β)	39.89
		2.00 (m, H_α)	
5	CH_2	2.27 (m, H_β)	25.06
		2.00 (m, H_α)	
6	CH	4.88 (dd, $^3J_{H,H}$ = 11.3, 3.9)	123.89
7	C_q	–	138.05
8	CH_2	1.96 (dd, $^2J_{H,H}$ = 12.7, $^3J_{H,H}$ = 8.1, H_β)	37.99
		1.70 (m, H_α)	
9	CH_2	1.62 (m, H_α)	25.66
		0.91 (m, H_β)	
10	CH	2.48 (d, $^3J_{H,H}$ = 10.0)	43.40
11	C_q	–	40.04
12	CH_2	2.05 (m, H_α)	34.39
		0.98 (ddd, $^2J_{H,H}$ = 13.8, $^3J_{H,H}$ = 4.8, 3.0, H_β)	
13	CH_2	1.49 (m, H_β)	37.50
		1.27 (ddd, $^2J_{H,H}$ = 13.2, $^3J_{H,H}$ = 4.4, 3.1, H_α)	
14	C_q	–	36.96
15	C_q	–	155.85
16	CH_3	1.14 (s)	30.39
17	CH_3	1.10 (s)	26.50
18	CH_3	0.73 (s)	21.08
19	CH_3	1.43 (t, $^2J_{H,H}$ = 1.4)	15.99
20	CH_3	1.56 (br s)	18.05
21	CH_2	5.05 (dd, $^4J_{H,H}$ = 1.4, $^2J_{H,H}$ = 0.6)	106.02
		4.74 (m)	

[a] Carbon numbering as shown in Figure S25. [b] Chemical shifts δ in ppm, multiplicity: s = singlet, d = doublet, t = triplet, m = multiplet, br = broad, coupling constants J are given in Hertz.

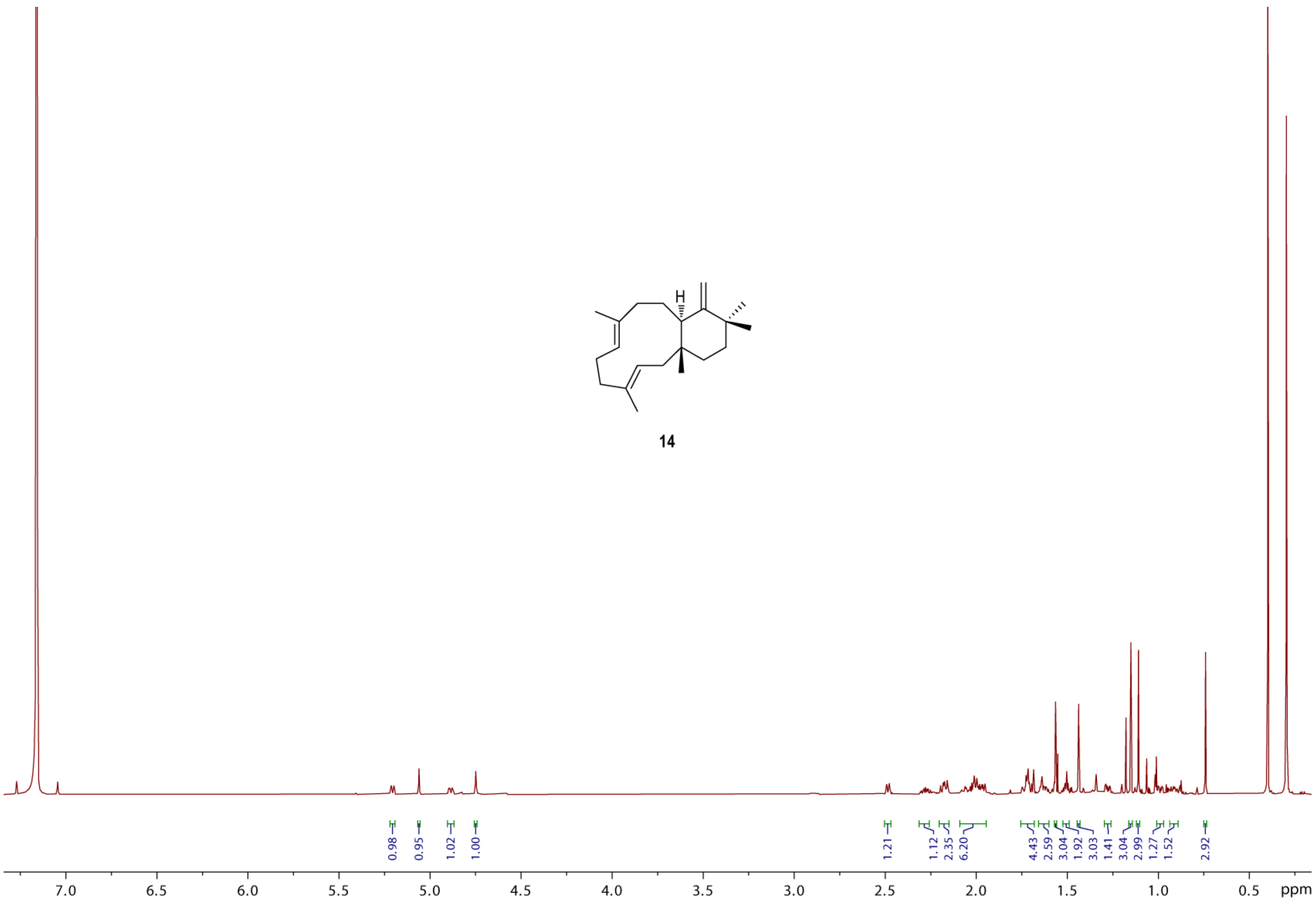

Figure S26. ^{1}H-NMR spectrum of **14** (700 MHz, C_6D_6).

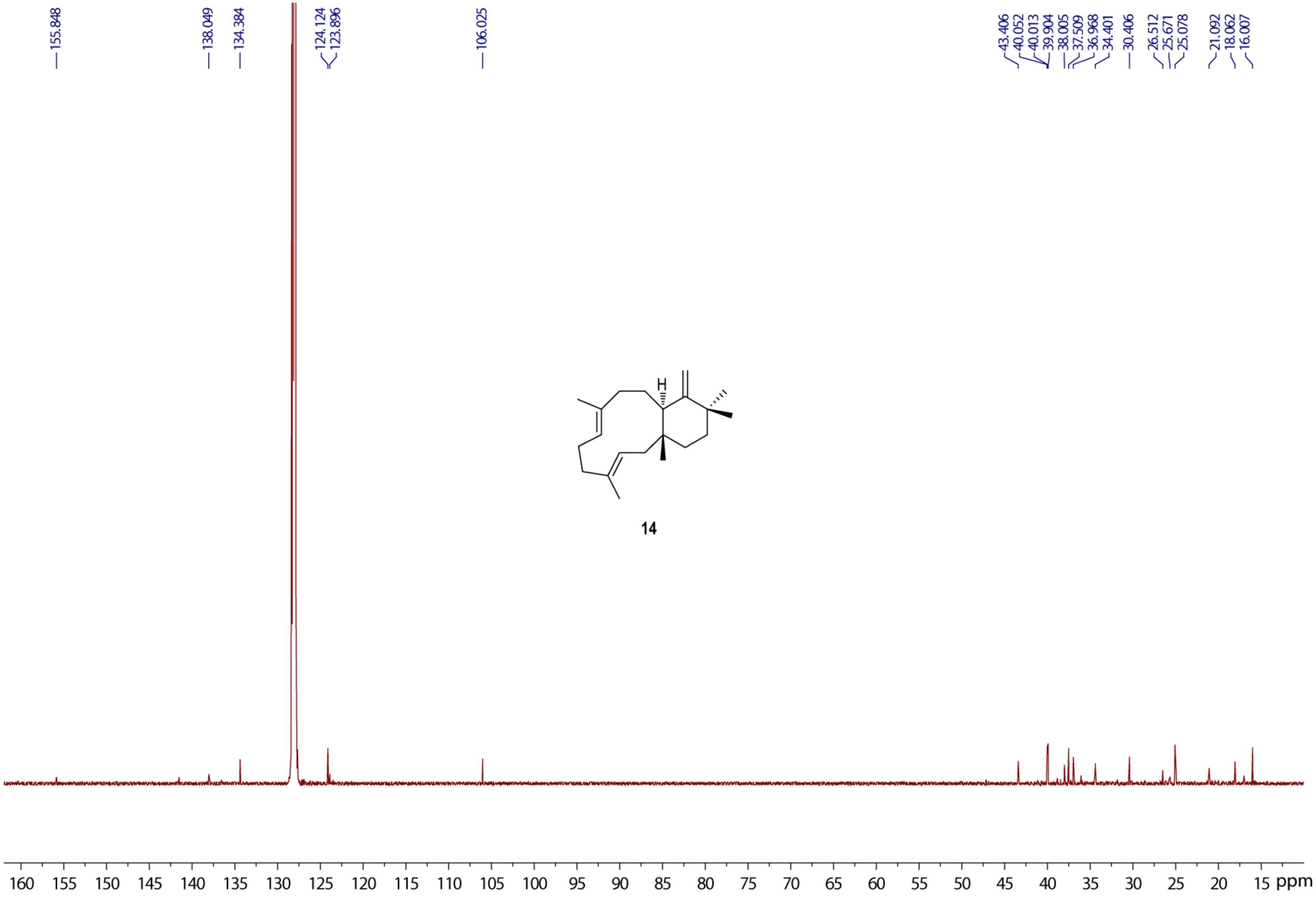

Figure S27. ^{13}C-NMR spectrum of **14** (176 MHz, C_6D_6).

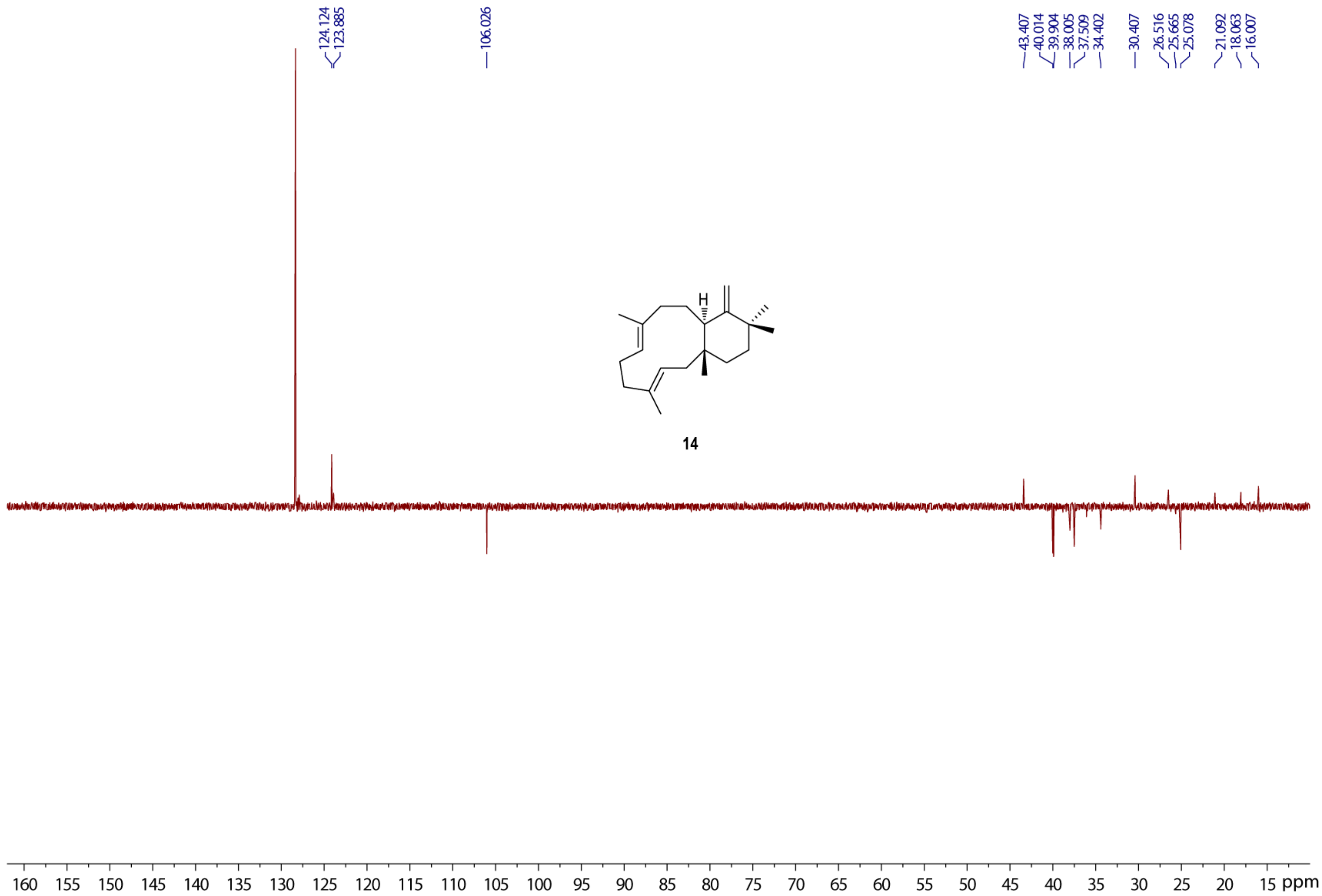

Figure S28. ^{13}C-DEPT spectrum of **14** (176 MHz, C_6D_6).

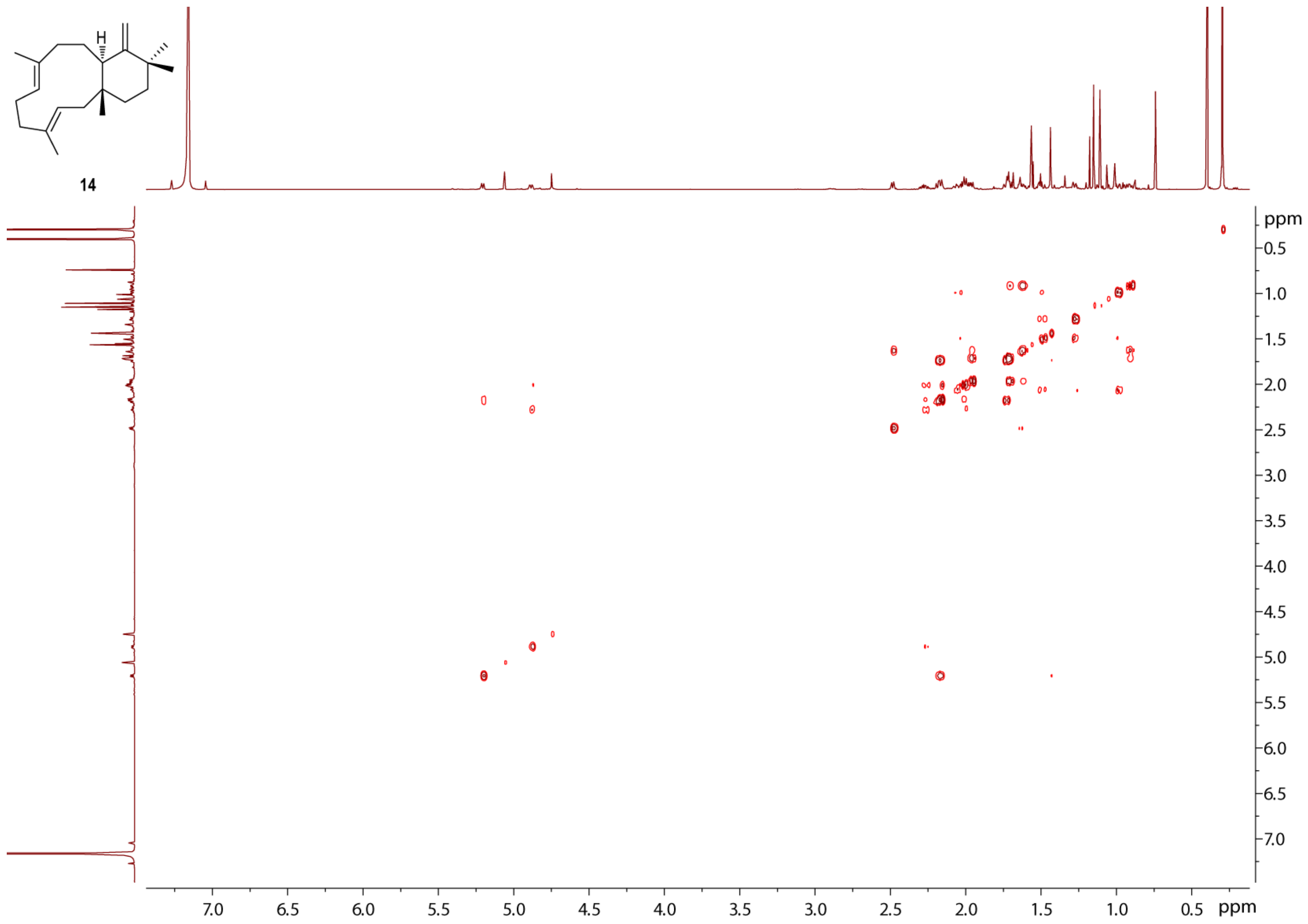

Figure S29. ^{1}H-^{1}H-COSY spectrum of **14** (700 MHz, C_6D_6).

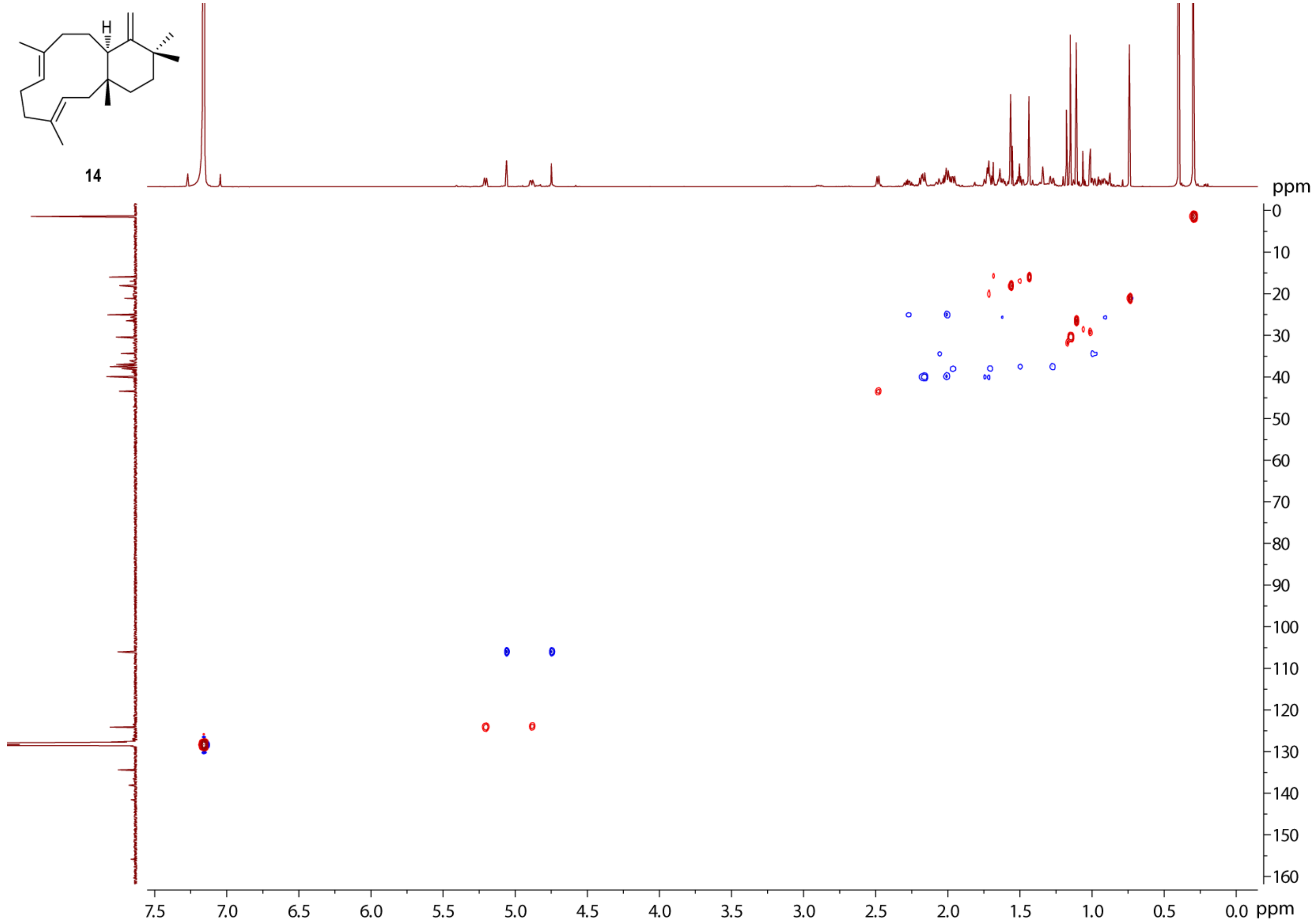

Figure S30. HSQC spectrum of **14** (C_6D_6).

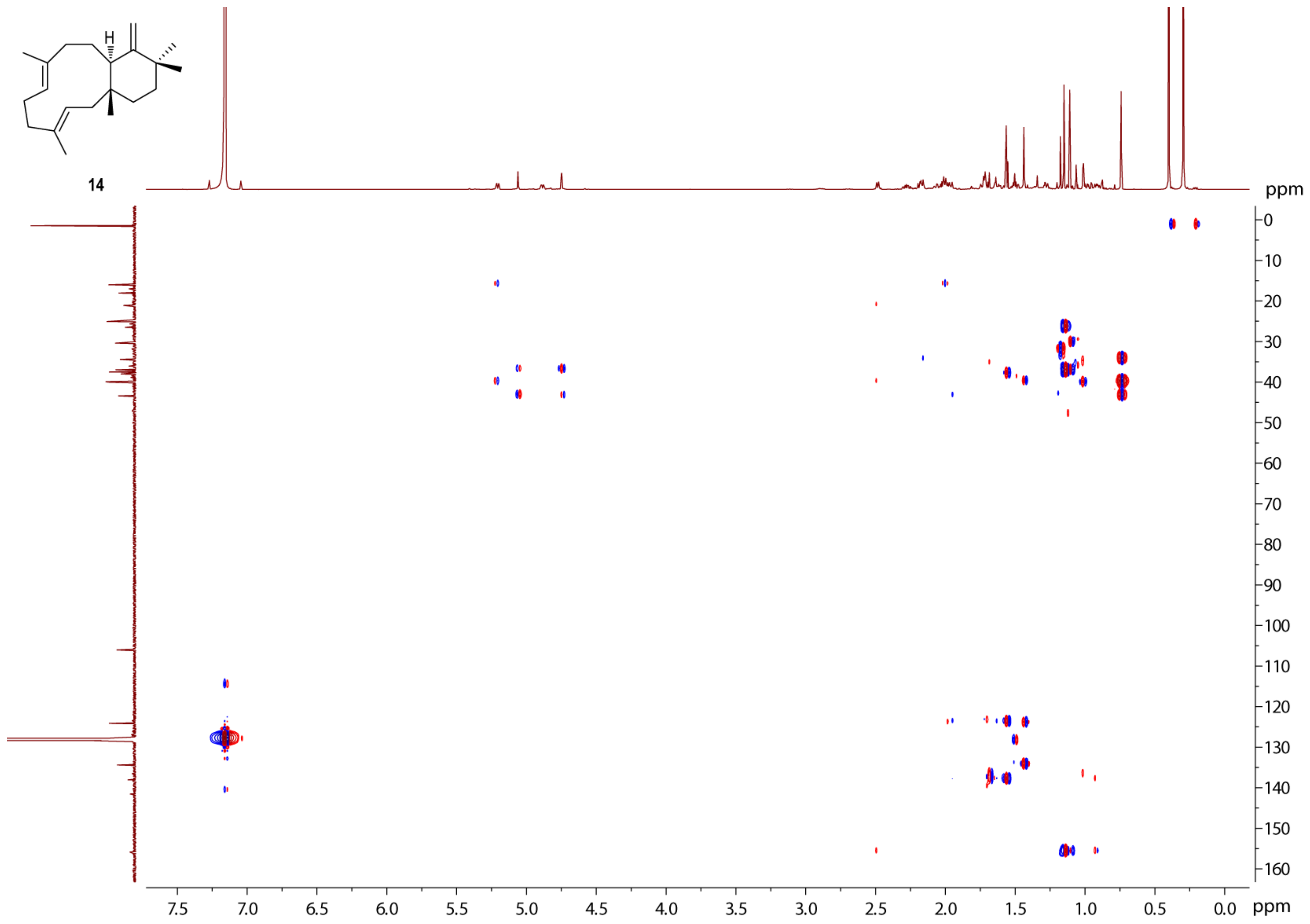

Figure S31. HMBC spectrum of **14** (C_6D_6).

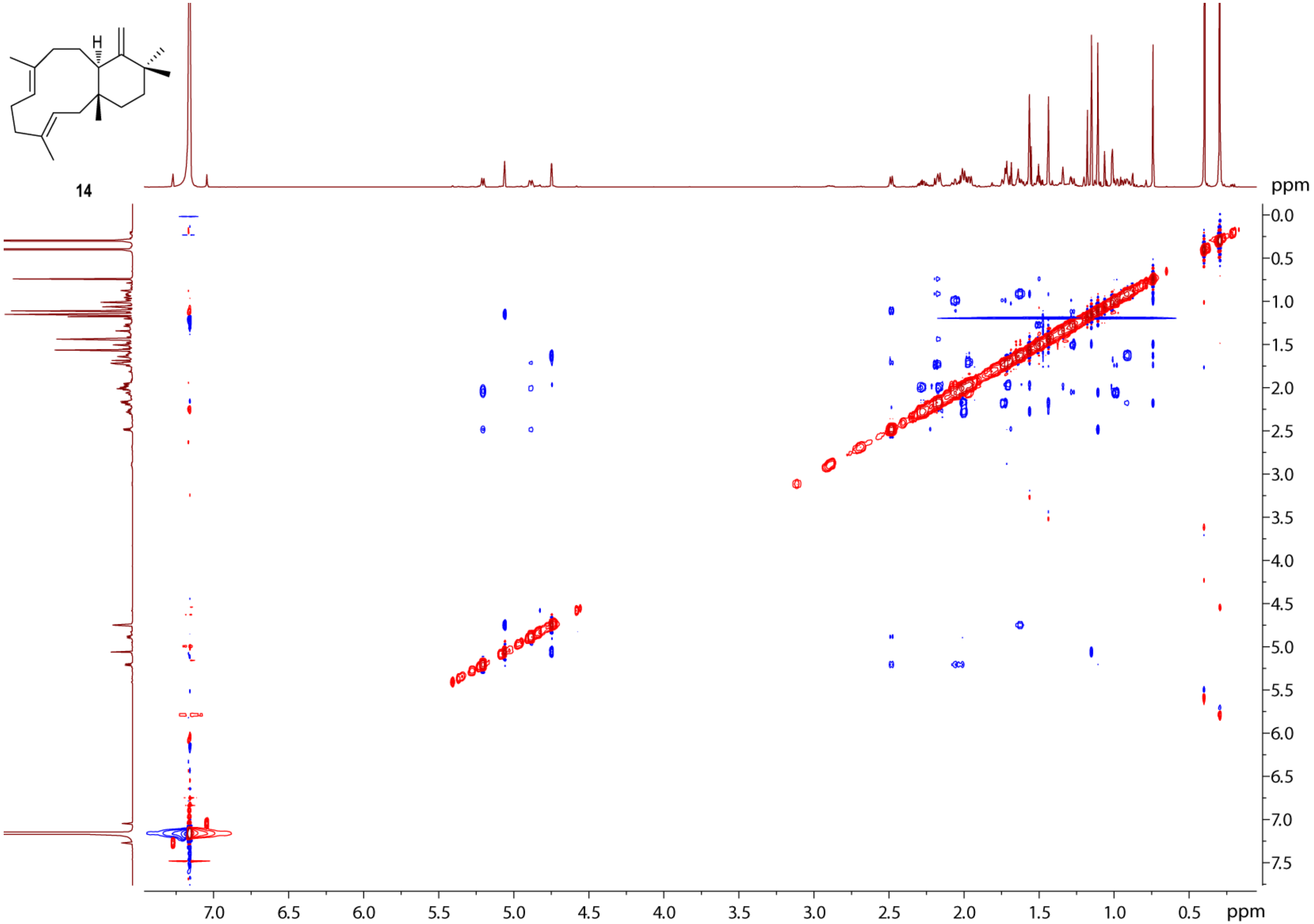

Figure S32. NOESY spectrum of **14** (C_6D_6).

Isodolabellatriene (20). Yield: 0.9 mg (3.3 μmol, 2.0%), from 50 mg (168.3 μmol) **5** trisammonium salt. TLC (pentane): R_f = 0.60. IR (diamond ATR): ṽ = 2963 (w), 2927 (w), 2272 (w), 2253 (w), 1739 (w), 1566 (w), 1370 (w), 1344 (w), 1262 (m), 1092 (m), 1013 (m), 844 (w), 799 (m), 704 (w), 541 (m), 468 (w) cm^{-1}. HR-MS (APCI): calc. for $[C_{20}H_{33}]^+$ *m*/*z* = 273.2577; found: *m*/*z* = 273.2576. Optical rotary power: $[\alpha]_D^{25}$ = −44.4 (*c* 0.09, C_6H_6).

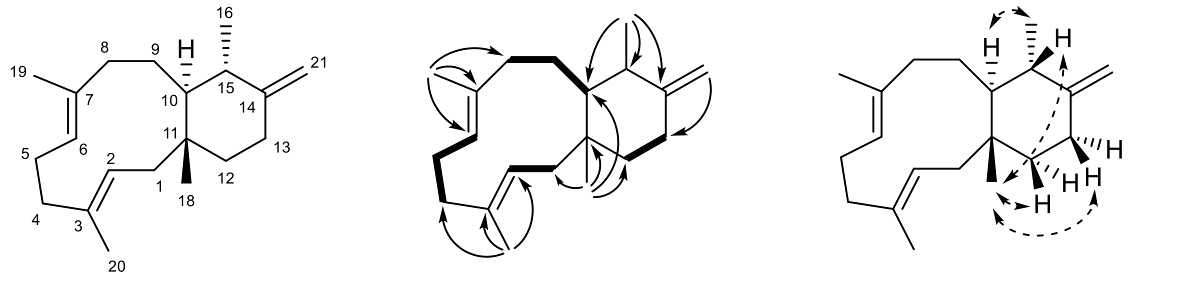

20

Figure S33. Structure elucidation of **20**. Bold: ^{1}H,^{1}H-COSY, single headed arrows: key HMBC, and dashed double headed arrows: key NOESY correlations. Carbon numbering follows GGPP numbering to indicate the origin of each carbon.

Table S5. NMR data of isodolabellatriene (**20**) in C_6D_6 recorded at 298 K.

C[a]	type	^{1}H[b]	^{13}C[b]
1	CH_2	2.08 (m, H_β)	39.76
		1.70 (d, $^2J_{H,H}$ = 17.1, H_α)	
2	CH	4.81 (d, $^3J_{H,H}$ = 7.4)	124.37
3	C_q	–	132.07
4	CH_2	2.01 (m, H_β)	39.11
		1.75 (dd, $^2J_{H,H}$ = 12.6, $^3J_{H,H}$ = 4.8, H_α)	
5	CH_2	2.12 (m, H_β)	26.20
		1.89 (m, H_α)	
6	CH	4.73 (dd, $^3J_{H,H}$ = 11.5, 4.7)	125.11
7	C_q	–	137.11
8	CH_2	2.05 (m, H_β)	41.48
		1.93 (dd, $^2J_{H,H}$ = 12.8, $^3J_{H,H}$ = 10.9, H_α)	
9	CH_2	1.19 (dd, $^2J_{H,H}$ = 14.6, $^3J_{H,H}$ = 10.7, H_β)	27.62
		1.04 (m, H_α)	
10	CH	1.26 (dd, $^3J_{H,H}$ = 10.9, 7.3)	48.14
11	C_q	–	37.94
12	CH_2	1.80 (m, H_α)	40.27
		1.23 (ddd, $^2J_{H,H}$ = 12.8, $^3J_{H,H}$ = 4.5, 2.5, H_β)	
13	CH_2	2.27 (dt, $^2J_{H,H}$ = 13.4, $^3J_{H,H}$ = 4.4, H_β)	33.52
		2.20 (ddd, $^2J_{H,H}$ = 13.2, $^3J_{H,H}$ = 4.8, 2.6, H_α)	
14	C_q	–	154.65
15	CH	1.97 (m)	40.33
16	CH_3	1.15 (d, $^3J_{H,H}$ = 6.7)	17.01
18	CH_3	0.83 (s)	21.07
19	CH_3	1.37 (br s)	16.68
20	CH_3	1.42 (br s)	16.76
21	CH_2	4.82 (q, $^4J_{H,H}$ = 1.6)	104.89
		4.76 (q, $^4J_{H,H}$ = 1.6)	

[a] Carbon numbering as shown in Figure S33. [b] Chemical shifts δ in ppm, multiplicity: s = singlet, d = doublet, t = triplet, q = quartet, m = multiplet, br = broad, coupling constants J are given in Hertz.

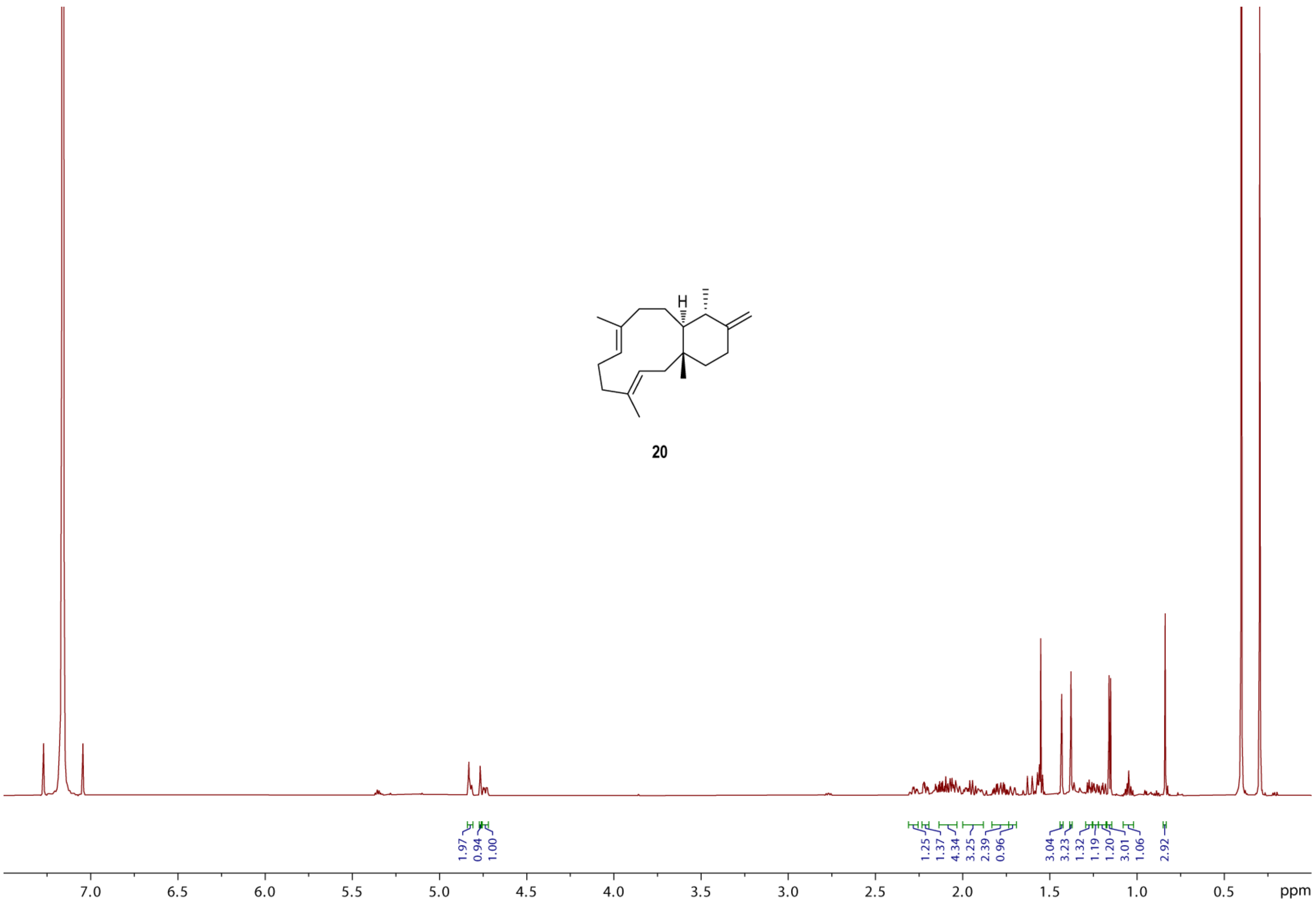

Figure S34. ^{1}H-NMR spectrum of **20** (700 MHz, C_6D_6).

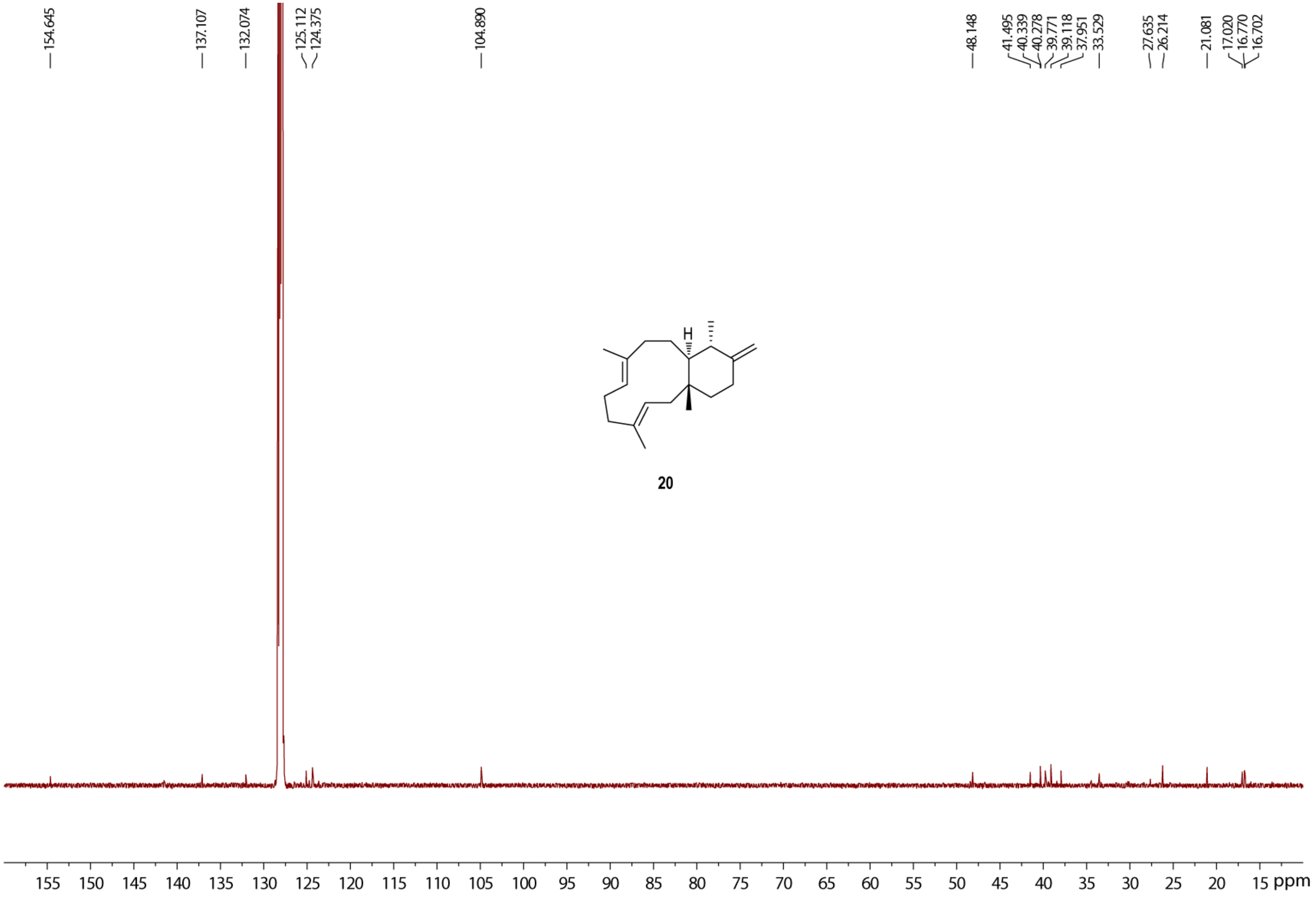

Figure S35. ^{13}C-NMR spectrum of **20** (176 MHz, C_6D_6).

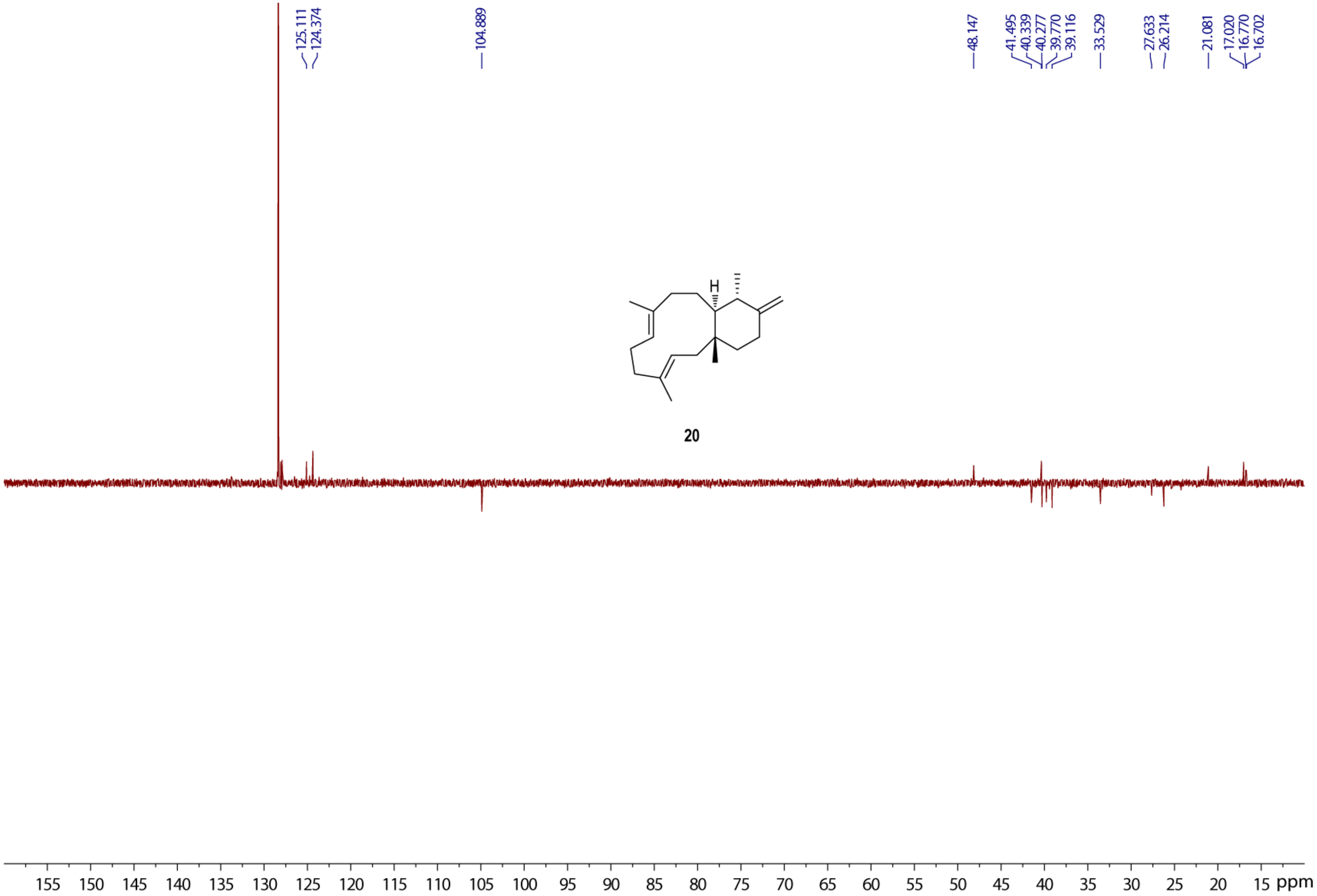

Figure S36. ^{13}C-DEPT spectrum of **20** (176 MHz, C_6D_6).

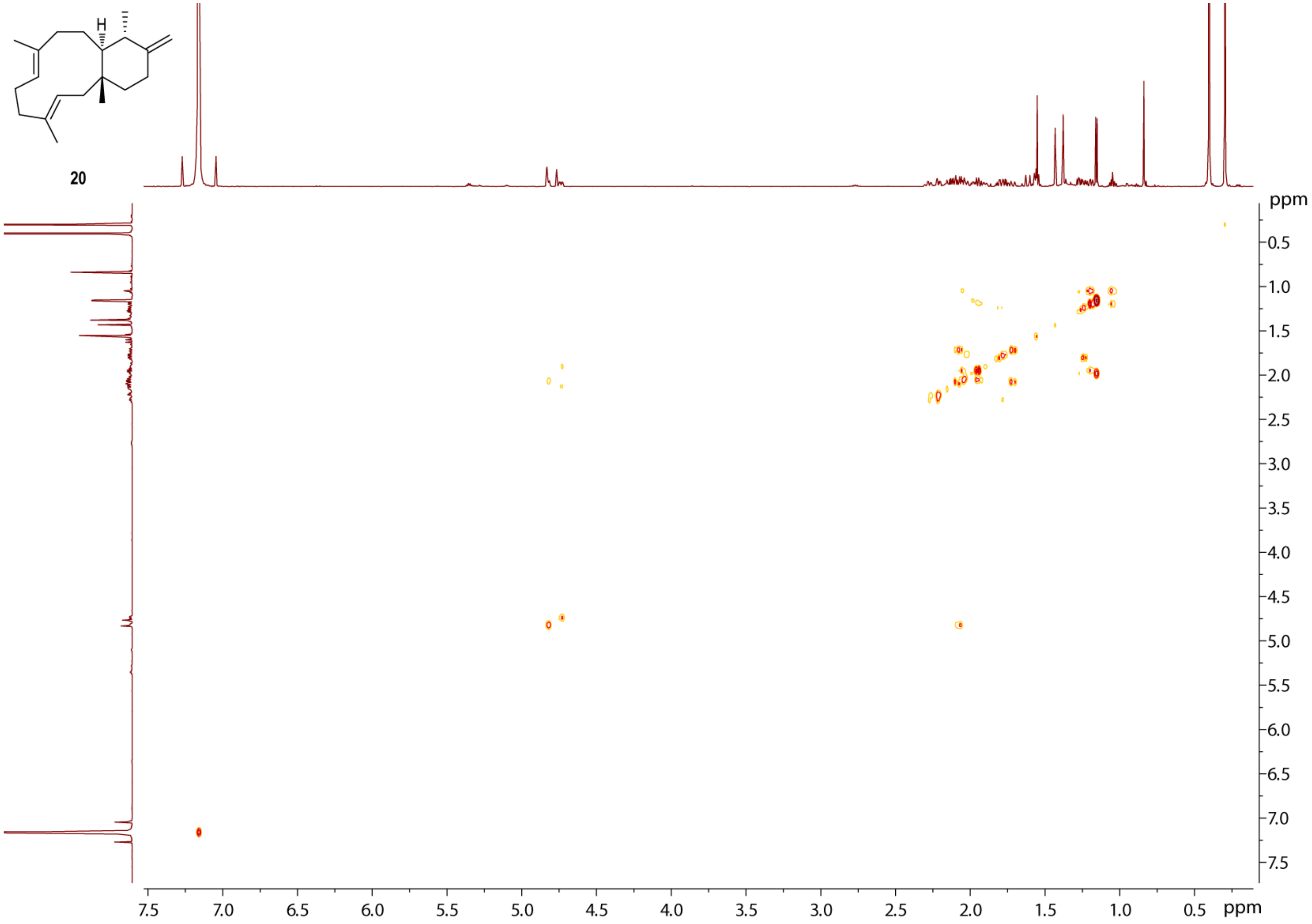

Figure S37. ^{1}H-^{1}H-COSY spectrum of **20** (700 MHz, C_6D_6).

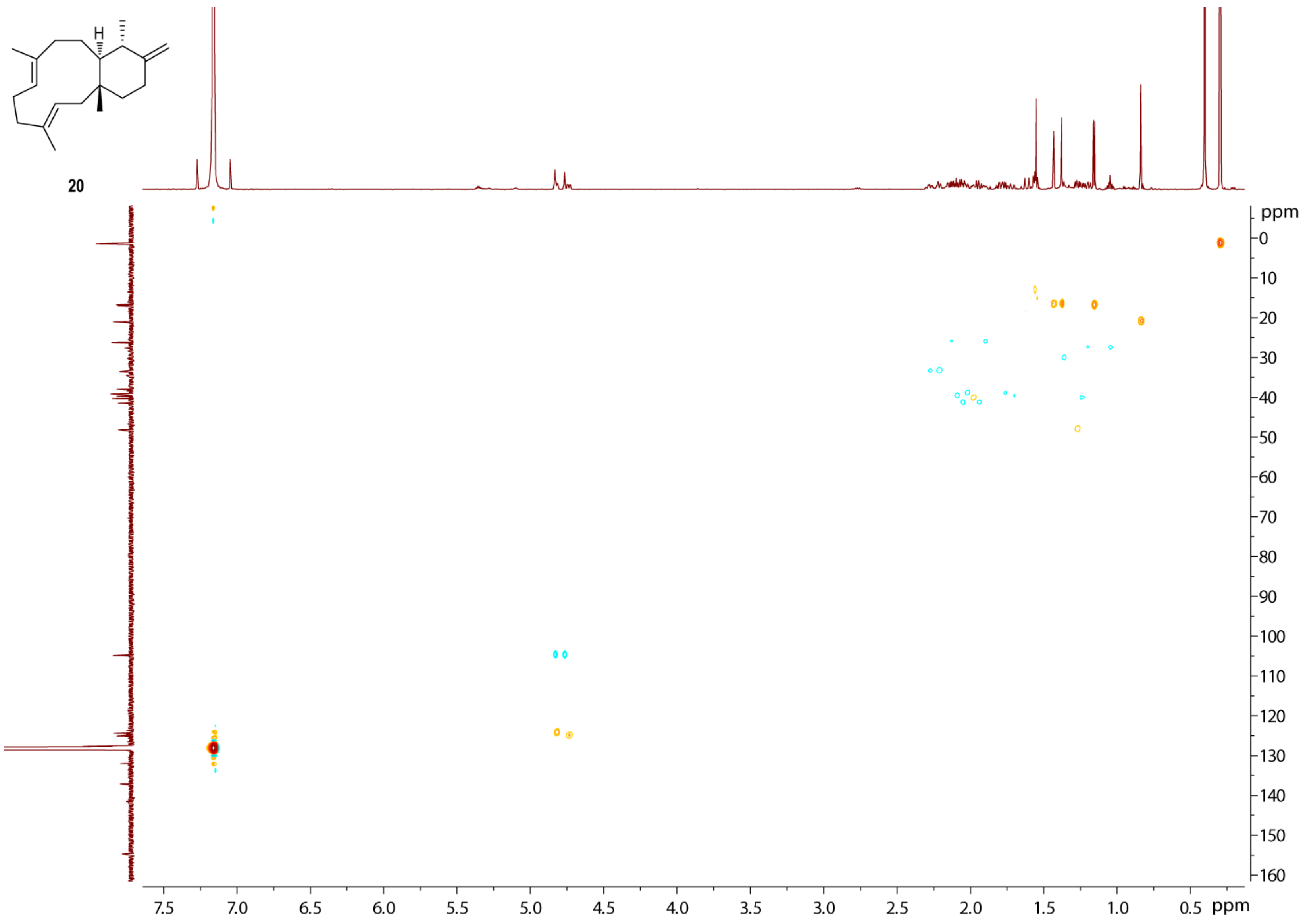

Figure S38. HSQC spectrum of **20** (C_6D_6).

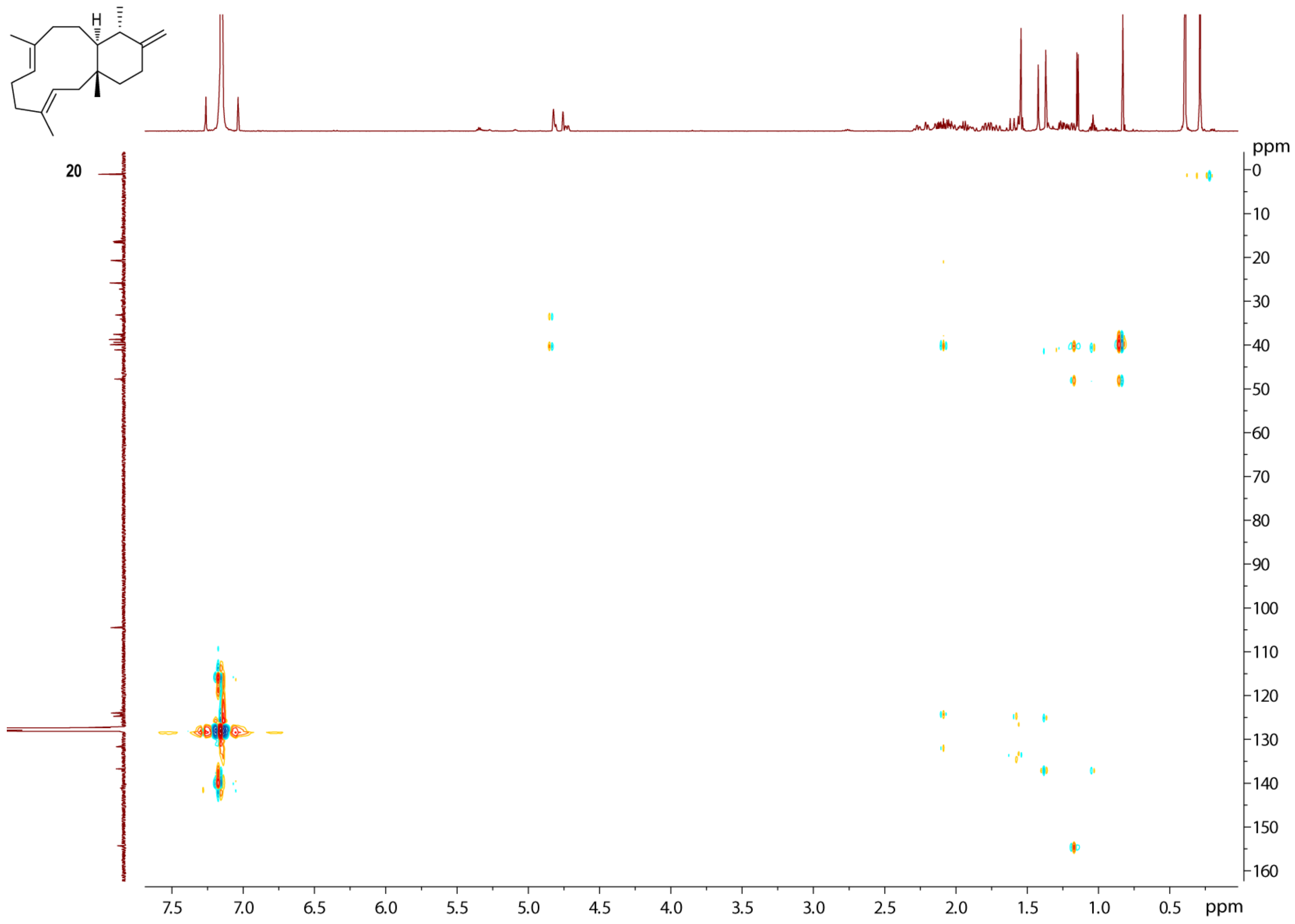

Figure S39. HMBC spectrum of **20** (C_6D_6).

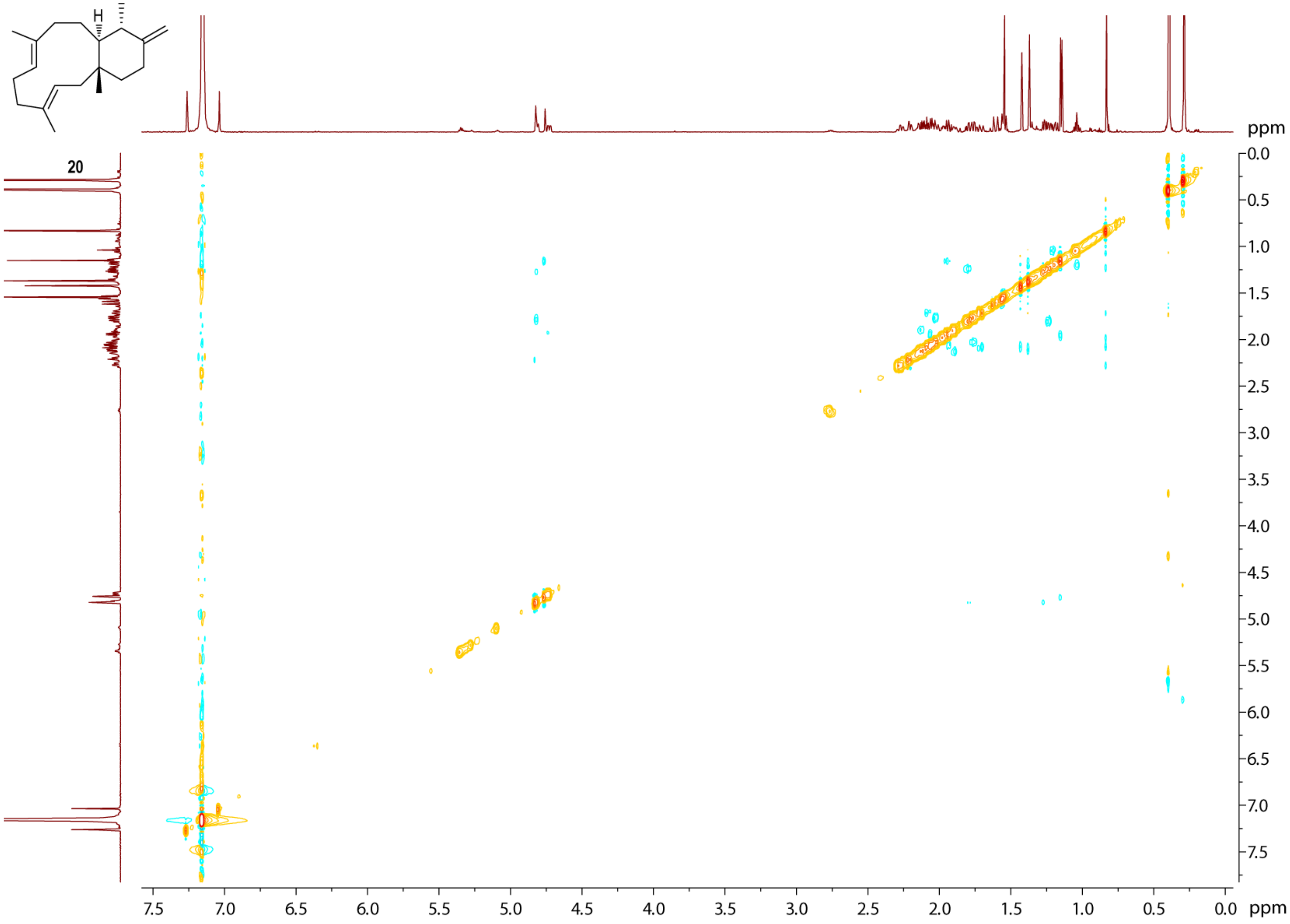

Figure S40. NOESY spectrum of **20** (C_6D_6).

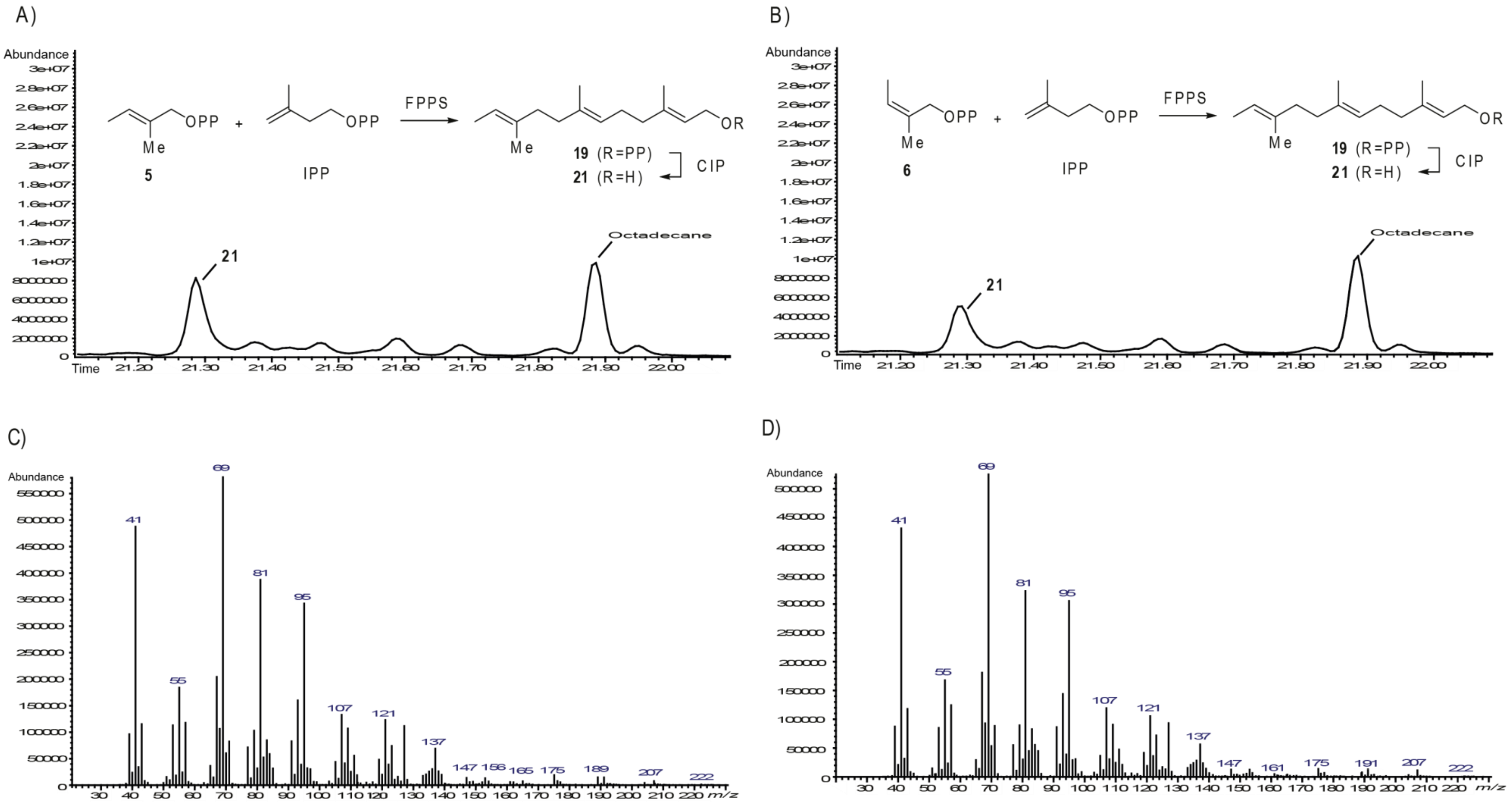

Figure S41. The total ion chromatogram of products from incubation of both DMAPP analogs A) **5** and B) **6** with IPP and FPPS and dephosphorylation with CIP. EI mass spectra of the compound eluting at 21.29 min obtained from DMAPP analogs C) **5** and D) **6**. The identical retention times and mass spectra indicate that from both DMAPP analogs **5** and **6** the same FPP analog **19** is obtained, which is explainable by a configurational isomerisation during the elongation of **6** with IPP. Octadecane was added as an internal standard.

Incubation experiments with isotopically labelled substrates

Isotopic labelling experiments were performed with amounts of ca. 1 mg labelled diphosphate dissolved in substrate buffer (1 mL), incubation buffer (5 mL), enzyme elution fractions (1 mL of terpene synthase solution and 0.5 mL for each additional enzyme (FPPS, GGPPS)) and binding buffer (to 10 mL total volume) with the substrates and enzyme preparations as listed in Table S6. After incubation with shaking at 28 °C for 3 h the products were extracted with C_6D_6 (650 μL and 300 μL) and analysed by NMR and GC/MS.

Table S6. Enzyme incubation experiments with isotopically labelled substrates.

substrates	enzymes	shown in
**2** + (*Z*)-(4-^{13}C,4-^{2}H)IPP	AbVS, GGPPS, FPPS	Figure S42
**2** + (*E*)-(4-^{13}C,4-^{2}H)IPP	AbVS, GGPPS, FPPS	Figure S42
**2** + (*R*)-(1-^{13}C,1-^{2}H)IPP	AbVS, GGPPS, FPPS	Figure S43
**2** + (*S*)-(1-^{13}C,1-^{2}H)IPP	AbVS, GGPPS, FPPS	Figure S43
**3** + (*Z*)-(4-^{13}C,4-^{2}H)IPP	AbVS, GGPPS, FPPS	Figure S44
**3** + (*E*)-(4-^{13}C,4-^{2}H)IPP	AbVS, GGPPS, FPPS	Figure S44
**3** + (*R*)-(1-^{13}C,1-^{2}H)IPP	AbVS, GGPPS, FPPS	Figure S45
**3** + (*S*)-(1-^{13}C,1-^{2}H)IPP	AbVS, GGPPS, FPPS	Figure S45
**4** + (*Z*)-(4-^{13}C,4-^{2}H)IPP	AbVS, GGPPS, FPPS	Figure S46, S48
**4** + (*E*)-(4-^{13}C,4-^{2}H)IPP	AbVS, GGPPS, FPPS	Figure S46, S48
**4** + (*R*)-(1-^{13}C,1-^{2}H)IPP	AbVS, GGPPS, FPPS	Figure S47, S49
**4** + (*S*)-(1-^{13}C,1-^{2}H)IPP	AbVS, GGPPS, FPPS	Figure S47, S49
**5** + (*Z*)-(4-^{13}C,4-^{2}H)IPP	AbVS, GGPPS, FPPS	Figure S50
**5** + (*E*)-(4-^{13}C,4-^{2}H)IPP	AbVS, GGPPS, FPPS	Figure S50
**5** + (*R*)-(1-^{13}C,1-^{2}H)IPP	AbVS, GGPPS, FPPS	Figure S51
**5** + (*S*)-(1-^{13}C,1-^{2}H)IPP	AbVS, GGPPS, FPPS	Figure S51

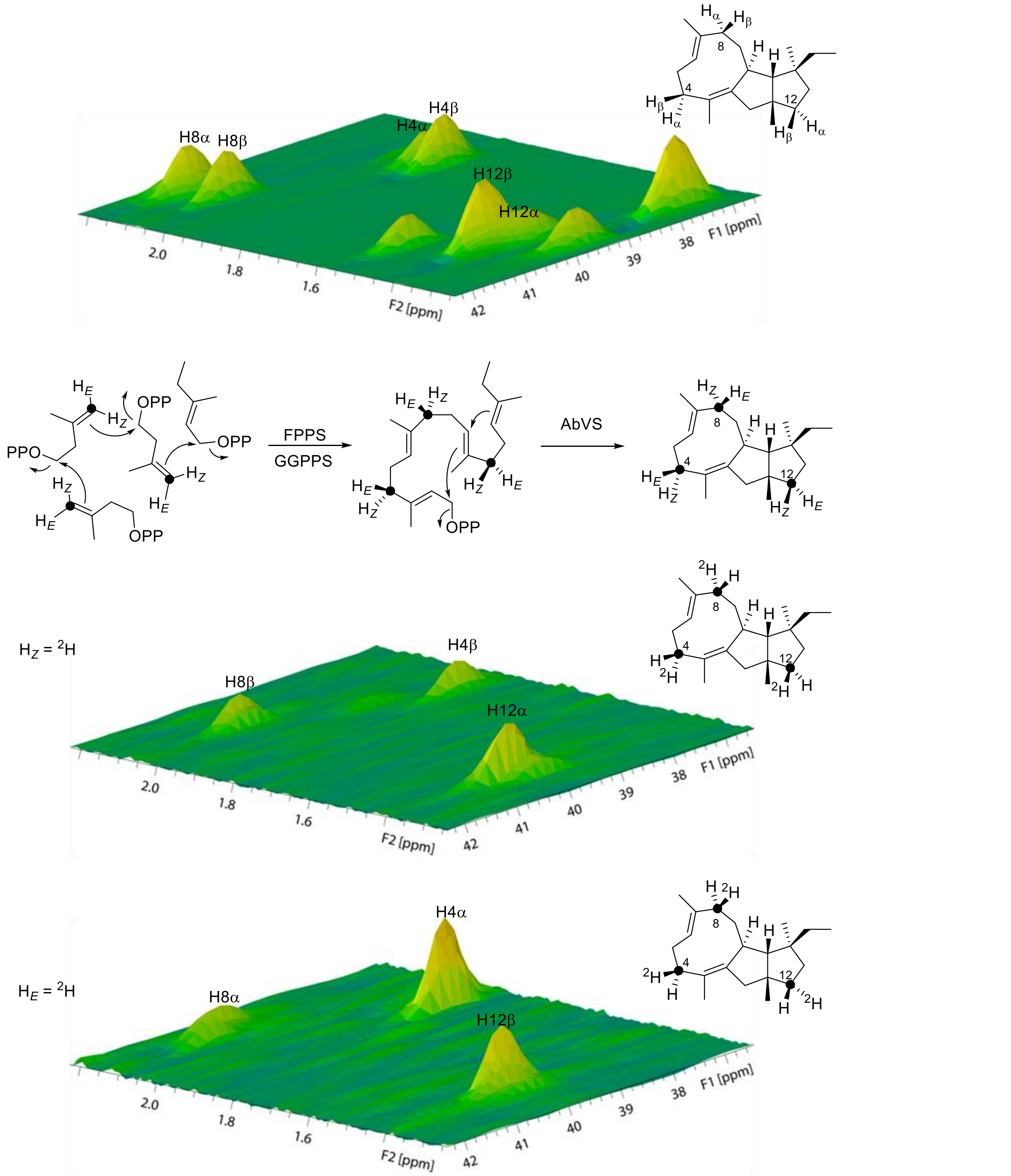

Figure S42. Determination of the absolute configuration of **8** by isotopic labelling experiments. Partial HSQC spectra of unlabelled **8** (top) in comparison to extracts from incubation experiments using AbVS, FPPS, GGPPS and (*Z*)-(4-^{13}C,4-^{2}H)IPP (middle) or (*E*)-(4-^{13}C,4-^{2}H)IPP (bottom) showing the incorporation of deuterium into the expected positions for the shown absolute configuration of **8**. Black dots represent ^{13}C-labelled atoms.

Because of the known stereochemical course for the formation of GGPP from DMAPP and IPP the configurations at the deuterated carbons from (*Z*)-(4-^{13}C,4-^{2}H)IPP and (*E*)-(4-^{13}C,4-^{2}H)IPP are known. For each experiment, only one signal for the remaining hydrogen at these carbons is visible in the HSQC spectra. Using these stereochemical anchors and together with the NOESY based assignment of diastereotopic hadrogens in the CH_2 groups of unlabelled **8** (Table S1) the relative orientation of the naturally present stereogenic centers is known, which allows to assign the absolute configuration of **8**.

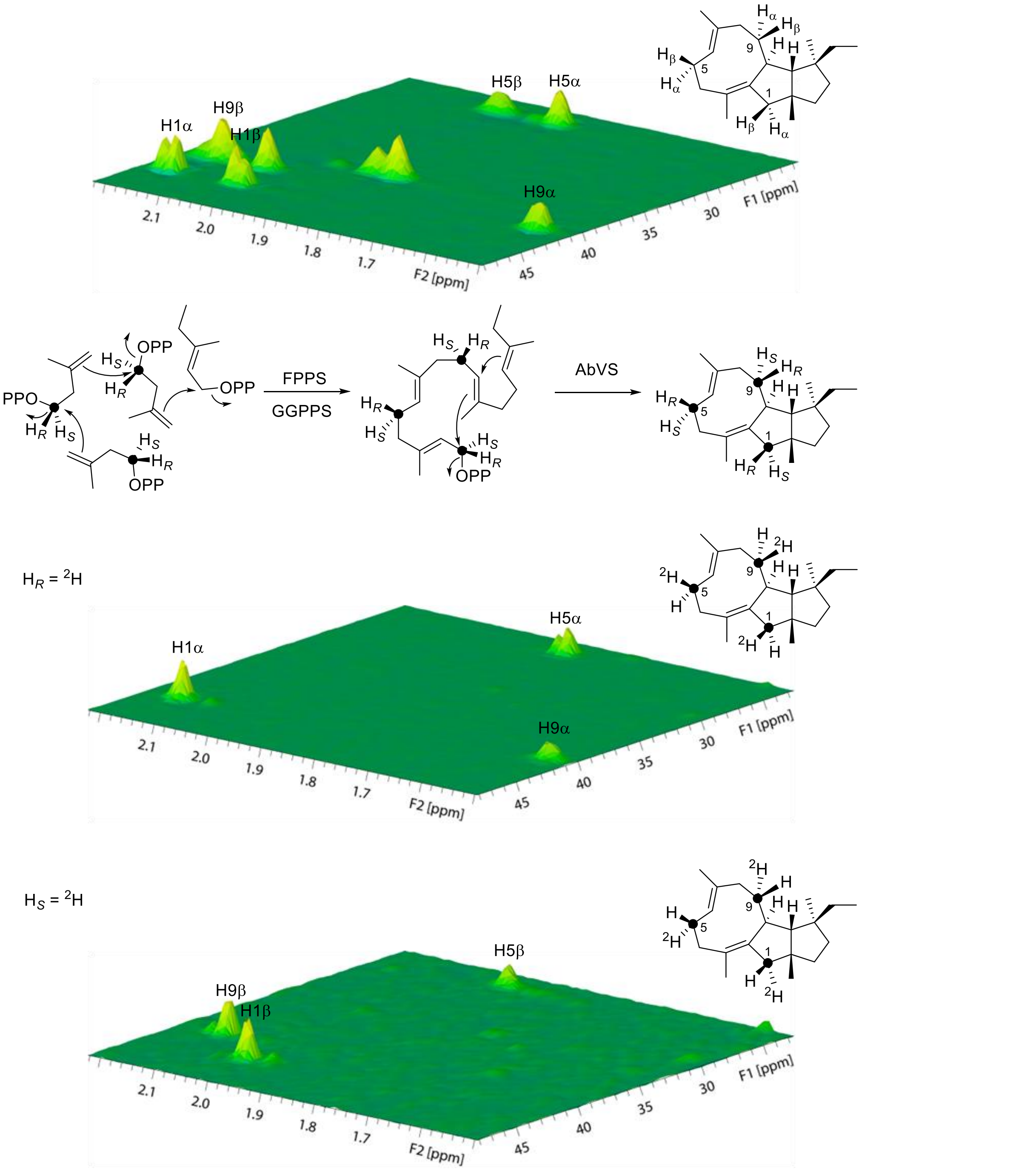

Figure S43. Determination of the absolute configuration of **8** by isotopic labelling experiments. Partial HSQC spectra of unlabelled **8** (top) in comparison to extracts from incubation experiments using AbVS, FPPS, GGPPS and (*R*)-(1-^{13}C,1-^{2}H)IPP (middle) or (*S*)-(1-^{13}C,1-^{2}H)IPP (bottom) showing the incorporation of deuterium into the expected positions for the shown absolute configuration of **8**. Black dots represent ^{13}C-labelled atoms.

Because of the known stereochemical course for the formation of GGPP from DMAPP and IPP the configurations at the deuterated carbons from (*R*)-(1-^{13}C,1-^{2}H)IPP and (*S*)-(1-^{13}C,1-^{2}H)IPP are known. For each experiment, only one signal for the remaining hydrogen at these carbons is visible in the HSQC spectra. Using these stereochemical anchors and together with the NOESY based assignment of diastereotopic hadrogens in the CH_2 groups of unlabelled **8** (Table S1) the relative orientation of the naturally present stereogenic centers is known, which allows to assign the absolute configuration of **8**.

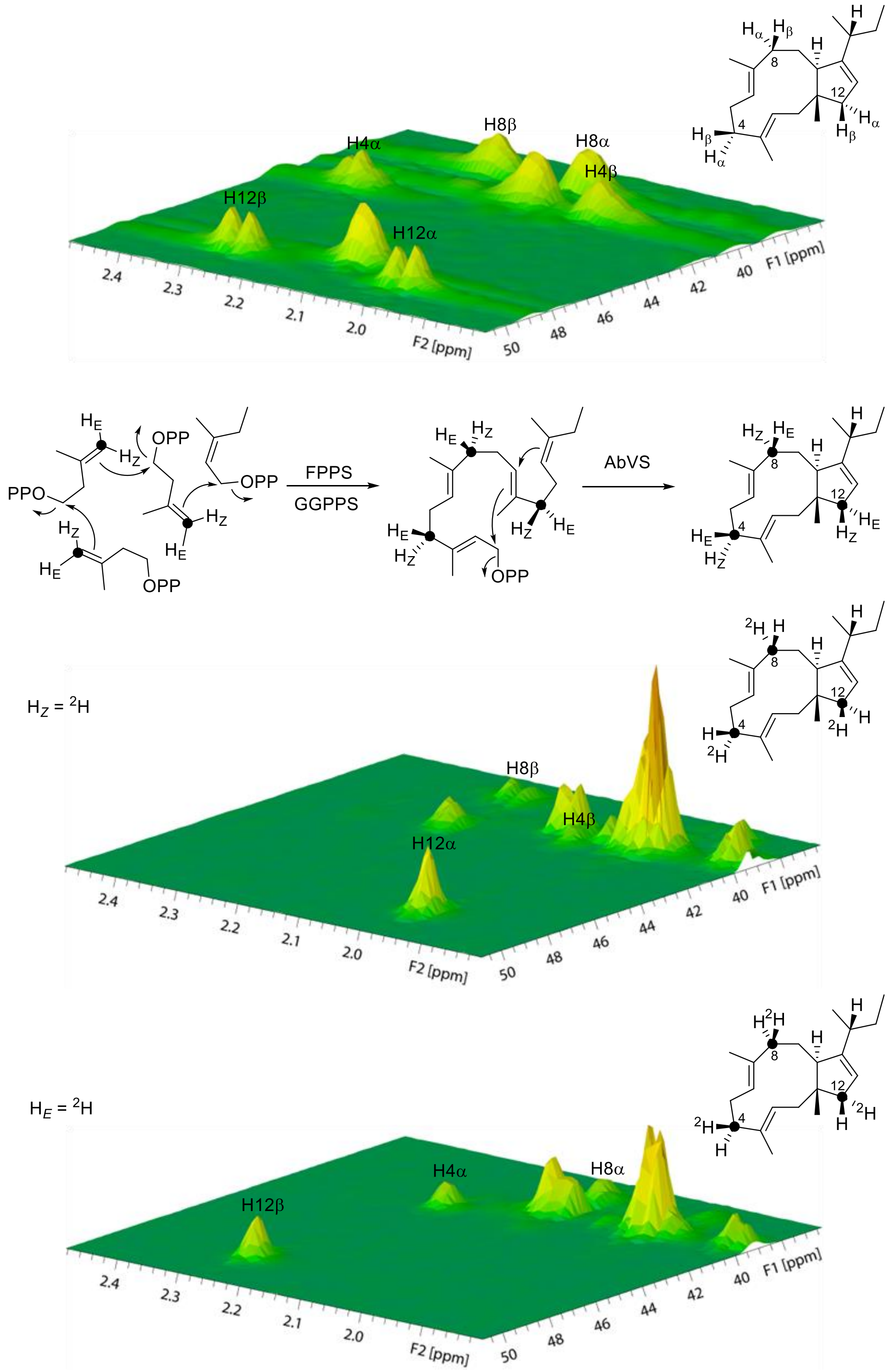

Figure S44. Determination of the absolute configuration of **10** by isotopic labelling experiments. Partial HSQC spectra of unlabelled **10** (top) in comparison to extracts from incubation experiments using AbVS, FPPS, GGPPS and (*Z*)-(4-^{13}C,4-^{2}H)IPP (middle) or (*E*)-(4-^{13}C,4-^{2}H)IPP (bottom) showing the incorporation of deuterium into the expected positions for the shown absolute configuration of **10**. Black dots represent ^{13}C-labelled atoms.

Because of the known stereochemical course for the formation of GGPP from DMAPP and IPP the configurations at the deuterated carbons from (*Z*)-(4-^{13}C,4-^{2}H)IPP and (*E*)-(4-^{13}C,4-^{2}H)IPP are known. For each experiment, only one signal for the remaining hydrogen at these carbons is visible in the HSQC spectra. Using these stereochemical anchors and together with the NOESY based assignment of diastereotopic hadrogens in the CH_2 groups of unlabelled **10** (Table S2) the relative orientation of the naturally present stereogenic centers is known, which allows to assign the absolute configuration of **10**.

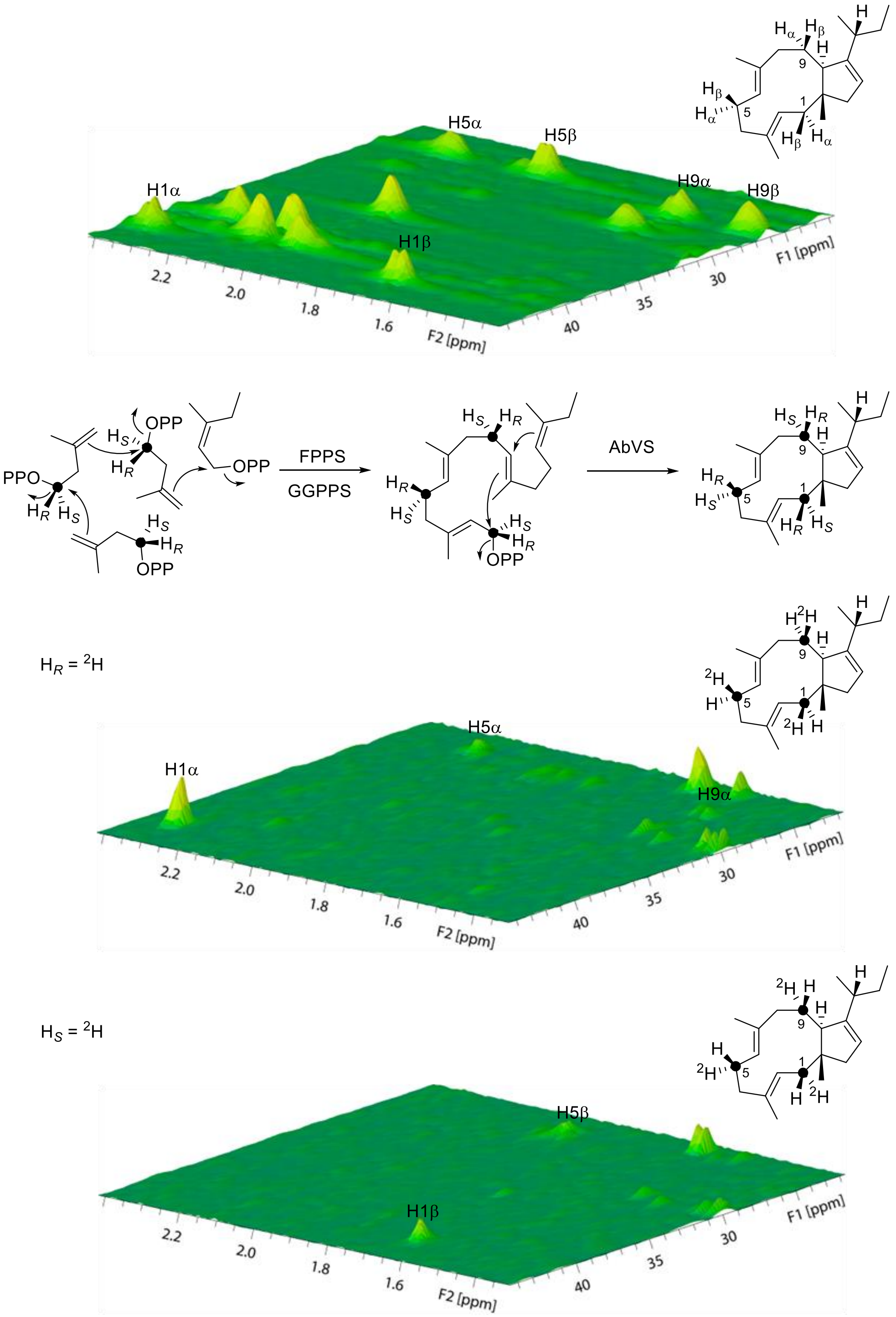

Figure S45. Determination of the absolute configuration of **10** by isotopic labelling experiments. Partial HSQC spectra of unlabelled **10** (top) in comparison to extracts from incubation experiments using AbVS, FPPS, GGPPS and (*R*)-(1-^{13}C,1-^{2}H)IPP (middle) or (*S*)-(1-^{13}C,1-^{2}H)IPP (bottom) showing the incorporation of deuterium into the expected positions for the shown absolute configuration of **10**. Black dots represent ^{13}C-labelled atoms.

Because of the known stereochemical course for the formation of GGPP from DMAPP and IPP the configurations at the deuterated carbons from (*R*)-(1-^{13}C,1-^{2}H)IPP and (*S*)-(1-^{13}C,1-^{2}H)IPP are known. For each experiment, only one signal for the remaining hydrogen at these carbons is visible in the HSQC spectra. Using these stereochemical anchors and together with the NOESY based assignment of diastereotopic hadrogens in the CH_2 groups of unlabelled **10** (Table S2) the relative orientation of the naturally present stereogenic centers is known, which allows to assign the absolute configuration of **10**.

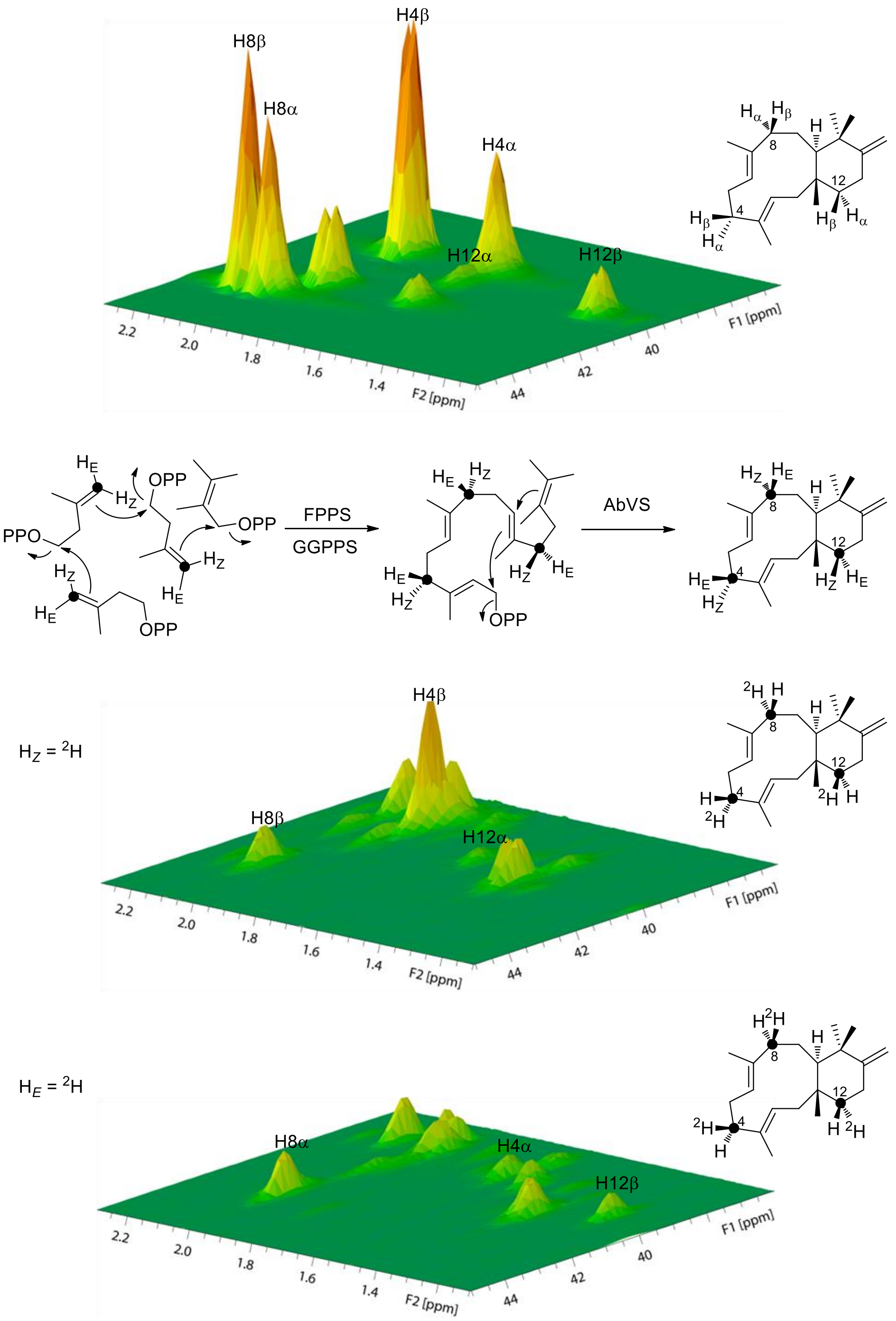

Figure S46. Determination of the absolute configuration of **13** by isotopic labelling experiments. Partial HSQC spectra of unlabelled **13** (top) in comparison to extracts from incubation experiments using AbVS, FPPS, GGPPS and (*Z*)-(4-^{13}C,4-^{2}H)IPP (middle) or (*E*)-(4-^{13}C,4-^{2}H)IPP (bottom) showing the incorporation of deuterium into the expected positions for the shown absolute configuration of **13**. Black dots represent ^{13}C-labelled atoms.

Because of the known stereochemical course for the formation of GGPP from DMAPP and IPP the configurations at the deuterated carbons from (*Z*)-(4-^{13}C,4-^{2}H)IPP and (*E*)-(4-^{13}C,4-^{2}H)IPP are known. For each experiment, only one signal for the remaining hydrogen at these carbons is visible in the HSQC spectra. Using these stereochemical anchors and together with the NOESY based assignment of diastereotopic hadrogens in the CH_2 groups of unlabelled **13** (Table S3) the relative orientation of the naturally present stereogenic centers is known, which allows to assign the absolute configuration of **13**.

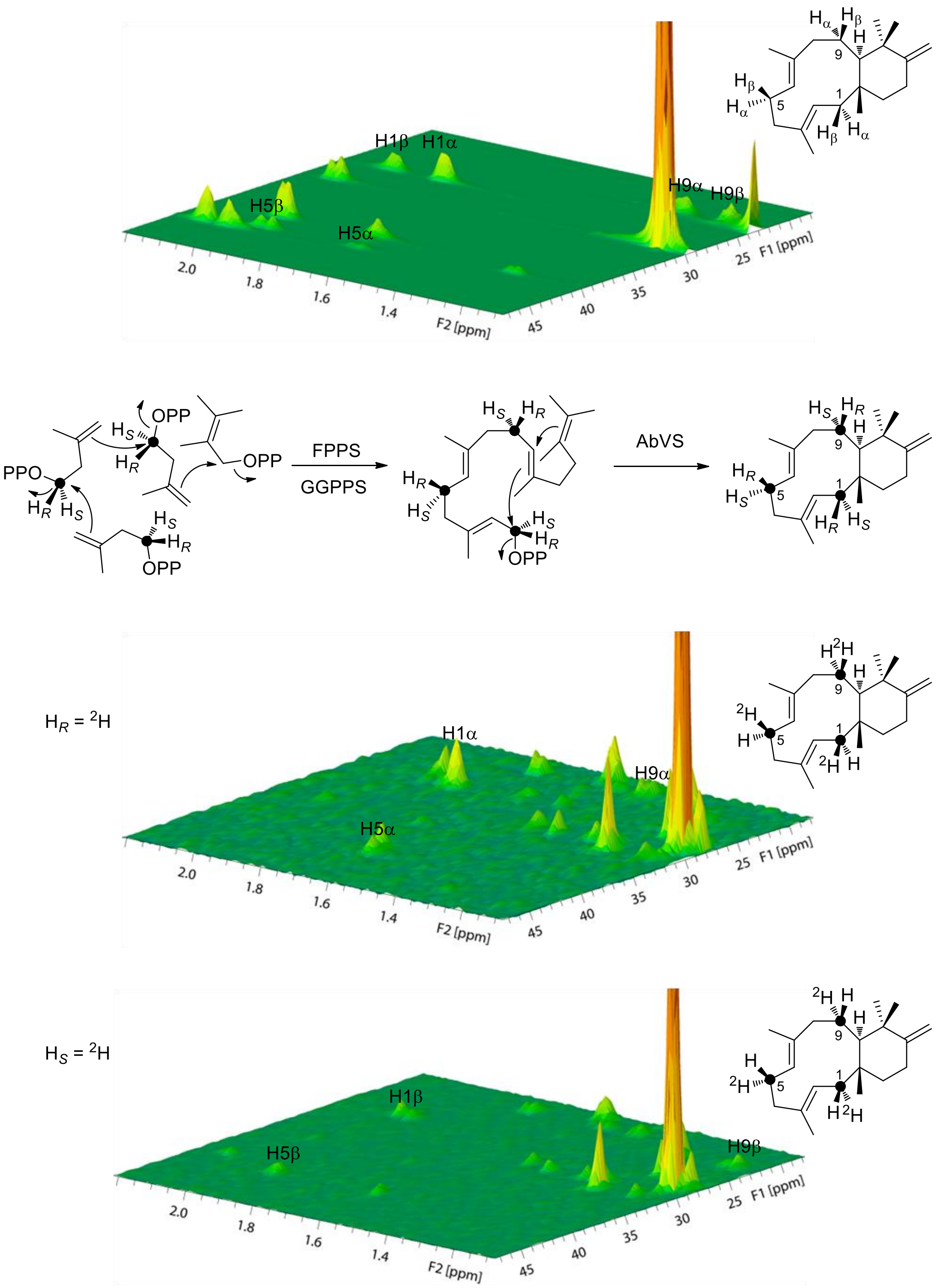

Figure S47. Determination of the absolute configuration of **13** by isotopic labelling experiments. Partial HSQC spectra of unlabelled **13** (top) in comparison to extracts from incubation experiments using AbVS, FPPS, GGPPS and (*R*)-(1-^{13}C,1-^{2}H)IPP (middle) or (*S*)-(1-^{13}C,1-^{2}H)IPP (bottom) showing the incorporation of deuterium into the expected positions for the shown absolute configuration of **13**. Black dots represent ^{13}C-labelled atoms.

Because of the known stereochemical course for the formation of GGPP from DMAPP and IPP the configurations at the deuterated carbons from (*R*)-(1-^{13}C,1-^{2}H)IPP and (*S*)-(1-^{13}C,1-^{2}H)IPP are known. For each experiment, only one signal for the remaining hydrogen at these carbons is visible in the HSQC spectra. Using these stereochemical anchors and together with the NOESY based assignment of diastereotopic hadrogens in the CH_2 groups of unlabelled **13** (Table S3) the relative orientation of the naturally present stereogenic centers is known, which allows to assign the absolute configuration of **13**.

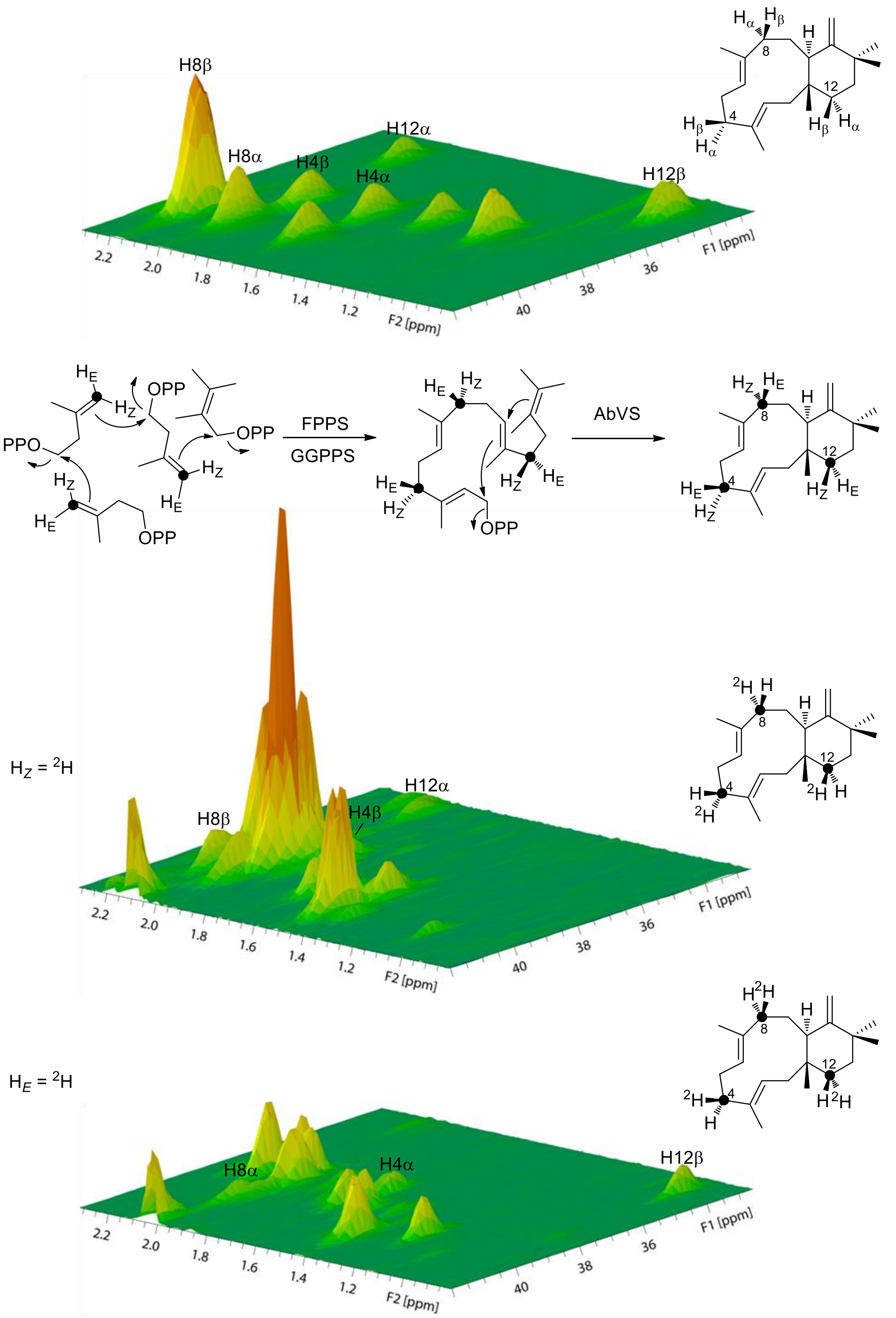

Figure S48. Determination of the absolute configuration of **14** by isotopic labeling experiments. Partial HSQC spectra of unlabelled **14** (top) in comparison to extracts from incubation experiments using AbVS, FPPS, GGPPS and (*Z*)-(4-^{13}C,4-^{2}H)IPP (middle) or (*E*)-(4-^{13}C,4-^{2}H)IPP (bottom) showing the incorporation of deuterium into the expected positions for the shown absolute configuration of **14**. Black dots represent ^{13}C-labelled atoms.

Because of the known stereochemical course for the formation of GGPP from DMAPP and IPP the configurations at the deuterated carbons from (*Z*)-(4-^{13}C,4-^{2}H)IPP and (*E*)-(4-^{13}C,4-^{2}H)IPP are known. For each experiment, only one signal for the remaining hydrogen at these carbons is visible in the HSQC spectra. Using these stereochemical anchors and together with the NOESY based assignment of diastereotopic hadrogens in the CH_2 groups of unlabelled **14** (Table S4) the relative orientation of the naturally present stereogenic centers is known, which allows to assign the absolute configuration of **14**.

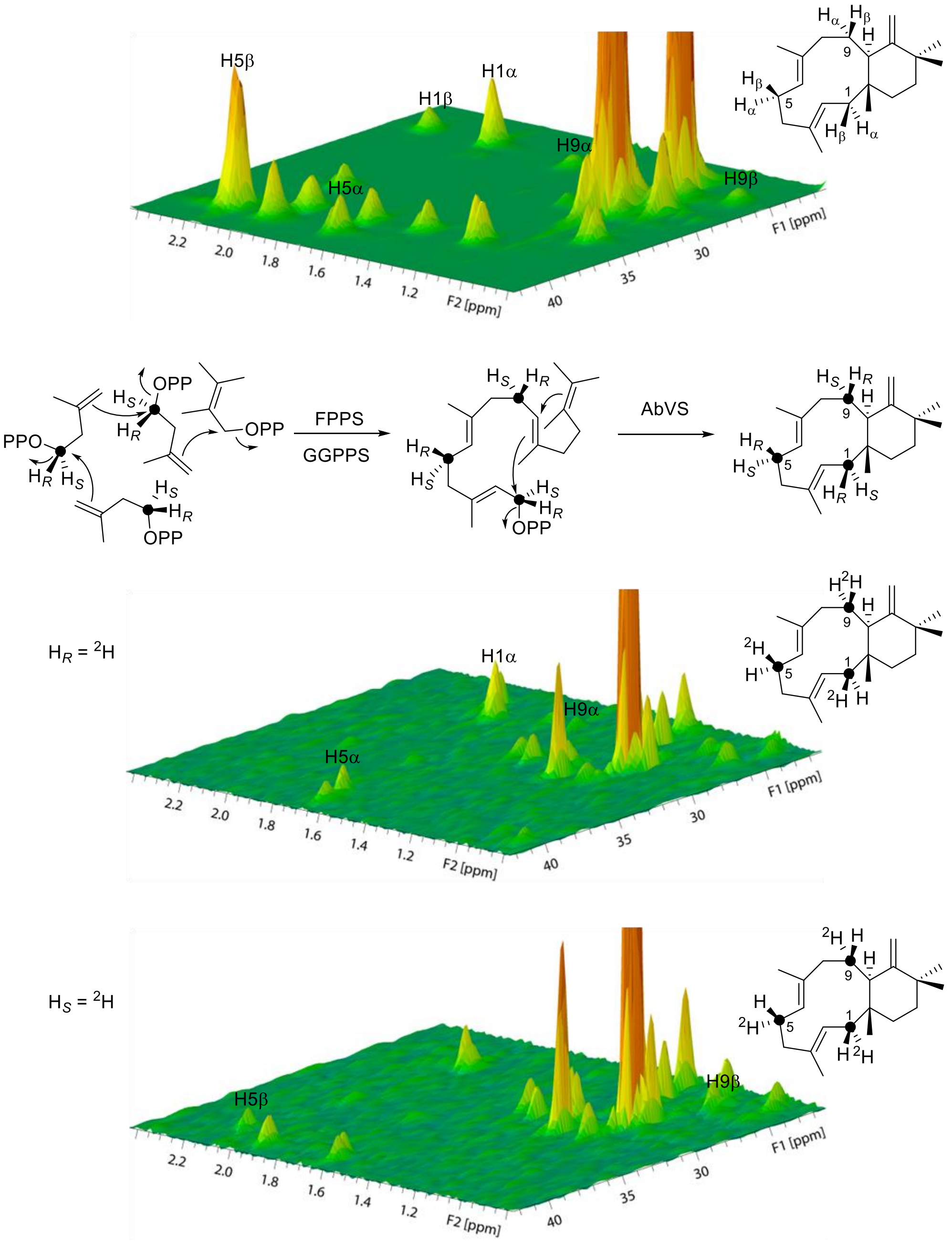

Figure S49. Determination of the absolute configuration of **14** by isotopic labelling experiments. Partial HSQC spectra of unlabelled **14** (top) in comparison to extracts from incubation experiments using AbVS, FPPS, GGPPS and (*R*)-(1-^{13}C,1-^{2}H)IPP (middle) or (*S*)-(1-^{13}C,1-^{2}H)IPP (bottom) showing the incorporation of deuterium into the expected positions for the shown absolute configuration of **14**. Black dots represent ^{13}C-labelled atoms.

Because of the known stereochemical course for the formation of GGPP from DMAPP and IPP the configurations at the deuterated carbons from (*R*)-(1-^{13}C,1-^{2}H)IPP and (*S*)-(1-^{13}C,1-^{2}H)IPP are known. For each experiment, only one signal for the remaining hydrogen at these carbons is visible in the HSQC spectra. Using these stereochemical anchors and together with the NOESY based assignment of diastereotopic hadrogens in the CH_2 groups of unlabelled **14** (Table S4) the relative orientation of the naturally present stereogenic centers is known, which allows to assign the absolute configuration of **14**.

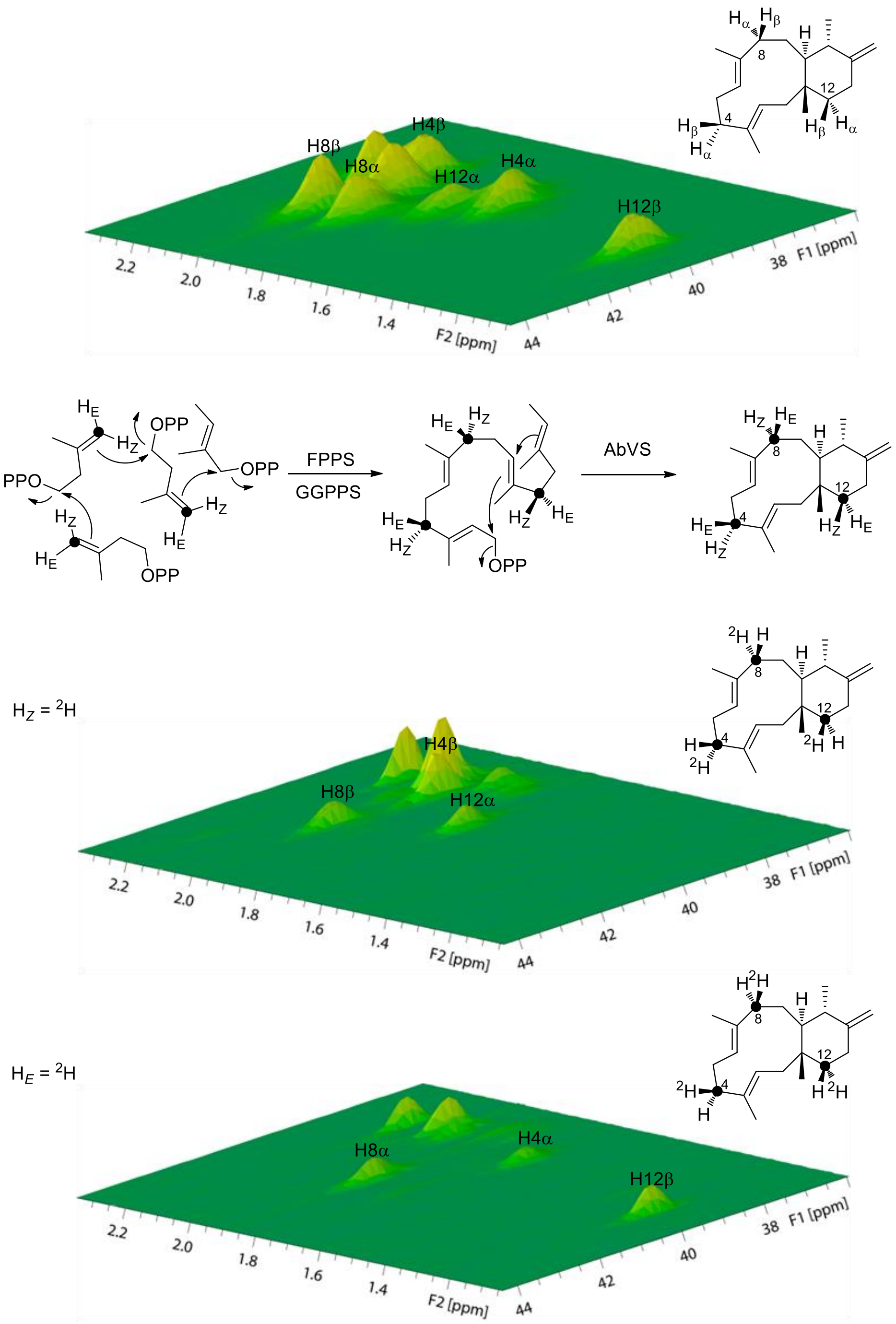

Figure S50. Determination of the absolute configuration of **20** by isotopic labelling experiments. Partial HSQC spectra of unlabelled **20** (top) in comparison to extracts from incubation experiments using AbVS, FPPS, GGPPS and (*Z*)-(4-^{13}C,4-^{2}H)IPP (middle) or (*E*)-(4-^{13}C,4-^{2}H)IPP (bottom) showing the incorporation of deuterium into the expected positions for the shown absolute configuration of **20**. Black dots represent ^{13}C-labelled atoms.

Because of the known stereochemical course for the formation of GGPP from DMAPP and IPP the configurations at the deuterated carbons from (*Z*)-(4-^{13}C,4-^{2}H)IPP and (*E*)-(4-^{13}C,4-^{2}H)IPP are known. For each experiment, only one signal for the remaining hydrogen at these carbons is visible in the HSQC spectra. Using these stereochemical anchors and together with the NOESY based assignment of diastereotopic hadrogens in the CH_2 groups of unlabelled **20** (Table S5) the relative orientation of the naturally present stereogenic centers is known, which allows to assign the absolute configuration of **20**.

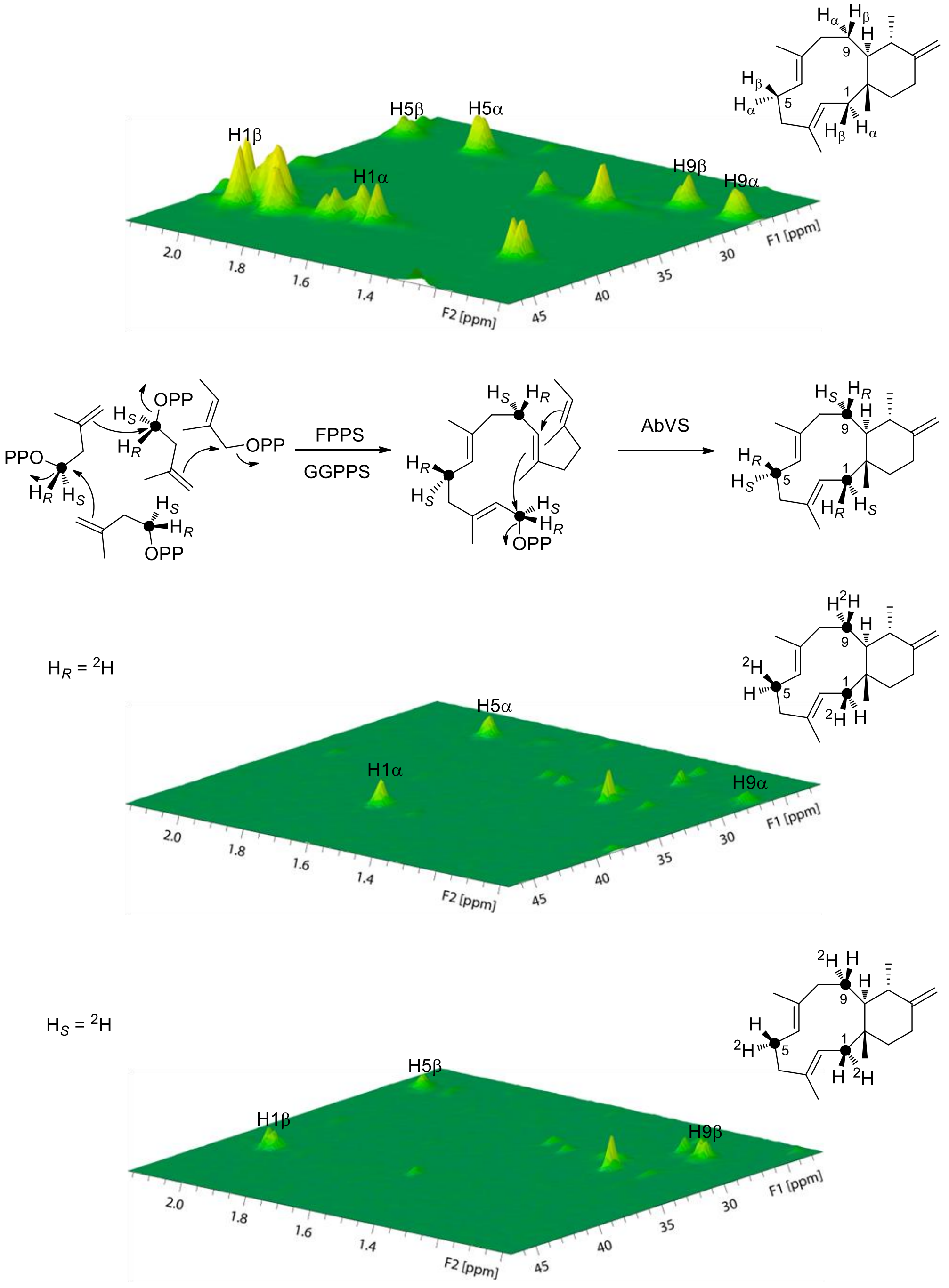

Figure S51. Determination of the absolute configuration of **20** by isotopic labelling experiments. Partial HSQC spectra of unlabelled **20** (top) in comparison to extracts from incubation experiments using AbVS, FPPS, GGPPS and (*R*)-(1-^{13}C,1-^{2}H)IPP (middle) or (*S*)-(1-^{13}C,1-^{2}H)IPP (bottom) showing the incorporation of deuterium into the expected positions for the shown absolute configuration of **20**. Black dots represent ^{13}C-labelled atoms.

Because of the known stereochemical course for the formation of GGPP from DMAPP and IPP the configurations at the deuterated carbons from (*R*)-(1-^{13}C,1-^{2}H)IPP and (*S*)-(1-^{13}C,1-^{2}H)IPP are known. For each experiment, only one signal for the remaining hydrogen at these carbons is visible in the HSQC spectra. Using these stereochemical anchors and together with the NOESY based assignment of diastereotopic hadrogens in the CH_2 groups of unlabelled **20** (Table S5) the relative orientation of the naturally present stereogenic centers is known, which allows to assign the absolute configuration of **20**.

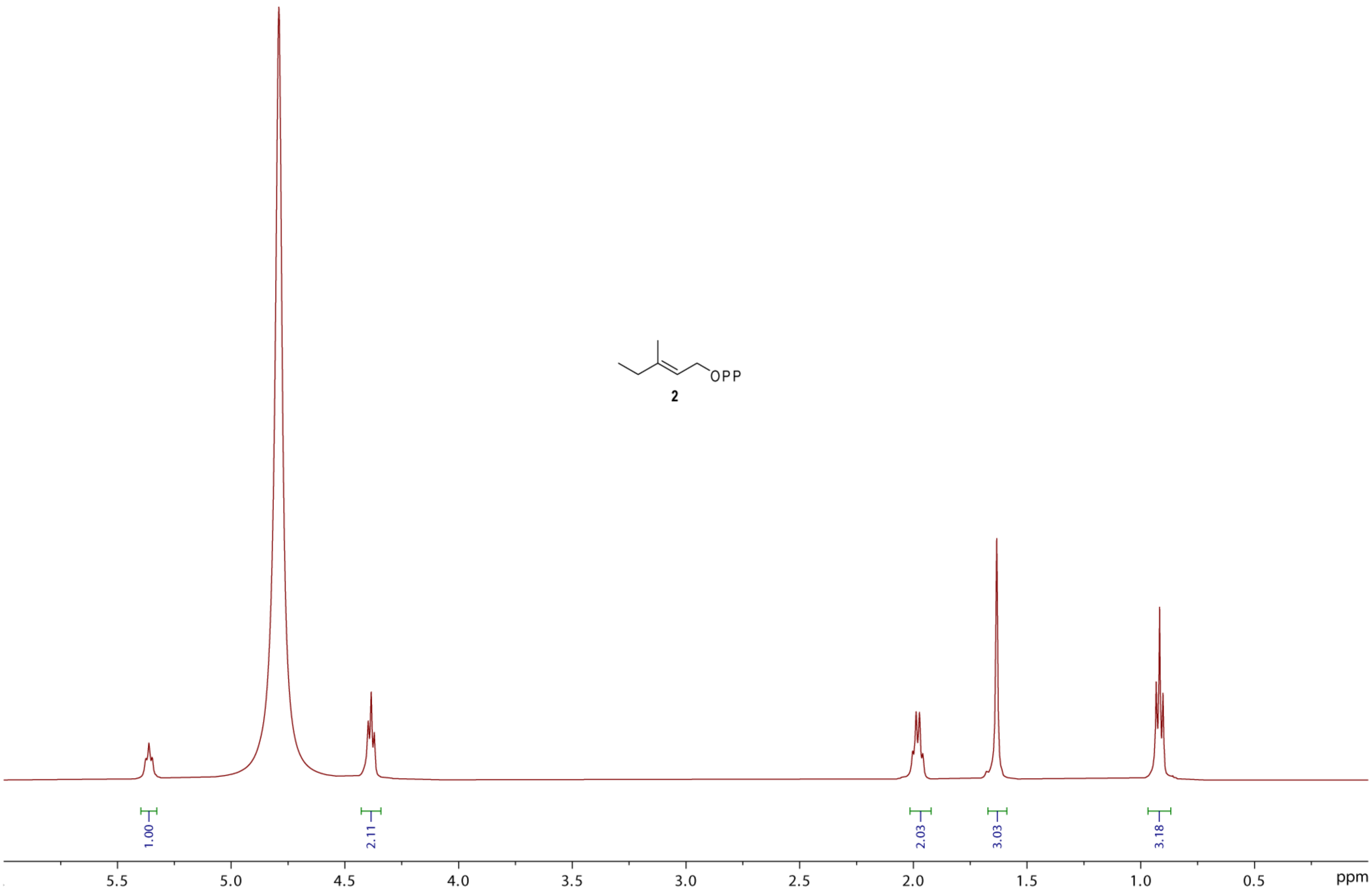

Figure S52. ^{1}H-NMR spectrum of **2** (500 MHz, D_2O).

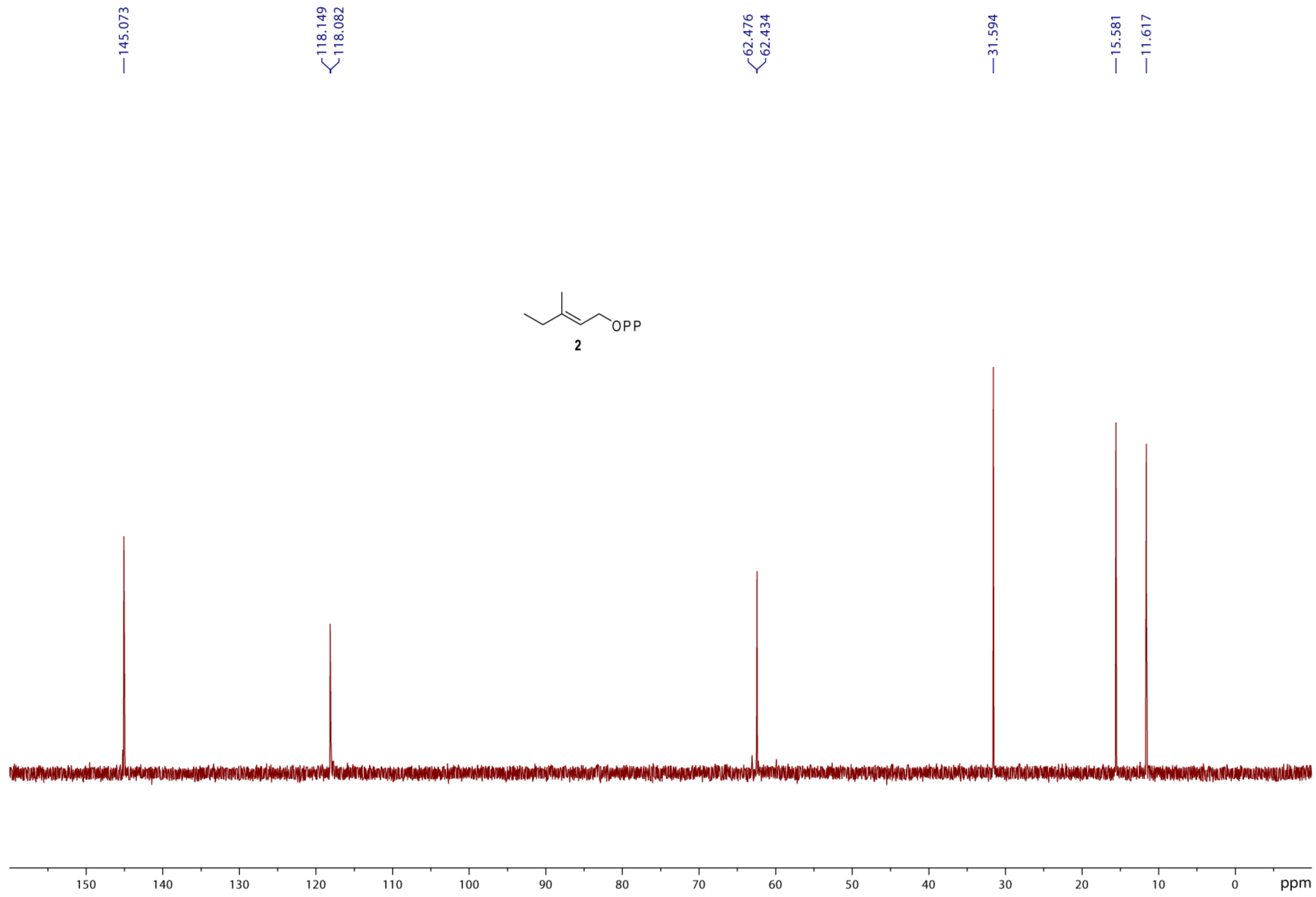

Figure S53. ^{13}C-NMR spectrum of **2** (126 MHz, D_2O).

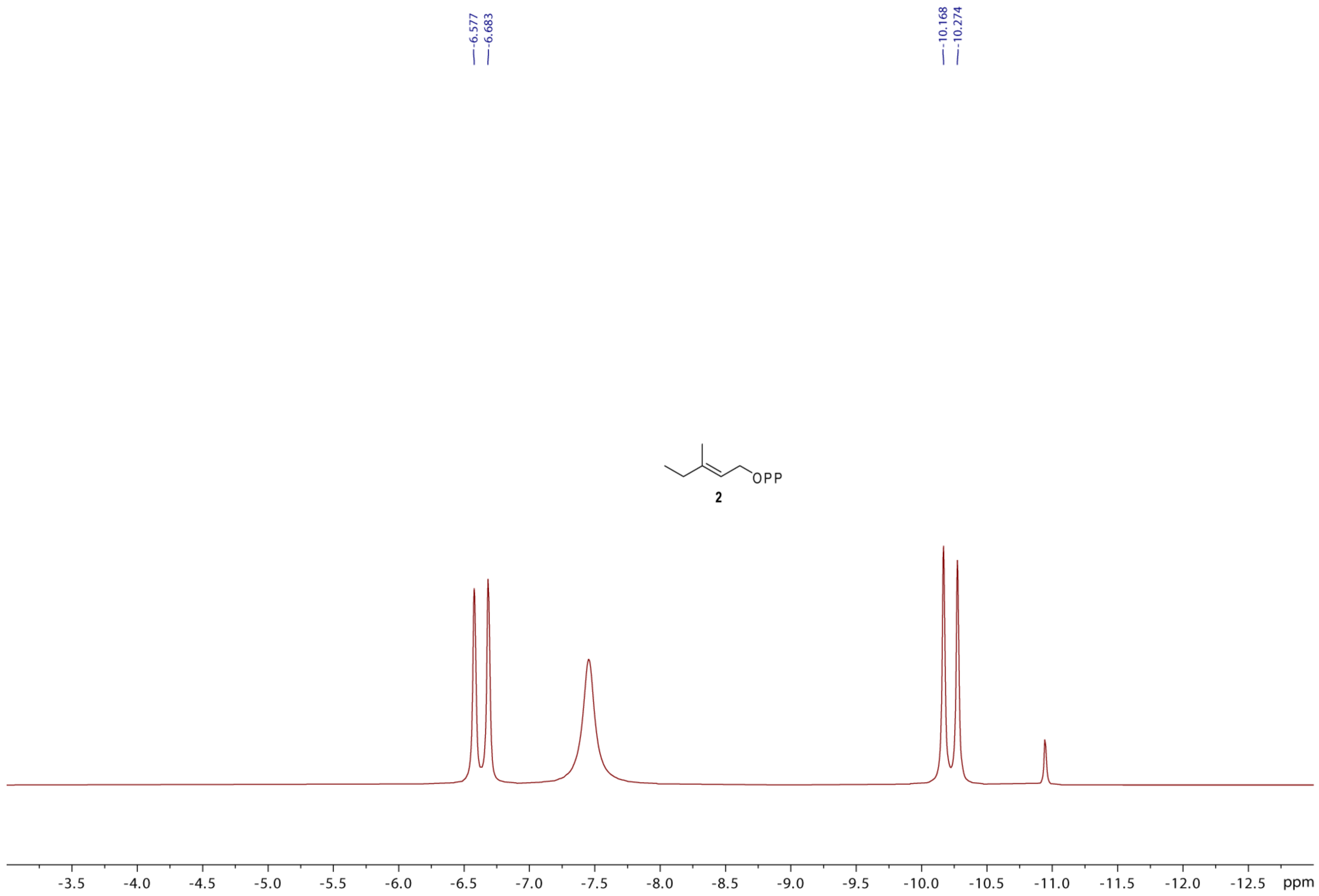

Figure S54. ^{31}P-NMR of **2** (202 MHz, D_2O).

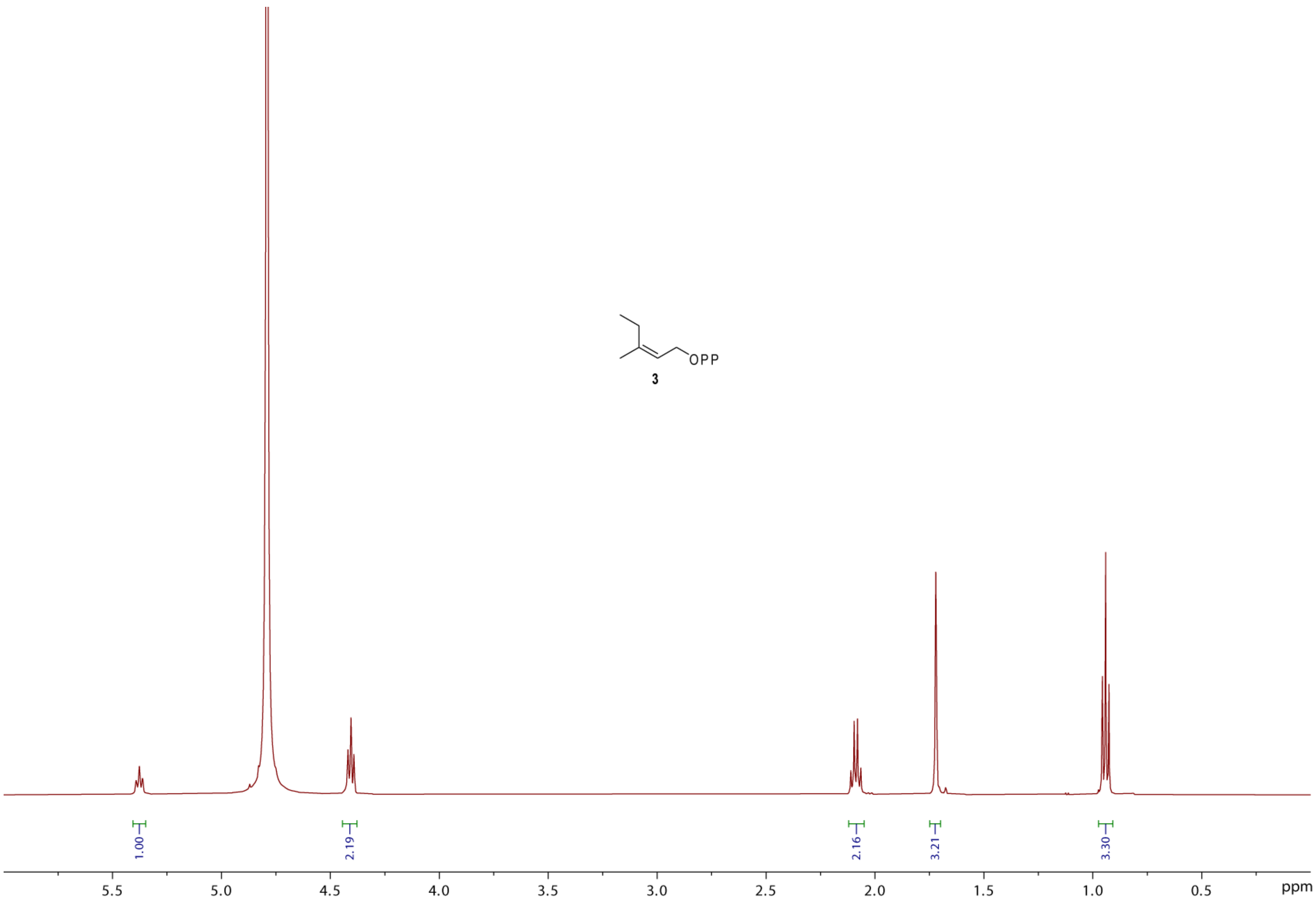

Figure S55. ^{1}H-NMR spectrum of **3** (500 MHz, D_2O).

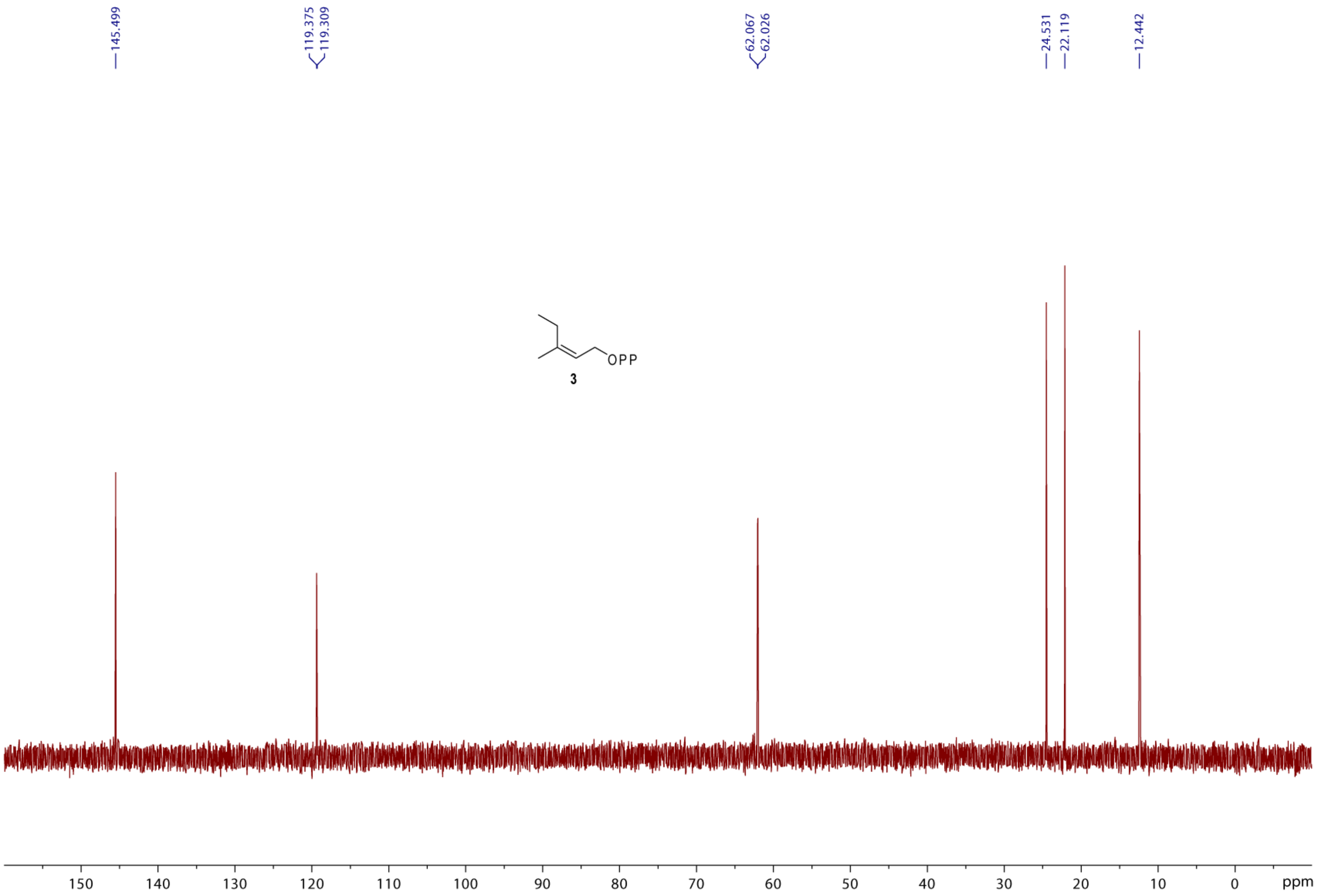

Figure S56. ^{13}C-NMR spectrum of **3** (126 MHz, D_2O).

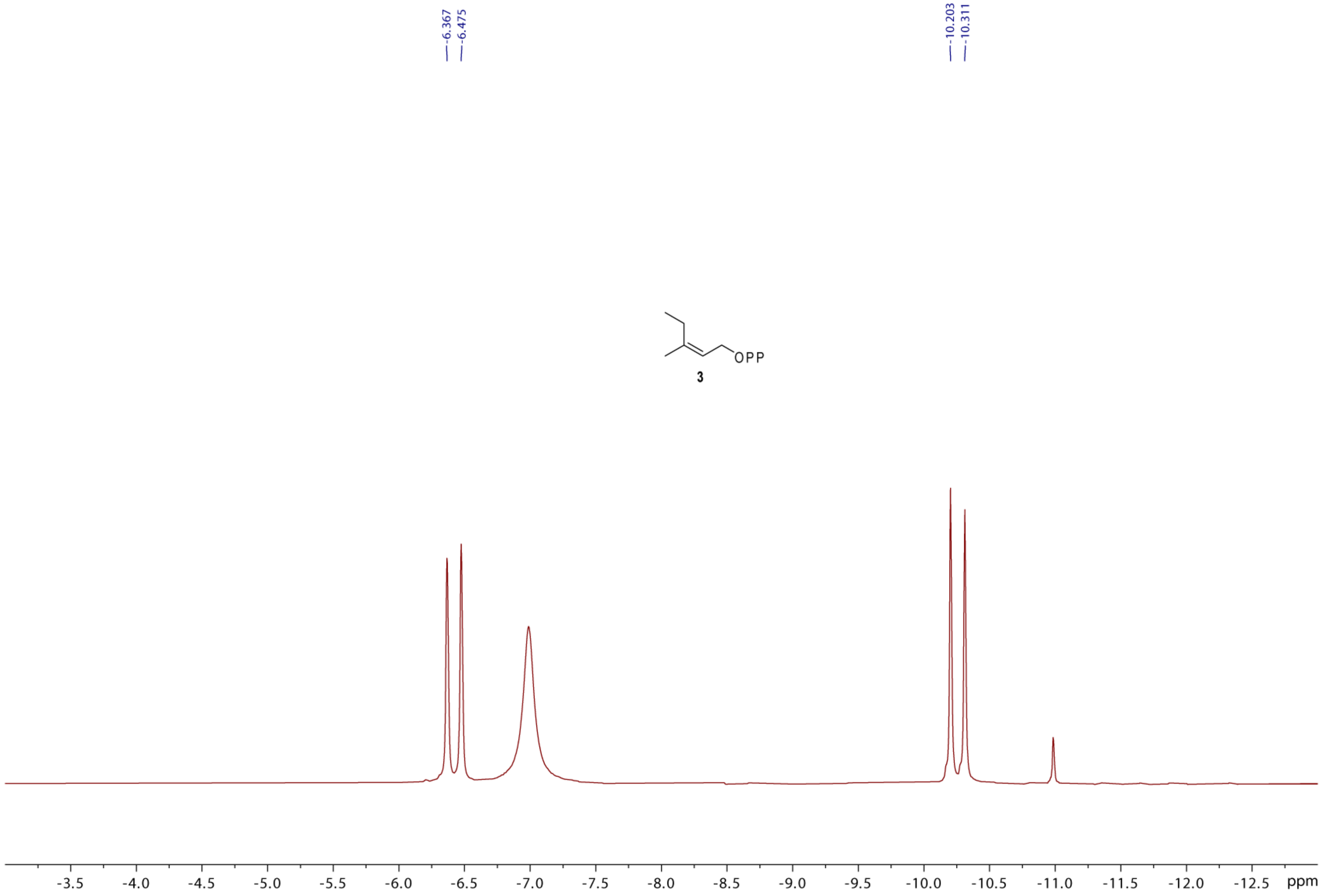

Figure S57. ^{31}P-NMR of **3** (202 MHz, D_2O).

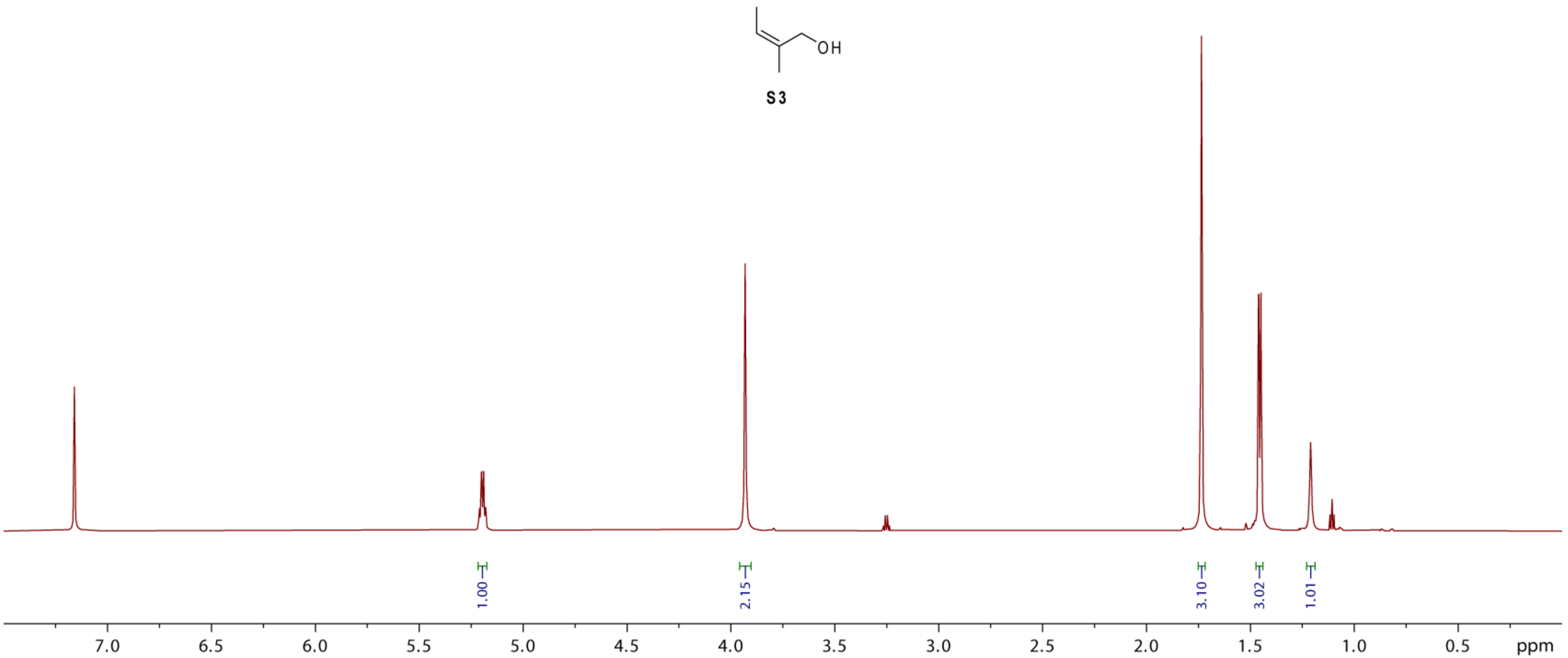

Figure S58. ^{1}H-NMR spectrum of **S3** (700 MHz, C_6D_6).

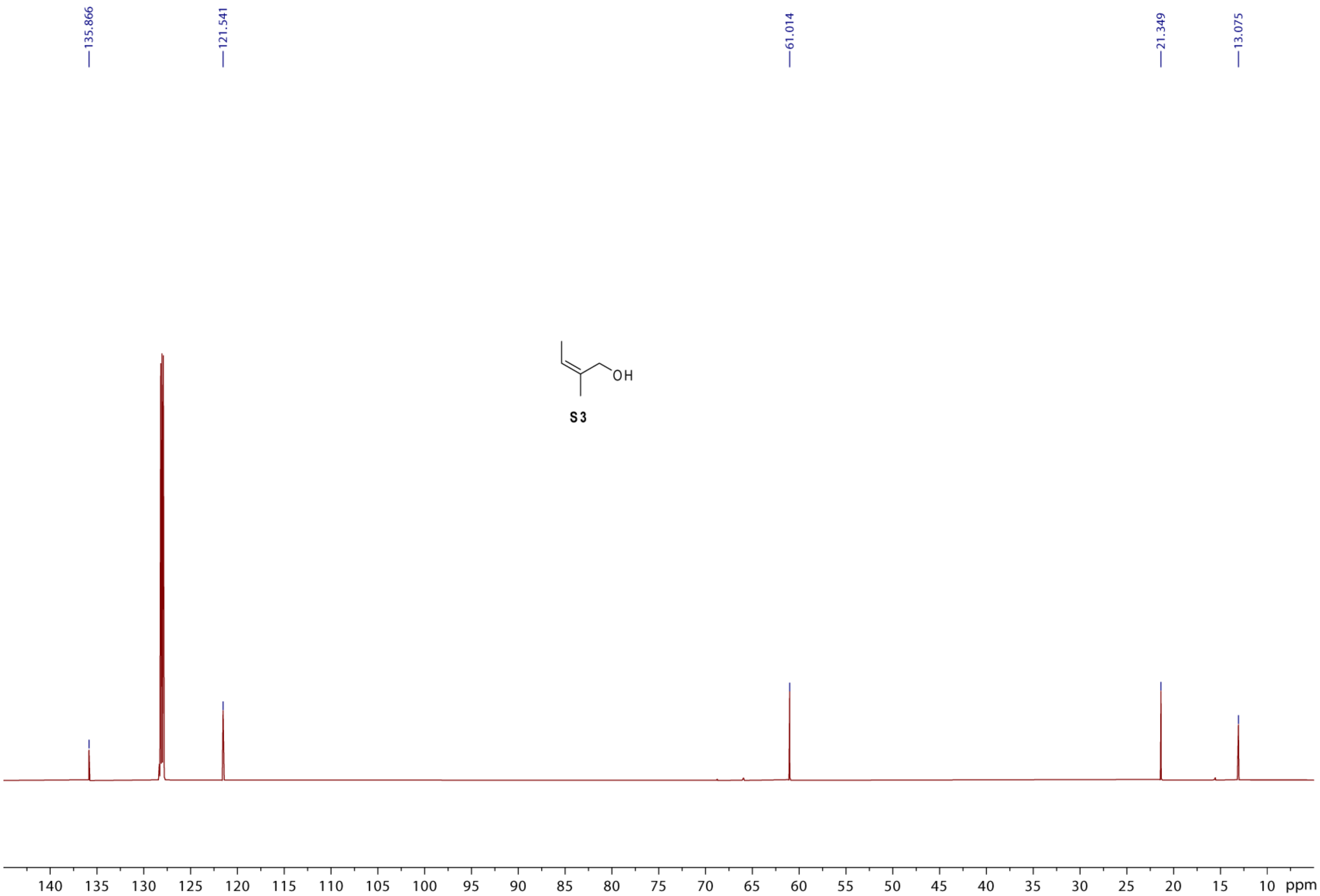

Figure S59. ^{13}C-NMR spectrum of **S3** (176 MHz, C_6D_6).

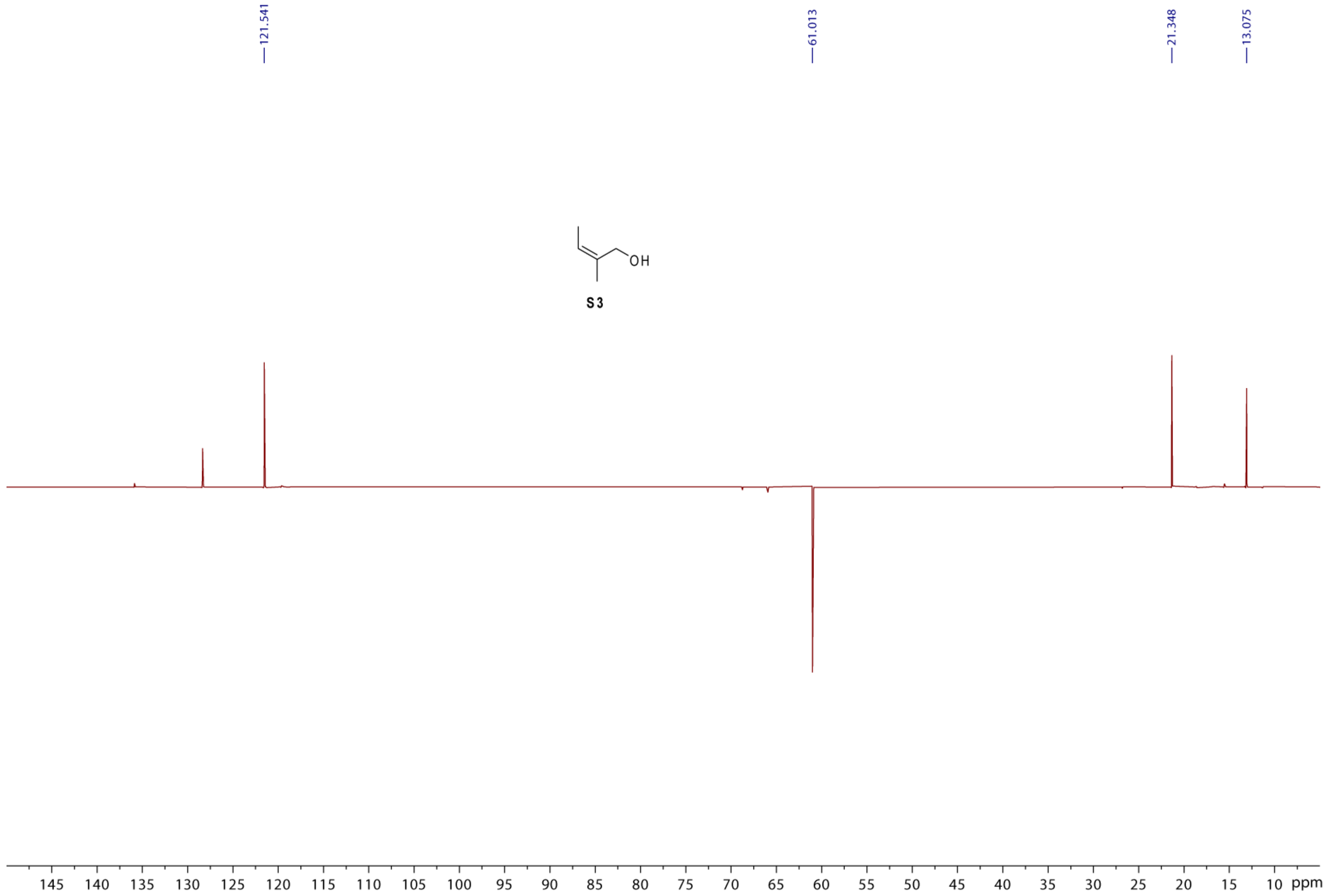

Figure S60. ^{13}C-DEPT spectrum of **S3** (176 MHz, C_6D_6).

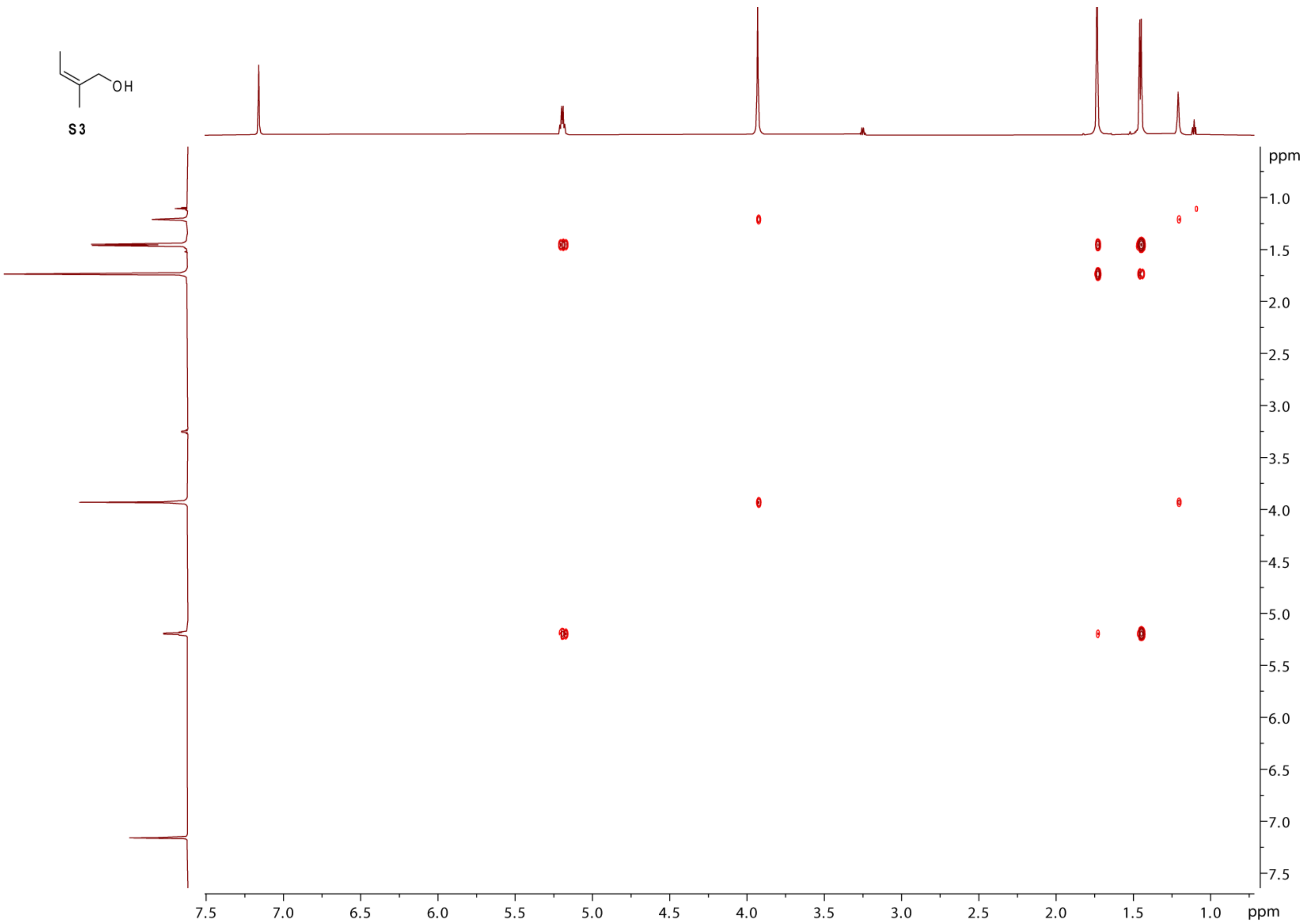

Figure S61. ^{1}H-^{1}H-COSY spectrum of **S3** (700 MHz, C_6D_6).

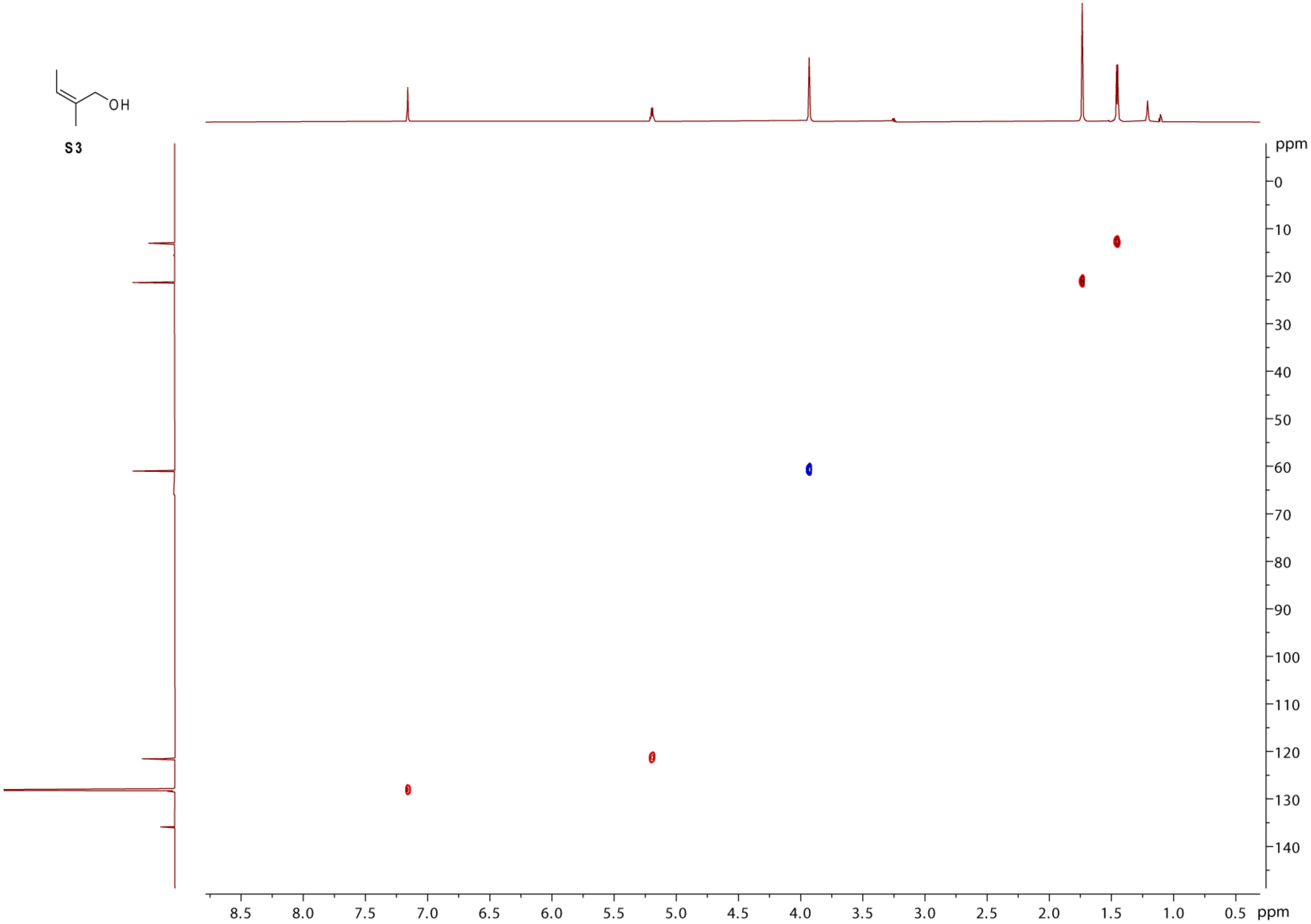

S3

Figure S62. HSQC spectrum of **S3** (C_6D_6).

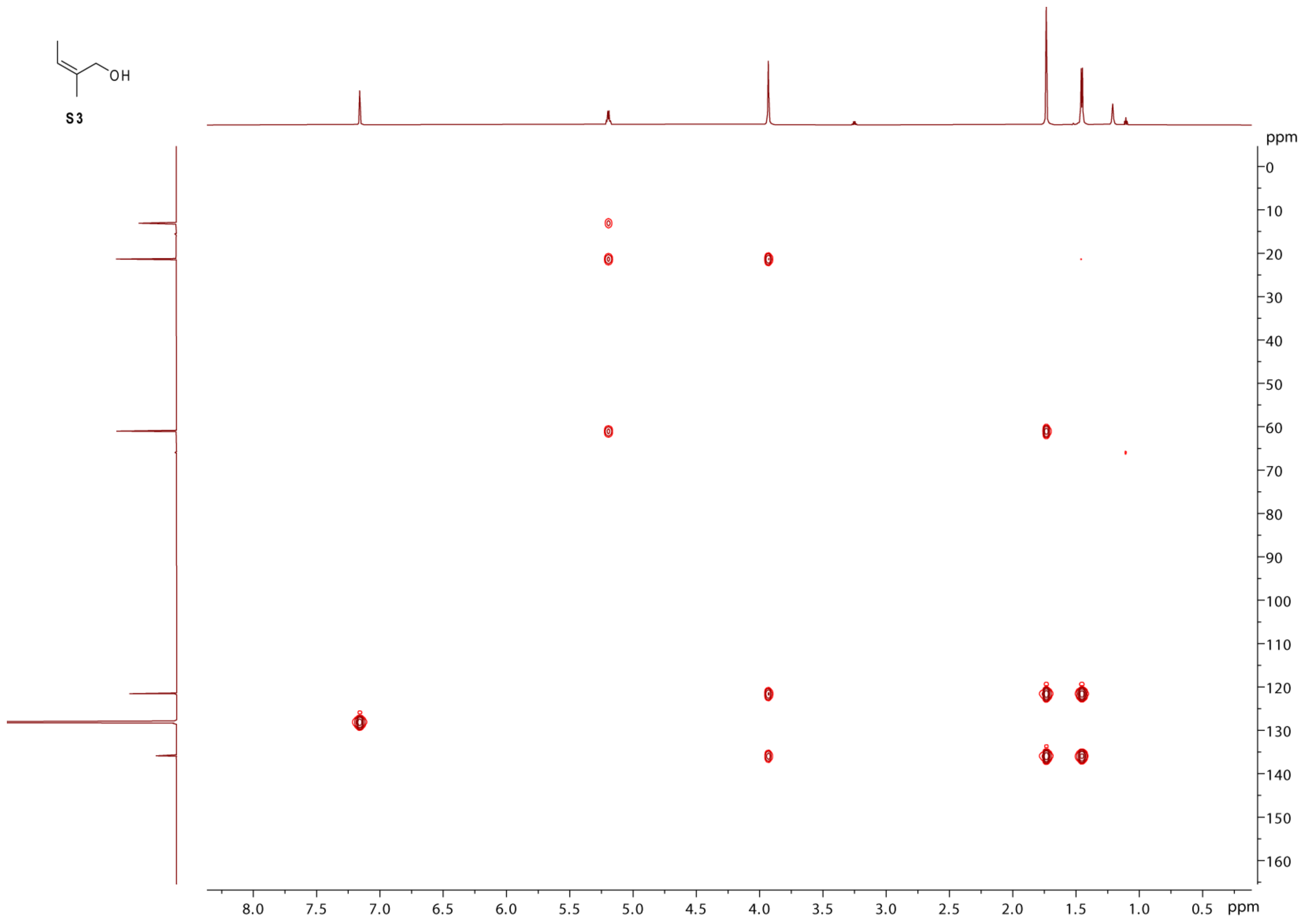

Figure S63. HMBC spectrum of **S3** (C_6D_6).

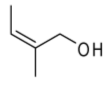

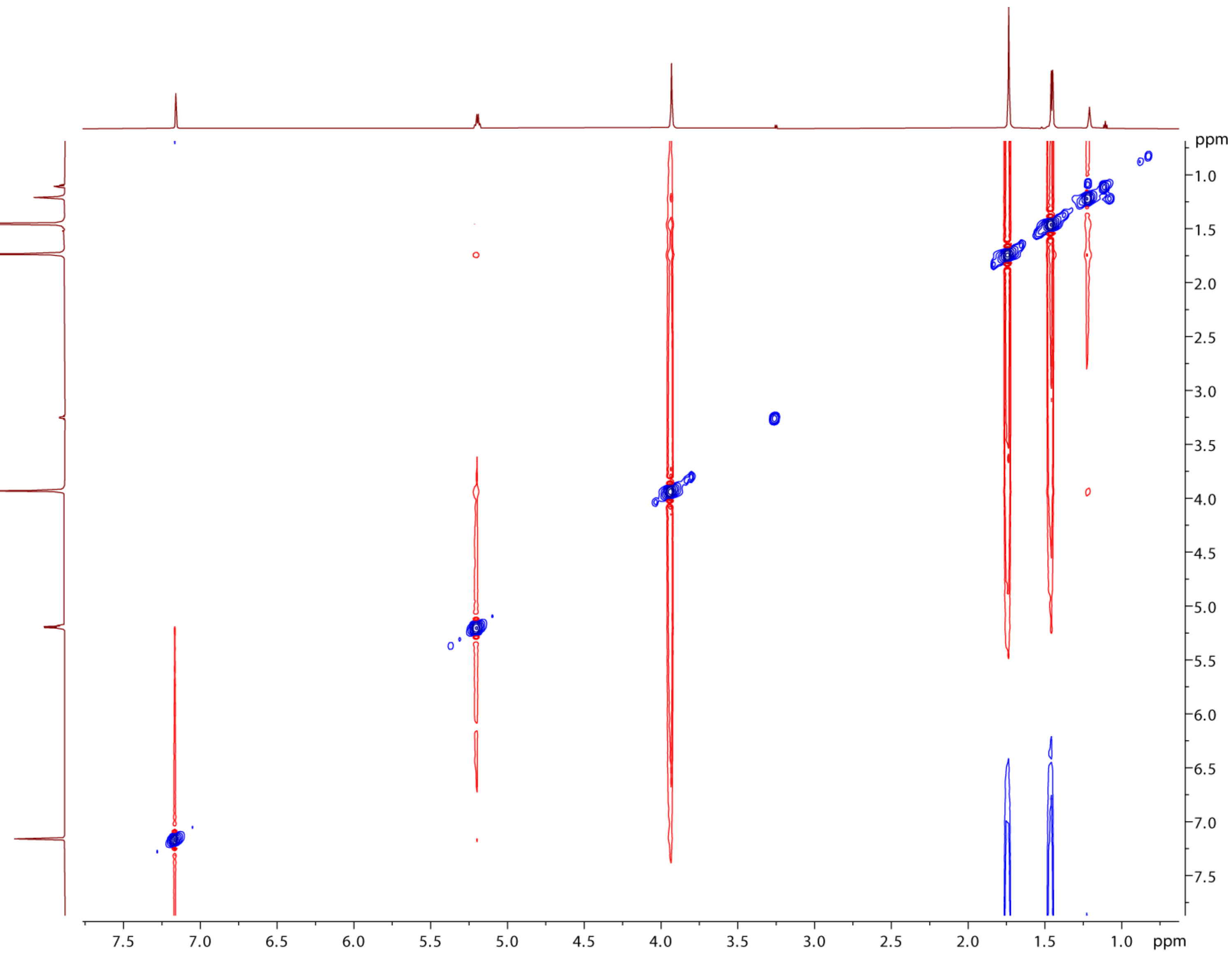

Figure S64. NOESY spectrum of **S3** (C_6D_6).

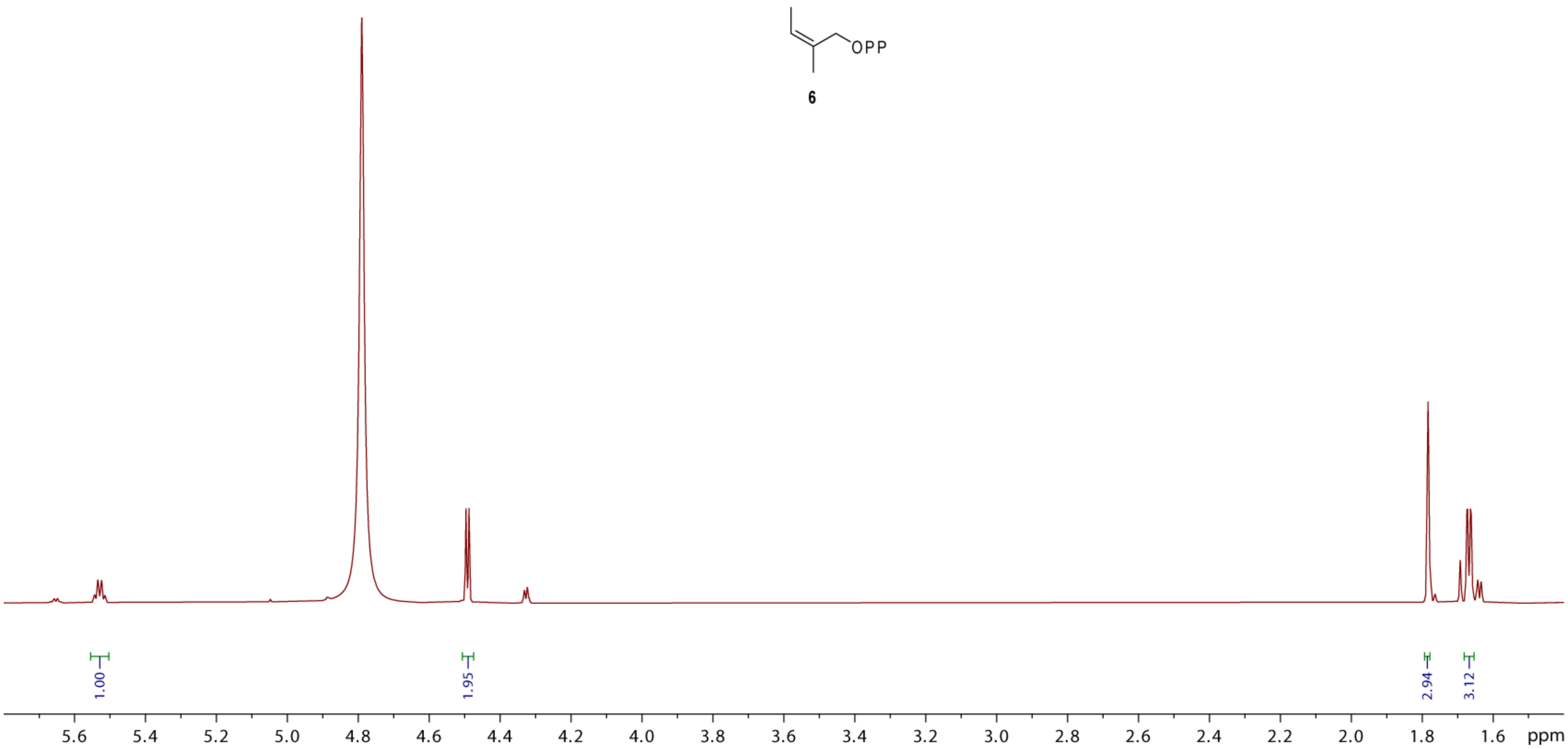

Figure S65. ^{1}H-NMR spectrum of **6** (700 MHz, D_2O).

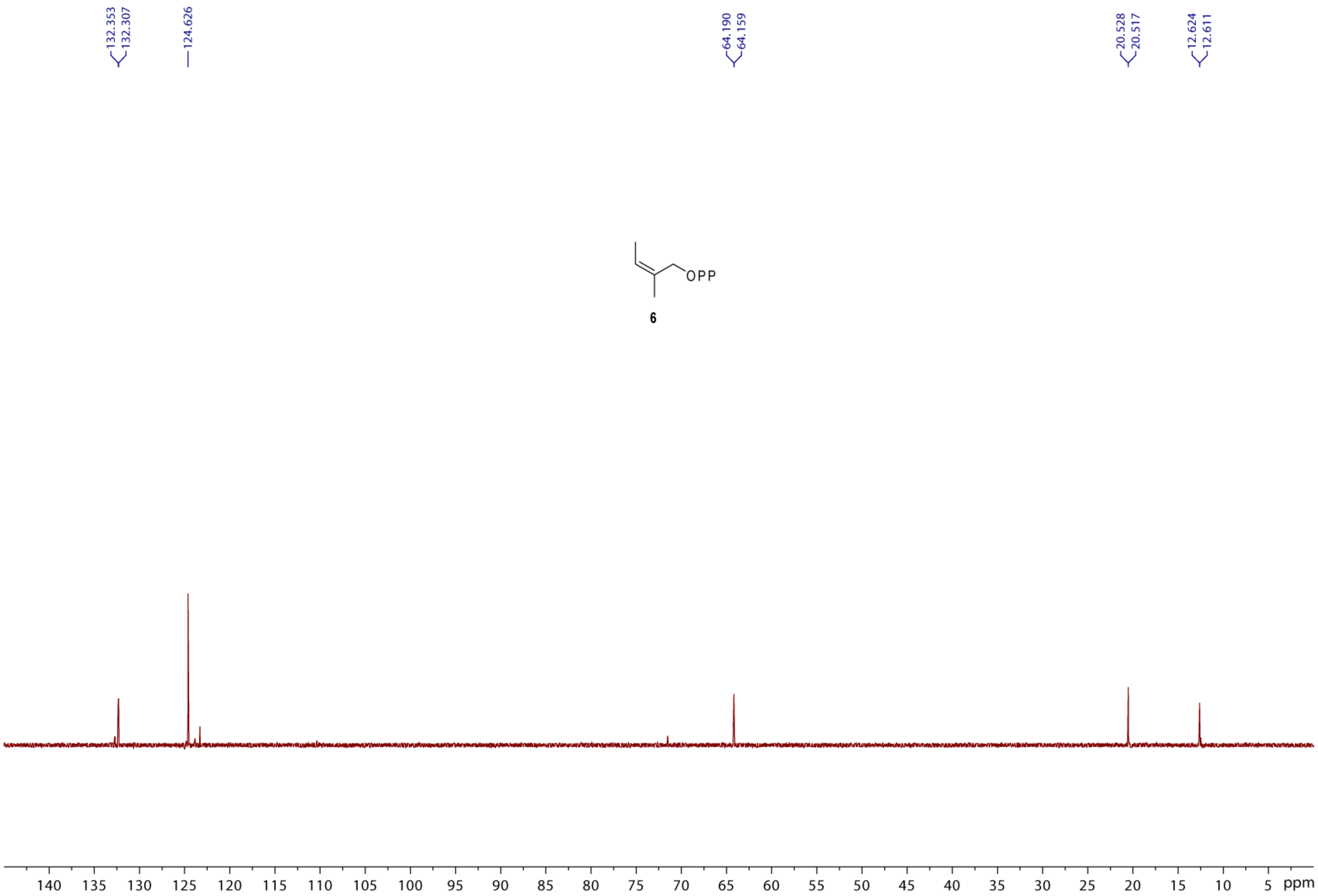

Figure S66. ^{13}C-NMR spectrum of **6** (176 MHz, D_2O).

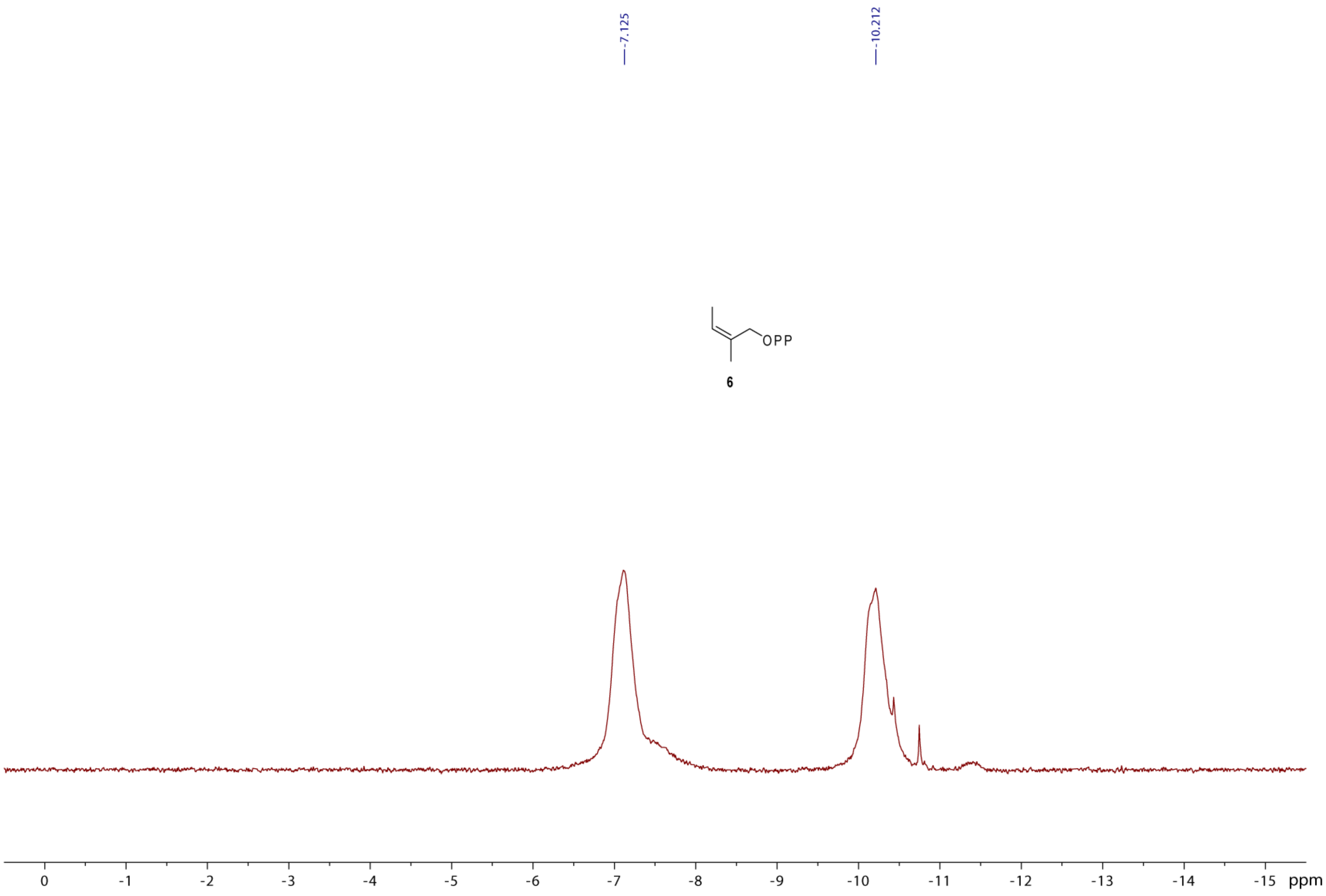

Figure S67. ^{31}P-NMR of **6** (202 MHz, D_2O).

References

[1] J. Rinkel, S. T. Steiner, G. Bian, R. Chen, T. Liu, J. S. Dickschat, *ChemBioChem* **2020**, *21*, 486.

[2] N. Shangguan, M. Joullié, *Tetrahedron Lett.* **2009**, *50*, 6748.

[3] K. Ogura, T. Nishino, T. Koyama, S. Seto, *J. Am. Chem. Soc.* **1970**, *92*, 6036.

[4] S. E. Sen, G. J. Ewing, *J. Org. Chem.* **1997**, *62*, 3529.

[5] L. A. Johnson, A. Dunbabin, J. C. R. Benton, R. J. Mart, R. K. Allemann, *Angew. Chem. Int. Ed.* **2020**, *59*, 8486.
